# Changes in the Immune Phenotype and Gene Expression Profile Driven by a Novel Tuberculosis Nanovaccine: Short and Long-Term Post-immunization

**DOI:** 10.3389/fimmu.2020.589863

**Published:** 2021-01-28

**Authors:** Amparo Martínez-Pérez, Ana Igea, Olivia Estévez, Catarina M. Ferreira, Egídio Torrado, António Gil Castro, Carmen Fernández, Anna-Lena Spetz, Lucille Adam, Moisés López González, Mahavir Singh, Rajko Reljic, África González-Fernández

**Affiliations:** ^1^ Immunology Group, CINBIO, Universidade de Vigo, Vigo, Spain; ^2^ Galicia Sur Health Research Institute (IIS-GS), Hospital Alvaro Cunqueiro, Vigo, Spain; ^3^ Life and Health Sciences Research Institute, University of Minho, Braga, Portugal; ^4^ ICVS/3B’s-PT Government Associate Laboratory, Braga/Guimarães, Portugal; ^5^ Department of Molecular Biosciences, The Wenner-Gren Institute (MBW) Stockholm University, Stockholm, Sweden; ^6^ Lionex GmbH, Braunschweig, Germany; ^7^ Infection and Immunity Research Institute, St George’s, University of London, London, United Kingdom

**Keywords:** *Mycobacterium tuberculosis*, nanovaccines, immune protection, lung infection, transcriptomic analysis

## Abstract

Deciphering protection mechanisms against *Mycobacterium tuberculosis* (*Mtb*) remains a critical challenge for the development of new vaccines and therapies. We analyze the phenotypic and transcriptomic profile in lung of a novel tuberculosis (TB) nanoparticle-based boosting mucosal vaccine Nano-FP1, which combined to BCG priming conferred enhanced protection in mice challenged with low-dose *Mtb*. We analyzed the vaccine profile and efficacy at short (2 weeks), medium (7 weeks) and long term (11 weeks) post-vaccination, and compared it to ineffective Nano-FP2 vaccine. We observed several changes in the mouse lung environment by both nanovaccines, which are lost shortly after boosting. Additional boosting at long-term (14 weeks) recovered partially cell populations and transcriptomic profile, but not enough to enhance protection to infection. An increase in both total and resident memory CD4 and CD8 T cells, but no pro-inflammatory cytokine levels, were correlated with better protection. A unique gene expression pattern with differentially expressed genes revealed potential pathways associated to the immune defense against *Mtb*. Our findings provide an insight into the critical immune responses that need to be considered when assessing the effectiveness of a novel TB vaccine.

## Introduction

Despite being well-known and treated for years, tuberculosis (TB) is the leading cause of death from a single infectious pathogen worldwide. The World Health Organization estimates that one third of the world’s population carries the bacillus in a latent form, with a 10% probability of those infected to develop TB during their lifetime, and that contributes to 10 million new cases of TB occur yearly (1). Control of the global TB epidemic has been challenged by the lack of an effective vaccine. Bacille Calmette–Guérin (BCG) remains the only licensed TB vaccine, although its efficacy against the pulmonary form of TB in adulthood is highly variable (2, 3). Unfortunately, the development of novel effective vaccines is hampered by the limited knowledge we have of the mechanisms that provide protection against *Mycobacterium tuberculosis (Mtb)*.

The immune response against *Mtb* is complex and incompletely characterized. Although evidence supports the fundamental role of CD4^+^ T cells and cytokines (such as interferon gamma (IFNγ) (4, 5), tumor necrosis factor alfa (TNFα), interleukins 2 (IL-2) (6–9), and 12 (IL-12) (10, 11)) in TB, there are still no reliable correlates of protection. In this scenario, it becomes difficult to predict the outcome of the disease or to monitor the efficacy of novel vaccines.

Mucosal vaccination (12–18) and mucosal boosting of BCG, combining the overall protection conferred by BCG with the reinforcement of the mucosal immunity in the lungs (19–25), have been considered attractive strategies against pulmonary TB. Hart et al. (26) demonstrated that the combination of subcutaneous BCG plus two intranasal boosts of a novel nanovaccine, Nano-FP1, significantly reduced the bacterial burden in mouse lungs after TB infection. Nano-FP1 is based on nanoparticles produced by the emulsification of yellow carnauba (YC) palm wax with sodium myristate (NaMA), coated with a fusion protein made of three different antigens of *Mtb*: the secreted protein Ag85B, the 16-kDa latency induced protein alpha crystalline (Acr) and the heparin-binding hemagglutinin (HBHA). Similar boosting nanovaccines based on other antigen combinations were also tested, but they did not show improved efficacy against *Mtb* (R. Reljic, unpublished). Among them is the Nano-FP2 vaccine, which displayed one single antigen replacement in its fusion protein compared to Nano-FP1, with antigen Ag85b replaced by the Mannose Binding Protein 64 (MPT64).

We intend to identify a phenotypic and/or transcriptomic profile of the efficacy of the Nano-FP1 TB vaccine in mice. Herein, we performed a systematic analysis of the novel Nano-FP1 prototype, analyzing the cellular signature and gene expression profile triggered in the pulmonary environment. Nano-FP2 was also tested as an example on a non-protective vaccine candidate. The effect produced by the intranasal boost with Nano-FP1 on previously BCG-immunized mice was evaluated at short-term (2 weeks), medium-term (7 weeks), and long-term (11 weeks) intervals. We found a unique cellular and transcriptional profile at short-term, characterized by alterations in CD4^+^ T cell populations and marked changes in gene expression. Nonetheless, we observed that the boosting effect was transient and it did not trigger an effective immunological memory against TB long term. Our findings suggest a critical role for the long-lived CD4+ T cell immunity that should be mandatory when assessing the effectiveness of a novel TB vaccine.

## Materials and Methods

### Mice

Six-week-old female specific pathogen-free C57BL/6 mice were purchased from Envigo (Spain). The mice were maintained under barrier conditions in a BL-3 biohazard animal facility at the University of Minho, Braga, Portugal, with constant temperature (24 ± 1°C) and humidity (50 ± 5%). The animals were fed a sterile commercial mouse diet and provided with water *ad libitum* under standardized light-controlled conditions (12 h light and dark periods). The mice were monitored daily, and none of the mice exhibited any clinical symptoms or illness during this experiment.

For the early response experiment, 6-week-old female specific pathogen-free C57BL/6 mice were purchased from Scanbur (Denmark), and housed in pathogen- free conditions at the Animal Department of MBW, Stockholm University, Sweden. Mice were acclimatized for at least 1 week before use and supervised daily. The specified pathogen free condition of the facility was confirmed by continuous use of sentinel mice.

All animal experiments were performed with ethical approval from the hosting institutions and according the national regulations and legislation of that country.

The study was approved by and performed in accordance with guidelines of the CEEA Xunta de Galicia, code ES-360570215601/17/INV. MED.02.OUTROS04/AGF/02. Early response experiments were approved by and performed in accordance with guidelines of the Stockholm North Ethical Committee on Animal Experiments, permit number N170/15.

### Nanovaccines Formulation

Two different vaccine candidates were used as intranasal (in) boost to BCG, hereafter referred to as Nano-FP1 and Nano-FP2. Both candidates consist of a combination of yellow carnauba palm wax with sodium myristate (YC-NaMA) nanoparticles (NPs) (Bethlehem, PA, USA) and a fusion protein (FP) composed of an N-terminal histidine tag and the *Mtb* antigens Acr (Rv2031c), Ag85B (Rv1886c), and the heparin-binding domain of HBHA (Rv0475) (FP1) or MPT64 (Rv1980c), Acr and HBHA (FP2). The vaccine formulation included 0.1% Yc-NaMA NPs, 200 µg/ml of the corresponding FP and 400 µg/ml PolyI:C (Sigma Aldrich) in saline solution, with 50 µl delivered to the each mouse. Nano-FP1 was used as the prototype of interest, taking into account previous studies reporting its protective effect (26) and Nano-FP2 was used as a representative of a nonprotective vaccine candidate (data not shown).

### Study Groups and Immunization Protocol

**Figure 1:**
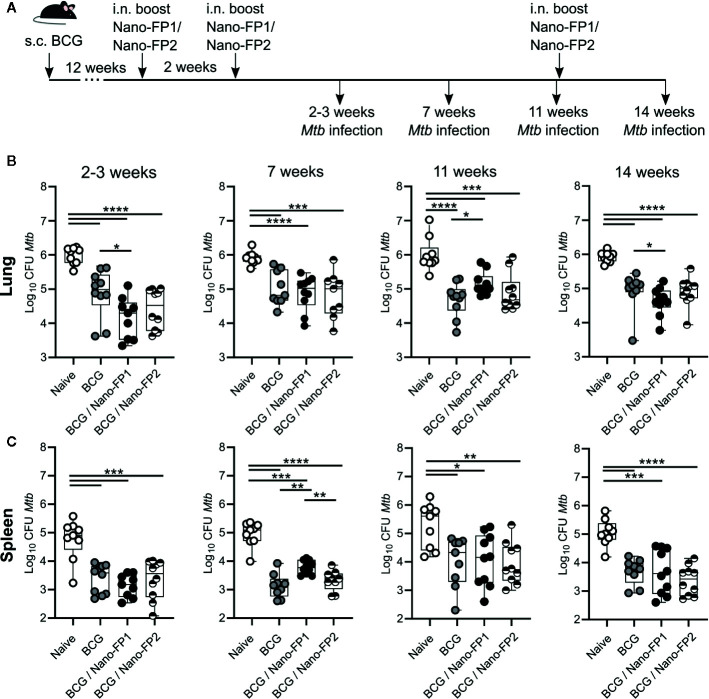
CFUs measurement in lung and spleen of *Mtb* infected mice. **(A)** Mice were left unvaccinated or vaccinated either with BCG alone (BCG) or BCG followed by intranasal Nano-FP1 (BCG/Nano-FP1) or Nano-FP2 (BCG/Nano-FP2). At the points indicated after the second boost, mice were infected with *Mtb* through the aerosol route. Lungs and spleen were collected 30 days after infection and plated to assess bacterial burdens in all groups, as described in Material and Methods (n = 9–10 mice per group). *Mtb* colony-forming units (CFUs) were determined in the lungs **(B)** and spleen **(C)**. Mann–Whitney-Wilcoxon test was used for statistical analysis. *p < 0.05; **p < 0.01; ***p < 0.001; ****p < 0.0001.

**Figure 2:**
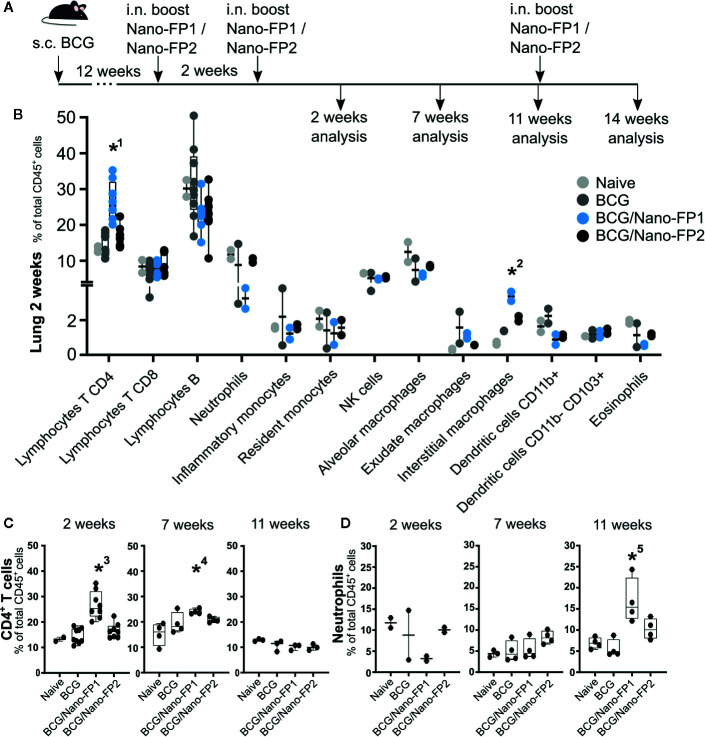
Immune cell populations in lung. **(A)** Groups of mice were vaccinated as described in **Figure 1**. **(B)** Lung immune cell populations at 2 weeks. **(C)** Percentages of selected immune populations CD45+ CD4^+^ T cells and **(D)** neutrophils in lungs analyzed at different time points (2, 7, 11 and 14 weeks). Data represent percentages of each cell population referred to the total of immune CD45-positive cells. *1 and *3: The percentage (%) of CD4^+^ T in the BCG/Nano-FP1 group was significantly higher than those in Naive, BCG and BCG/Nano-FP2 groups at 2 weeks. *2: The % of interstitial macrophages was significantly higher than that one in Naive group at 2 weeks. *4: The % CD4^+^ T cells was significantly higher than that one in Naive group at 7 weeks.*5: The % of neutrophils was significantly higher than that one in BCG group at 11 weeks. Kruskal–Wallis test and Dunn’s multiple comparisons test were used for statistical analysis. *p < 0.05.

The vaccination groups and schedules are shown in **Figures 1A** and **2A**. For subcutaneous (s.c.) priming vaccination, mice received 0.5 million CFUs of BCG strain Pasteur. Twelve and 14 weeks later, mice from the Nano-FP1 and Nano-FP2 groups were anesthetized with 100 µl of ketamine–xylazine and 50 µl of the corresponding nanovaccine was intranasally administered. A group of mice were also administered a 3rd boost of the corresponding nanovaccine 25 weeks after sc. BCG. Animals were divided in four experimental groups: Unvaccinated mice (henceforth referred to as Naive group); mice vaccinated with subcutaneous (s.c.) BCG alone (BCG group); mice vaccinated with BCG s.c. and 12 weeks later an intranasal boost with Nano-FP1 (BCG/Nano-FP1 group) or Nano-FP2 (BCG/Nano-FP2 group). Animals were studied at different time points (2, 7, and 11 weeks (after two intranasal challenges), and 14 weeks (after an additional third intranasal challenge).

For the early immune experiment (24 h), mice were subcutaneously vaccinated with either 0.5 million CFUs of BCG or PBS. Twelve weeks later, they were administered intranasally one dose of either Nano-FP1 or PBS. 24 h later, mice were sacrificed. Animals were divided in four experimental groups (1): mice receiving s.c and intranasal PBS as control (herein referred as PBS/PBS-24h); (2) only vaccinated with BCG s.c. (BCG/PBS-24h), (3) only with an intranasal boost of Nano-FP1 (PBS/Nano-FP1-24h), and (4) animals received BCG s.c. and twelve weeks later one intranasal dose of Nano-FP1 (BCG/Nano-FP1-24h).

### Bacteria

The H37Rv strain of *M. tuberculosis* was grown in Middlebrook 7H9 liquid medium (BD Biosciences, San Diego, CA) for 7–10 days and then sub-cultured in Proskauer Beck (PB) medium supplemented with 0.05% Tween 80 and 2% glycerol, until the mid-log phase. Bacterial stocks were aliquoted and stored at −80°C. Bacterial frozen stocks were used to infect mice *via* aerosol route, using a Glas-Col inhalation exposure system. Bacterial clumps were disrupted by forcing them through a 26G needle before diluting the bacterial suspension in water (Aqua B. Braun) to a concentration of 2 × 10^6^ CFUs/ml to deliver 100 CFUs into the lungs.

### Infection and Sample Collection

Mice were challenged *via* the aerosol route with the H37Rv strain at different time points (2–3, 7, and 11 weeks) following the last boost of the intranasal nanovaccines. Sample collection was conducted both pre- and post-challenge for each experimental group. Mice from the “pre-challenge” group were euthanized with CO_2_ and lung parenchyma, bronchoalveolar lavage (BAL) and spleen were collected for analysis. The remaining animals (“post-challenge” group) were sacrificed four weeks after infection on each of the corresponding time points for organ CFU count. Lung parenchyma and spleen were collected from these mice for immunological assays.

### Sample Processing

BAL was collected by irrigating lungs *via* trachea with a syringe containing 1 ml of cold PBS. Lungs and spleen were aseptically removed after BAL lavage and were homogenized and processed for immunological assays. Prior to homogenization, lungs were incubated in digestion medium DMEM (Dulbecco’s Modified Eagle Medium, High glucose NEAA, no glutamine, Gibco) supplemented with collagenase 0.15 mg/ml (Sigma Aldrich) at 37°C for 30 min. Spleens and collagenase-incubated lungs were homogenized and filtered through a 40 μm nylon mesh cell strainer (BD Biosciences, San Diego, CA) to obtain a homogenous cell suspension. BAL, lung and spleen cells were treated with red blood cell (RBC) lysing buffer (0.87% of NH_4_Cl solution and 5% of PBS in water) for 5 min, and washed twice with DMEM supplemented with 10% of heat-inactivated fetal bovine serum. Leukocyte fraction was isolated by density gradient centrifugation on an 80%/40% Percoll (GE Healthcare, Sigma Aldrich) gradient. In mice sacrificed post-challenge, the left lung and half spleen were reserved for organ CFU count.

### Bacterial Counts

The number of viable bacteria in lung and spleen from infected mice was determined by plating serial dilutions of the organ (left lung or half spleen) homogenates onto Middlebrook 7H11 agar (Difco Laboratories) supplemented with 10% OADC (Difco Laboratories). Colonies were counted after 3 weeks of incubation at 37°C with 5% CO_2_ atmosphere.

### Antibodies and Surface Staining

For analysis of general immune populations and lymphocyte composition in parenchyma lung and BAL, cell pools from 8 to 12 mice per group were used for the 2-week analysis and four mice per group for the 7, 12, and 14 weeks analysis.

Cells were incubated for 30 min with antibodies at 4°C, washed with FACs buffer (PBS with 3% FBS and 0.1% of 10 mM sodium azide) and kept at 4°C until flow cytometry analysis. List of antibodies used and references can be found in **Table S1**.

In the analysis of general immune populations, we identified CD4^+^ T lymphocytes (CD45+ CD3+ CD4+ cells), CD8^+^ T lymphocytes (CD45+ CD3+ CD8+ cells), B lymphocytes (CD45+ CD3- CD19+ cells), neutrophils (CD45+ Ly6G+ CD11b+), inflammatory monocytes (CD45+ Ly6G− MHCII− CD64+ CD11b+ Ly6C+), resident monocytes (CD45+ Ly6G− MHCII− CD64+ CD11b+ CD11c+), NK cells (CD45+ Ly6G− MHCII− CD64− CD11b+), alveolar macrophages (CD45+ Ly6G-CD64+ Siglec-F+), exudate macrophages (CD45+ Ly6G-CD64+ Siglec-F− Ly6C+), interstitial macrophages (CD45+ Ly6G-CD64+ Siglec-F− Ly6C−), CD11b+ dendritic cells (CD45+ Ly6G− CD64− CD24+ MHCII+ CD11b+), CD11b− CD103+ dendritic cells (CD45+ Ly6G− CD64− CD24+ MHCII+ CD11b− CD103+) and eosinophils (CD45+ Ly6G− CD64− CD24+ MHCII− CD11b+) in lung, following cytometry gating strategy described in (27) and depicted in (**Supplementary Figure S1**).

For lymphocyte composition analysis, we identified central memory cells (CD45+ CD3+ CD4+/CD8+ CD44+ CD62Lhigh CD127+), effector memory cells (CD45+ CD3+ CD4+/CD8+ CD44+ CD62Llow CD127+), effector cells (CD45+ CD3+ CD4+/CD8+ CD44+ CD62Llow CD127−) and lung resident memory cells (CD45+ CD3+ [CD4+ CD44+ CD62Llow CD69+] or [CD8+ CD44+ CD62Llow CD69+ CD103+]) following cytometry gating strategy depicted in (**Supplementary Figure S1**).

### Intracellular Cytokine Staining

Cell pools from 8–12 mice per group were used for the 2 weeks analysis and three to four mice per group for the 7, 12, and 14 weeks analysis. Before intracellular cytokine staining, single cell suspensions (obtained from lung or BAL) from immunized animals (1 × 10^6^ cells) were stimulated for 5 h as follows: BCG group with Ag85 (5 µg/ml); BCG/Nano-FP1 group with Ag85 (5 µg/ml) and FP1 (5 µg/ml); BCG/Nano-FP2 group with Ag85 (5 µg/ml) and FP2 (5 µg/ml). 90 min after stimulation, 10 ng/µl of Brefeldin A (Sigma Aldrich) was added to the cells, and incubated for 3 h at 37°C to avoid cytokine release into the culture media. Cells were stained for 30 min at 4°C with antibodies directed to surface antigens in FACs buffer. Cells were then fixed and permeabilized with FACs buffer with 0.05% Saponin (Sigma Aldrich). Intracellular cytokine staining was performed staining the cells with the intracellular antibodies for 30 min at 4°C. After that, cells were washed with FACs buffer and kept at 4°C until flow cytometry analysis, following cytometry gating strategy depicted in (**Supplementary Figure S1**). List of antibodies used and references can be found in **Supplementary Table S1**.

### Flow Cytometry and Data Analysis

Samples were run on an LSRII flow cytometer (BD Bioscience), and data were analyzed using Flowlogic (version 7.1, FlowLogic; UK) software. Graphpad Prism version 6.00 for Windows (GraphPad Software; CA, USA) was used for statistical analysis and graph representation.

### RNA Sequencing

RNA from BAL cells and immune lung infiltrate was extracted using RNeasy Plus Micro Kit (Qiagen) according to the manufacturer’s recommendations. For early response experiments, RNA was extracted from the post-caval lung lobes using the RNeasy Plus Mini kit (Qiagen) according to the manufacturer’s recommendations. RNA quality was assessed based on RIN value using Agilent 2100 Bioanalyzer and the Agilent RNA 600 Nano Kit (Agilent Technologies). Three samples of pooled RNA were analyzed per condition, selecting those with higher RIN value and RNA concentration. In the case of mice sacrificed at 11 and 14 weeks, two BAL samples were analyzed due to low RNA amount.

RNA sequencing was performed on an Ion Proton sequencer (Ion Torrent, Thermo Fisher Scientific; CA, USA) at the Genomic Service at the Centro de Apoio Científico-Tecnolóxico á Investigación of University of Vigo (CACTI). Poly(A)-mRNA fraction was enriched using the Dynabeads^®^ mRNA DIRECTTM Micro Kit (Thermo Fisher Scientific). Enriched mRNA was processed with the Ion Total RNA-Seq Kit v2 (Life technologies-Thermo Fisher Scientific). cDNA libraries with a percentage of DNA in 50 to 160 bp less than 50% passed the quality control and were then used for template preparation. Enriched templates were loaded in an Ion PI Hi-Q Chef Kit PI chip and used for RNA-sequencing. Ion Proton Torrent Suite Software 5.4.0 filtered polyclonal reads (Ion Sphere Particles with >1 unique library template population), adapter dimers (reads where no or only a very short sequencing insert is present) and low quality reads (reads with unrecognizable key signal, low signal quality, and reads trimmed to < 25 bp). Usable raw data were recorded on FastQ files.

### RNA-seq Data Processing

FastQ files resulting from RNA-sequencing were processed using the computational resources of the Galician Supercomputational Centre (CESGA). FastQ files were analyzed using FastQC software to confirm they had an average per base Phred quality score 20 to 30. Index was generated using Rsem software (version 1.2.31) utilizing reference genome of *Mus musculus* Ensembl Version GRCm38 and gene transfer format (.gtf) annotation from Ensembl version GRCm38.90. Alignment and count quantification were performed using STAR (version 2.5) and Rsem (version 1.2.31) software respectively.

### Differential Gene Expression Analysis

Differential gene expression between groups was assessed using DESeq2 R package (version 1.20.0). Study groups were compared to each other and the differential expression between groups was evaluated based on the adjusted p-value and the absolute log2 fold change. Benjamini Hochberg correction was used to obtain adjusted p-values. Genes with adjusted p-value < 0.05 and absolute log2 fold change ≥ 0.6 were considered significant in terms of differential expression.

#### Pathway Enrichment Analysis

Pathway enrichment analysis of significantly differentially expressed genes was performed using ReactomePA R package (version 1.26.0) (28). Pathways and biological processes with p value <0.05 were considered significantly enriched. Representation of enrichment analysis in clusters was performed using clusterProfiler R package (version 3.10.1) (29).

### Quantitative Real-Time PCR Analysis

RNA-seq results were validated by quantitative real-time PCR (RT-qPCR). Individual mice RNA were reverse transcribed to cDNA using SuperScript II Reverse Transcriptase (Invitrogen). cDNA and primers were mixed with PowerUp SYBR Green MadeMix (Applied Biosystems) and analyzed in a 7900HT Fast Real-Time PCR system (Applied Biosystems). A list of primers is described in **Supplementary Table S5**. Four independent biological replicates and technical triplicates of BCG/Nano-FP1 and BCG/Nano-FP2 groups were used for BAL analysis. Two to four independent biological replicates and technical triplicates of Naive, BCG, BCG/Nano-FP1, and BCG/Nano-FP2 groups were used for lung parenchyma analysis. The β-actin gene was used as internal control. Relative expression levels were calculated using the comparative method 2−ΔCt. Log2 ratios of fold change were calculated and compared in both RNA-Seq and RT-qPCR platforms.

## Results

### Memory After Vaccination With Bacille Calmette–Guérin/Nano-FP1: The Protective Effect After Boosting Decreases With Time

It was reported that a regimen of BCG vaccination followed by mucosal boosting with a novel nanovaccine Nano-FP1 provided enhanced protection against *Mtb* challenge compared to BCG alone (26). However, the *Mtb* challenge in these experiments was always scheduled 3 weeks after the second nanovaccine boost. Therefore, in this work we investigated the duration of protection provided by the Nano-FP1 nanovaccine. To do this, mice were vaccinated with BCG for 12 weeks at which point they received two i.n. boosts with the nanovaccine Nano-FP1 two weeks apart (**Figure 1A**). The non-protective Nano-FP2 (R. Reljic unpublished) was used as control. To determine the duration of protection conferred by the prime-boost regimen mice were challenged with *Mtb* through the aerosol route 2, 7, and 11 weeks after a second boost.

Our data confirmed that the combination of BCG priming followed by boosting with Nano-FP1 was more effective than only BCG in reducing the CFUs in lungs in mice infected 2 weeks after the second boost. On the other hand, BCG/Nano-FP2 did not improve upon the protection conferred by BCG alone. Nonetheless, the protection effect induced by the BCG/Nano-FP1 vaccine was lost after 7 and 11 weeks post vaccination (**Figure 1B**) indicating that protection conferred by this regimen is rapidly lost. To determine if memory could be recovered at long term, we administered an additional intranasal boost at 11 weeks with Nano-FP1 or Nano-FP2 vaccines. Although a reduction in the lung CFUs was observed in the BCG/Nano-FP1 group (0.39 log CFUs reduction on average in BCG/Nano-FP1 group vs BCG), it was not as strong as the protection achieved at 2 weeks (average 0.7 log CFUs reduction among BCG/Nano-FP1 and BCG alone at 2 weeks) (p value < 0.05) (**Figure 1B**).

CFUs analysis in spleen revealed that at 2 weeks no significant differences were found among groups receiving BCG/Nano-FP1 or BCG alone (**Figure 1C**). Strikingly, at 7 and 11 weeks the Nano-FP1 boost even hampered the observed BCG-systemic-protection, increasing significantly the bacterial burden in spleen.

Our results pointed that two intranasal boosts with Nano-FP1 in animals previously BCG vaccinated conferred enhanced lung protection to the *Mtb* infection at short-term compared to BCG alone. However, this protection was lost after 7 weeks and thereafter. Re-exposure by an extra vaccine boost reduced the CFUs, but to a lesser extent than expected after recalling immunological memory.

### Protection Induced by Bacille Calmette–Guérin/Nano-FP1 Associates With an Increase in CD4 T Cells and Interstitial Macrophages

With the aim to correlate the protection levels observed at the various time points with the immune signature, we used flow cytometry to define the immunophenotypic profile including T cell phenotype and cytokine production (**Figure 2A**).

First, we identified total lung CD4^+^, CD8^+^ and B lymphocytes, neutrophils, inflammatory and resident monocytes, NK cells, alveolar, interstitial and exudate macrophages, conventional dendritic and CD11b- CD103+ dendritic cells and eosinophils. From all the cell subsets analyzed, we only found statistically significant changes in the increased number of CD4^+^ T cells and interstitial macrophages in the BCG/Nano-FP1 group 2 weeks after the 2^nd^ nanovaccine boost (**Figure 2B**). We also detected a tendency of a lower percentage of neutrophils at this time, although these differences were not statistically significant.

We wanted to find out how these populations behave at long term in the lung, and found two features. First, the higher proportion of CD4^+^ T cells observed at 2 weeks in the BCG/Nano-FP1 group was partially lost at 7 weeks and completely gone at 11 weeks (**Figure 2C**). Secondly, at 11 weeks the percentage of neutrophils in the BCG/Nano-FP1 group increased abruptly (**Figure 2D**).

No significant differences in cell subsets were observed in lungs between the Naive and BCG groups at the time-points studied (**Supplementary Figure S2**).

### Bacille Calmette–Guérin/Nano-FP1 Induces an Increase in Resident Memory CD4 T Cells

Next, we focused on changes in the lymphocytic T cell sub-populations, analyzing the phenotype of T cell subtypes in lungs. These included the central memory, effector memory, effector and resident memory (RM) cells in both CD4^+^ and CD8^+^ subsets.

**Figure 3:**
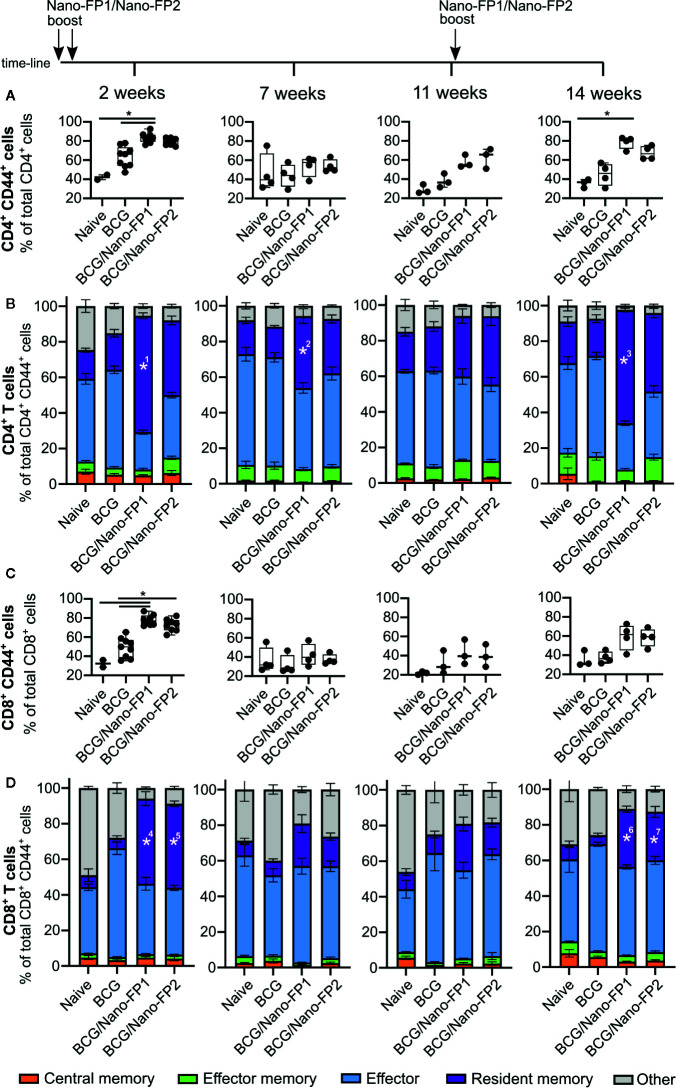
Analysis of CD4 and CD8 T cell subtypes in lung. Groups of mice were vaccinated as described in **Figure 1**. Cells were analyzed by flow cytometry at 2, 7 and 11 weeks after the 2^nd^ nanovaccine boost and at 14 weeks, 3 weeks after a third intranasal nanovaccine boost. **(A, C)** Data represent percentages of activated (CD44+) CD4 or CD8 T cells referred to the total of CD4^+^ or CD8^+^ T cells, respectively. **(B, D)** Graphs represent mean percentages (%) of T cell subtypes referred to the total of activated CD4^+^ or CD8^+^ T cells respectively ± standard error mean (SEM). Kruskal–Wallis test and Dunn*’*s multiple comparisons test were used for statistical analysis. *p < 0.05. *1: BCG/Nano-FP1*%* of resident memory CD4 T cells is significantly higher than those in Naive and BCG groups at 2 weeks. *2 and 3: BCG/Nano-FP1*%* of resident memory CD4 T cells are significantly higher than that those in BCG group at 7 and 14 weeks. *4, 5, 6 and 7: BCG/Nano-FP1 and BCG/Nano-FP2*%* of resident memory CD8 T cells are significantly higher than those in BCG group at 7 and 14 weeks.

A higher proportion of activated lymphocytes (CD4+ CD44+) in lungs of BCG/Nano-FP1 immunized mice at 2 weeks (**Figure 3A**) was observed, with an increasing significant proportion of CD4^+^ RM lymphocytes, when compared to the other groups (**Figure 3B**). This predominant CD4+ CD44+ feature was partially lost at 7 and 11 weeks, which also correlates with the lower level of protection against the *Mtb* infection at these time points. In contrast, after the administration of the 3^rd^ boost, CD4^+^ RM cells reached similar levels to those showed at short-term. BCG/Nano-FP2 also seemed to increase the proportion of activated CD4+CD44+ and CD4^+^ RM cells, although not reaching statistical significance.

Besides, both nanovaccines augmented the proportion of activated CD8+ CD44+ and CD8^+^ RM at short-term in a similar fashion (**Figures 3C, D**). Unlike CD4^+^ behavior, at 14 weeks we found a smaller proportion of CD8^+^ both activated and RM cells compared to 2 weeks. Moreover, no significant differences were observed between Naive and BCG mice at the time-points studied between different groups (data not shown). The immunophenotypic analyses showed that vaccination with BCG/Nano-FP1 induced significant changes in lung lymphocyte population at short-term, more pronounced than BCG/Nano-FP2, which were lost from 7 weeks and later on. We observed changes in both CD4^+^ activated lymphocytes and CD4^+^ RM cells. Also, administration of an extra boost of the nanovaccine recovered those lung cell populations in a similar manner.

### Bacille Calmette–Guérin/Nano-FP1 Induces Specific Profiles of Mono and Polyfunctional CD4 T Cells

In order to assess the profile of cytokines induced by the BCG/Nano-FP1 vaccine compared to the other vaccination regimes, lung and spleen cells were checked for specific activation following *in vitro* re-stimulation for 5 h with *Mtb* antigens. We studied the production of IFNγ, TNFα, IL-2, and IL-17 by intracellular staining in activated CD45+ CD3+ CD4+/CD8+ CD44+ cells.

**Figure 4:**
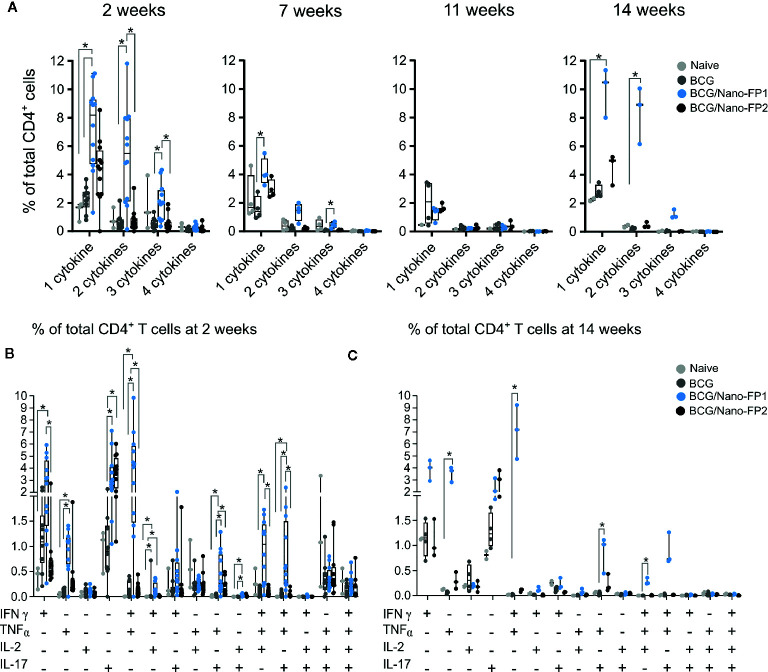
Analysis of CD4^+^ cytokine polyfunctionality in lung. Group of mice were vaccinated as described in **Figure 1**. Production of either INFɣ, TNFα, IL-2 and IL-17 or their combinations were analyzed at 2, 7 and 11 weeks after two intranasal nanovaccine boosts or at 14 weeks, three weeks after a third intranasal nanovaccine boost. **(A)** Data represent percentages of cytokine-producing activated CD4^+^ T cells at 2 or 14 weeks that produced 1 cytokine (INFɣ, TNFα, IL-2 or IL-17) or the combination of 2, 3, or 4 of those cytokines, referred to the total of CD4 T cells. **(B, C)** Data represent percentages of cytokine-producing activated CD4^+^ T cells at 2 **(B)** or 14 weeks **(C)** referred to the total of CD4^+^ T cells. Kruskal–Wallis test and Dunn*’*s multiple comparisons test were used for statistical analysis. *p < 0.05.

The number of CD4 cells producing one or combinations of cytokines was higher in the BCG/Nano-FP1 group at 2 weeks, but decreased at 7 weeks and even more at 11 weeks. However, the 3^rd^ boost increased again at 14 weeks the percentage of cells producing either one or more cytokines, resembling the initial 2 weeks levels (**Figure 4A**). Examining in detail the polyfunctional signature in mouse lungs at 2 weeks after receiving the Nano-FP1 vaccine, we found an increment of CD4^+^ T cells producing either IFNγ, TNFα, or IL-17, or combinations of IFNγ+ TNFα+ and IFNγ+ TNFα+ IL-2+ (**Figure 4B**), compared to the other groups.

At 14 weeks, after the extra boost, a similar profile to the 2-week signature was observed, with even higher percentages of TNFα+ and IFNγ+ TNFα+ producing cells (**Figure 4C**). Thus, the 2-week effect on secreting cytokines induced by BCG/Nano-FP1 can be rescued by an additional intranasal boost.

Regarding CD8^+^ T cells cytokine profile, we found that both nanovaccines Nano-FP1 and Nano-FP2 increased the percentage of cells producing IFNγ alone or in combination with TNFα in a similar manner (**Supplementary Figure S3**).

No significant differences were observed in lungs between Naive and BCG groups of mice at the studied time-points. Similarly, no significant differences were found between lung and bronchoalveolar cells, or splenocytes from the different groups (data not shown).

The outstanding CD4^+^ cytokine response following Nano-FP1 boosting at 2 and again at 14 weeks points toward a classical memory boosting effect that nonetheless does not enhance TB protection at long term.

### Early Lung Transcriptome Alterations Arise in Response to Nano-FP1

It is accepted that one of the most important factors in vaccination strategies is the election of effective adjuvant and delivery systems. To understand the possible causes of the short duration of protective and immunological memory induced by the BCG/Nano-FP1 vaccine, we first studied the ability of the nano-PolyIC system to create, in a very early phase, a lung environment able to support subsequent protective immune responses.

**Figure 5:**
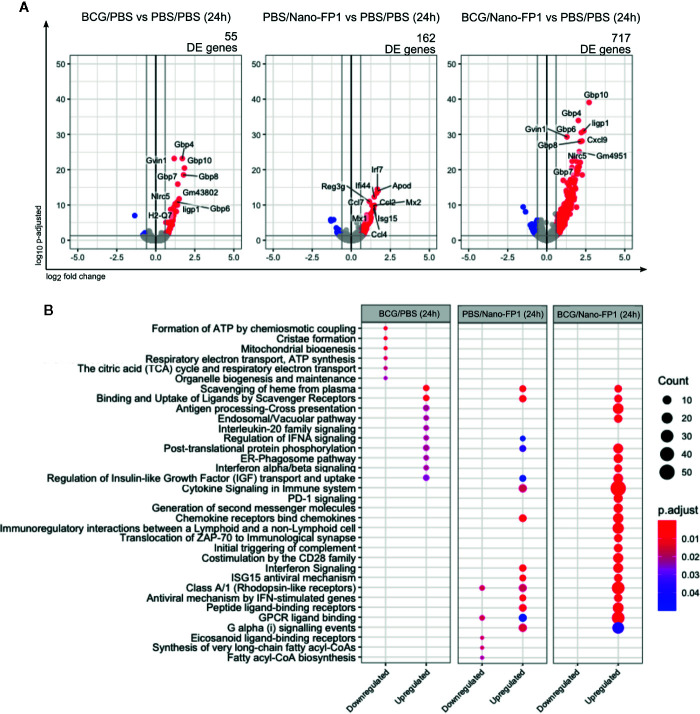
Transcriptomic changes in lung cells, 24 h after one intranasal nanovaccine boost. **(A)** Volcano plot representation of the differential expression analysis between experimental and control groups 24* h* after one boost with the BCG/Nano-FP1 vaccine. Animal received s.c. either PBS or BCG 12 weeks earlier, followed by one intranasal administration of either PBS (PBS/PBS and BCG/PBS groups, respectively) or Nano-FP1 vaccine (PBS/Nano-FP1 and BCG/Nano-FP1 groups, respectively). Two to three samples of pooled mice were analyzed per group. The differential expression analysis was made using DESeq2 R package, comparing all annotations of the reference genome (52636 annotations). Significantly DE genes (p adjusted < 0.05 and fold change ≥ 1.5) colored in red (upregulated) or blue (downregulated). Names of top-10 p-adjusted DE genes are plotted. **(B)** Pathway enrichment analysis of DE genes using ReactomePA package. Up and downregulated DE genes of each experimental groups were separated in clusters. Top-10 p-adjusted enriched pathways of each cluster of genes are represented. Count: number of DE genes involved in the pathway; *“*.

We analyzed mice lung transcriptome by RNA-Sequencing just 24 h after the boost. Rapid and substantial transcriptomic changes were observed after one single dose of Nano-FP1 intranasal administration. Differential gene expression analysis of each group versus control revealed a total of 55, 162 and 717 Differentially Expressed (DE) genes in BCG-24h, PBS/Nano-FP1-24h, and BCG/Nano-FP1-24h boosted groups, respectively (**Figure 5A**). This immediate effect points toward the mobilization of innate immune responses promoted by the vaccine delivery system. Remarkably, the considerably higher number of DE genes in BCG/Nano-FP1-24h mice reflects the importance of BCG priming in the outcome.

Analysis of enrichment pathways of up and downregulated DE genes, reflected how both BCG primed and non-primed Nano-FP1 shared some features, as upregulation of chemokine and Interferon signaling pathways (**Figure 5B**, **Supplementary Table S2**). Nonetheless, BCG/Nano-FP1-24h involved some unique pathways, related to antigen presentation, immunological interaction and synapses or triggering of complement, among others.

In summary, the analysis of changes induced by the BCG/Nano-FP1 vaccine at this early time point regarding various innate system related parameters, suggest a robust and powerful adjuvant effect.

### Bacille Calmette–Guérin/Nano-FP1 Induces Pronounced Changes in the Transcriptomic Profiles of Both Bronchoalveolar Lavage and Lung Shortly After Boosting

Our following steps comprised the RNA-Sequencing analysis of Naive, BCG, BCG/Nano-FP1 and BCG/Nano-FP2 vaccination groups at short and long-term (2, 11, and 14 weeks). The aim was to reveal possible unique transcriptomic changes correlated with the enhanced TB protection conferred by BCG/Nano-FP1 at 2 weeks and its subsequent disappearance. We analyzed independently bronchoalveolar lavage (BAL) cellular fraction and lung parenchyma to uncover potential specific mechanisms occurring in different lung compartments.

**Figure 6:**
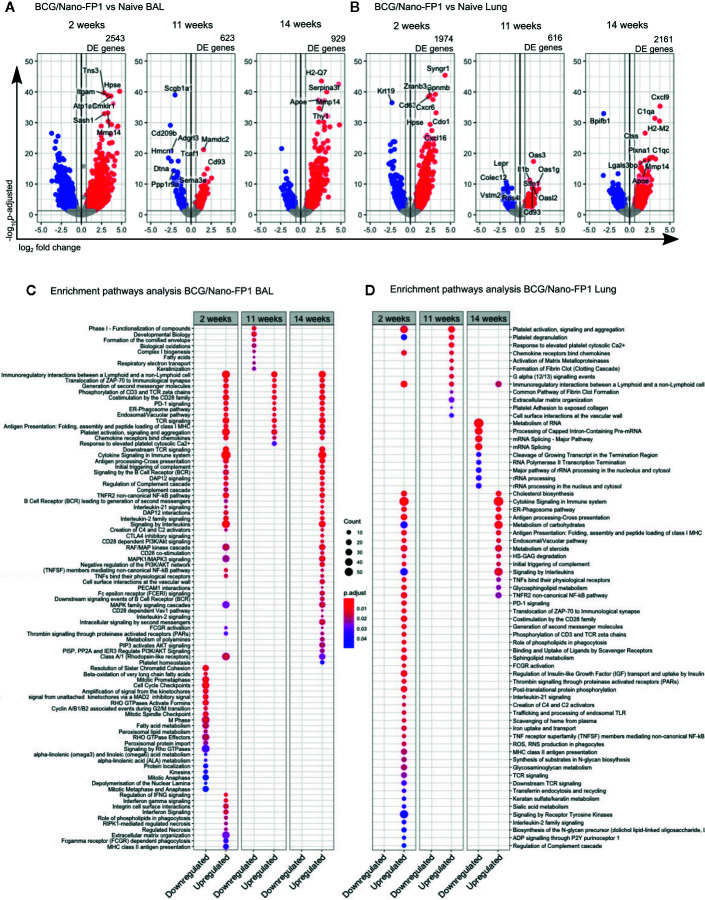
Transcriptomic changes in BAL and lung cells at short, long term and after re-immunization in the BCG/Nano-FP1 group, compared with naive mice. Animals in the BCG/Nano-FP1 group received s.c. BCG and 12^th^ weeks later, the nano-FP1 vaccine. They were studied at different time points (2 and 11 weeks (after two intranasal challenges), and 14 weeks (after an additional third intranasal challenge).Volcano plot representation of the differential expression analysis of genes obtained from the BCG/Nano-FP1 group, compared to control Naive group (unvaccinated) in bronchoalveolar lavage (BAL) **(A)** and lung parenchyma **(B)**. Three samples of individual or pooled mice were analyzed per group. The differential expression analysis was made using DESeq2 R package, comparing all annotations of the reference genome (52636 annotations). Significantly DE genes (p adjusted < 0.05 and fold change ≥ 1.5) colored in red (upregulated) or blue (downregulated). Names of top-10 p-adjusted DE genes are plotted. Pathway enrichment analysis of DE genes in BCG/Nano-FP1 compared to control Naive using ReactomePA package in BAL **(C)** and lung parenchyma **(D)**. Up and downregulated DE genes of each experimental groups were separated in clusters. Top-50 p-adjusted enriched pathways of each cluster of genes were represented. Count: number of DE genes involved in the pathway.

Differential gene expression analysis comparing BCG, BCG/Nano-FP1 or BCG/Nano-FP2 groups versus control group (Naïve), showed that at 2 weeks BCG/Nano-FP1 prompted major changes in the transcriptome, with 2,543 and 1,974 DE genes in BAL and lung, respectively (**Figure 6A**, **Supplementary Figures S4A, B**).

At 11 weeks, we found a different scenario. The number of DE genes in BCG/Nano-FP1 was markedly reduced to 623 and 616 genes in BAL and the lungs, respectively. After the 3^rd^ boosting (14 weeks), the number of DE genes in BCG/Nano-FP1 rose again, reaching 929 genes in BAL and 2161 DE genes in the lungs (**Figures 6A, B**). Moreover, we observed that this group conserved part of its DE genes at these time-points (**Supplementary Figures S4C, D**). The transcriptomic analysis comparing samples at 2 and 14 weeks shows that both shared the highest number of DE genes, with a total of 771 in BAL and 744 in the lungs (**Supplementary Figures S4C, D**).

Conversely, BCG/Nano-FP2 group displayed similar ciphers of DE genes at 2, 11, and 14 weeks, while BCG group showed the lowest number of DE genes compared to Naive in all studied time-points in both BAL and lungs (**Supplementary Figures S4A, B**).

### Biological Pathways Induced by Nano-FP1 Differ Over Time

We performed an enrichment pathway analysis to compare the biological context induced by BCG/Nano-FP1 vaccination, analyzing the up and downregulated DE genes at different time points, revealing some compelling details.

In BAL, several immune-related routes were upregulated in all, at 2, 11, and 14 weeks, such as: Immuno-regulatory interactions between a lymphoid and a non-lymphoid cell, immunological synapse, co-stimulation by CD28 family, PD-1 signaling, ER-Phagosome pathway, TCR, and chemokine signaling (**Figure 6C**).

Some pathways upregulated exclusively at 2 weeks were IFNγ signaling, Integrin cell surface interactions, extracellular matrix organization, RIPK1-mediated regulated necrosis, phagocytosis (Role of phospholipids and Fc gamma receptor dependent phagocytosis), or MHC-II antigen presentation.

Downregulated DE genes routes were mostly related with cell cycle and cell metabolism in the three clusters. Other pathways shared exclusively among 2 and 14 weeks involved *Cytokine*, *Interleukin*, *B cell receptor (BCR), DAP12 and NF-kB signaling, and the Complement cascade*.

In the case of lung parenchyma, part of the upregulated genes coincided with enriched pathways previously shared by 2 or 14 weeks in BAL, as *co-stimulation by CD28, TCR signaling, immunological synapse, or PD-1 signaling* (**Figure 6D**).

Other routes matched with those pathways occurring exclusively in BAL at 2 weeks, for example *MHC-II antigen presentation or role of phospholipids in phagocytosis*. Moreover, new unique pathways were found induced by the BCG/Nano-FP1 vaccine at 2 weeks in the lung, as *Reactive oxygen species (ROS) and Reactive nitrogen species (RNS) production in phagocytes*, *Scavenger receptors, endosomal TLR, signaling through P2Y receptors, Sphingolipid metabolism* or *regulation of Insulin-like Growth Factor*. Compared to BAL, a smaller fraction of pathways was shared among 2 and 14 weeks, including *Cholesterol biosynthesis, Cytokine and Interleukin signaling*, *ER-Phagosome pathway, Antigen presentation* or *Metabolism of carbohydrates.*

Distinct biological mechanism related with DE genes were found in the lung compartments of BAL and parenchyma. Although most immune-related routes were found at the three time points studied, samples from animals obtained at 2 weeks after immunization or just after the third boost (at 14 weeks), displayed a more resembling profile in BAL than in lung parenchyma.

### Bacille Calmette–Guérin/Nano-FP1 Shows a Unique List of Differentially Expressed Genes at 2 Weeks

The large number of DE genes obtained in the BCG/Nano-FP1 group hindered a more detailed analysis of single genes involved in the Nano-FP1 boosting protective effect at short-term. Our next approach involved the filtering of the genes differentially expressed in BCG/Nano-FP1 at 2 weeks to obtain a reduced list of possible candidate biomarkers of *Mtb* enhanced protection.

**Figure 7:**
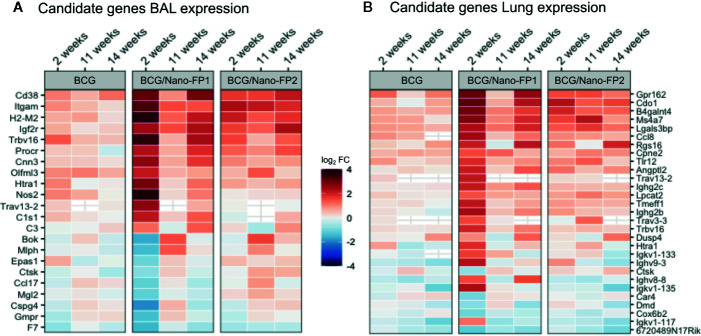
Candidate genes in BAL and Lung in the BCG/Nano-FP1 group at 2 weeks. After filtering, a list of 22 and 29 candidate genes obtained in BAL **(A)** and lung **(B)**, respectively, were selected by the differential expression analysis in the BCG/Nano-FP1 group (in terms of log2 fold Change), compared to control mice. Naive colored in red (upregulated) or blue (downregulated) among vaccination groups: BCG (received s.c BCG 12 weeks earlier); BCG/Nano-FP1 and BCG/Nano-FP2 (received sc. BCG 12^th^ weeks earlier followed by two (analyzed at weeks 2 and 11) or three (analyzed at week 14) intranasal administrations of the Nano-FP1 or Nano-FP2 vaccines, respectively).

BCG/Nano-FP1 group was pairwise compared by differential expression analysis to the other vaccination groups (Naive, BCG and BCG/Nano-FP2 for every time-point (2, 11, or 14 weeks). Then, three characteristic lists of genes of BCG/Nano-FP1 for 2, 11, and 14 weeks were obtained by the intersection of the differentially expressed (DE) genes set in mentioned pairwise comparison, i.e. genes differentiating BCG/Nano-FP1 from every other group. From the new sets of genes in each time point, we selected the relative complement of BCG/Nano-FP1 at 2 weeks’ genes. Thus, we obtained a list of candidate genes (**22 in BAL and 29 in lung)** (**Figure 7**, **Table 1**) that were exclusively DE in the BCG/Nano-FP1 group at 2 weeks, at its highest point of *Mtb* protection. The filtering strategy scheme is described in **Supplementary Figures S4E, F**.

**Table 1:** List of candidate genes in bronchoalvelolar lavage and lung cells, differentially expressed in the BCG/Nano-FP1 group at 2 weeks, compared to the other groups (naïve, BCG, BCG/Nano-FP2).

Bronchoalveolar lavage			
*Gen symbol*	*Ensemble gene ID*	*Protein codifying*	*Log_2_ fold Change*
**Nos2**	ENSMUSG00000020826	Nitric oxide synthase, inducible	3.72
**H2-M2**	ENSMUSG00000016283	Histocompatibility 2, M region locus 2	3.68
**Trbv16**	ENSMUSG00000076473	T cell receptor beta, variable 16	3.54
**Itgam**	ENSMUSG00000030786	Integrin alpha-M	3.29
**Cd38**	ENSMUSG00000029084	ADP-ribosyl cyclase/cyclic ADP-ribose hydrolase 1	3.10
**Htra1**	ENSMUSG00000006205	Serine protease HTRA1	2.86
**Trav13-2**	ENSMUSG00000076846	T cell receptor alpha variable 13-2	2.61
**Cnn3**	ENSMUSG00000053931	Calponin-3	2.59
**Igf2r**	ENSMUSG00000023830	Cation-independent mannose-6-phosphate receptor	2.46
**Procr**	ENSMUSG00000027611	Endothelial protein C receptor	2.25
**C1s1**	ENSMUSG00000038521	Complement component 1, s subcomponent 1	2.14
**Olfml3**	ENSMUSG00000027848	Olfactomedin-like protein 3	2.07
**C3**	ENSMUSG00000024164	Complement C3	1.14
**Ctsk**	ENSMUSG00000028111	Cathepsin K	−0.86
**Epas1**	ENSMUSG00000024140	Endothelial PAS domain-containing protein 1	−1.05
**Mgl2**	ENSMUSG00000040950	Macrophage galactose N-acetyl-galactosamine-specific lectin 2	−1.14
**Ccl17**	ENSMUSG00000031780	C-C motif chemokine 17	−1.14
**Gmpr**	ENSMUSG00000000253	GMP reductase 1	−1.43
**Mlph**	ENSMUSG00000026303	Melanophilin	−1.44
**F7**	ENSMUSG00000031443	Coagulation factor VII	−1.55
**Bok**	ENSMUSG00000026278	Bcl-2-related ovarian killer protein	−1.66
**Cspg4**	ENSMUSG00000032911	Chondroitin sulfate proteoglycan 4	−1.91
**Lung parenchyma**			
***Gen symbol***	*Ensemble gene ID*	*Protein codifying*	*Log_2_ fold Change*
**Cdo1**	ENSMUSG00000033022	Cysteine dioxygenase type 1	3.19
**Gpr162**	ENSMUSG00000038390	Probable G-protein coupled receptor 162	3.03
**B4galnt4**	ENSMUSG00000055629	N-acetyl-beta-glucosaminyl-glycoprotein 4-beta-N-acetylgalactosaminyltransferase 1	2.92
**Ms4a7**	ENSMUSG00000024672	Membrane-spanning 4-domains, subfamily A, member 7	2.73
**Ccl8**	ENSMUSG00000009185	C-C motif chemokine 8	2.41
**Ighg2b**	ENSMUSG00000076613	Immunoglobulin heavy constant gamma 2B	2.37
**Igkv1-135**	ENSMUSG00000096336	Immunoglobulin kappa variable 1-135	2.32
**Lgals3bp**	ENSMUSG00000033880	Galectin-3-binding protein	2.29
**Ighv9-3**	ENSMUSG00000096459	Immunoglobulin heavy variable V9-3	2.24
**Tmeff1**	ENSMUSG00000028347	Tomoregulin-1	2.12
**Trav13-2**	ENSMUSG00000076846	T cell receptor alpha variable 13-2	2.10
**Ighg2c**	ENSMUSG00000076612	Immunoglobulin heavy constant gamma 2C	2.08
**Rgs16**	ENSMUSG00000026475	Regulator of G-protein signaling 16	2.03
**Angptl2**	ENSMUSG00000004105	Angiopoietin-related protein 2	2.01
**Trbv16**	ENSMUSG00000076473	T cell receptor beta, variable 16	1.94
**Tlr12**	ENSMUSG00000062545	Toll-like receptor 12	1.84
**Htra1**	ENSMUSG00000006205	Serine protease HTRA1	1.81
**Igkv1-133**	ENSMUSG00000094491	Immunoglobulin kappa variable 1-133	1.58
**Trav3-3**	ENSMUSG00000094828	T cell receptor alpha variable 3-3	1.52
**Ighv8-8**	ENSMUSG00000104452	Immunoglobulin heavy variable 8-8	1.49
**Cpne2**	ENSMUSG00000034361	Copine-2	1.48
**Lpcat2**	ENSMUSG00000033192	Lysophosphatidylcholine acyltransferase 2	1.38
**Igkv1-117**	ENSMUSG00000094335	Immunoglobulin kappa variable 1-117	1.09
**Dusp4**	ENSMUSG00000031530	Dual specificity protein phosphatase 4	0.90
**Ctsk**	ENSMUSG00000028111	Cathepsin K	−0.76
**Dmd**	ENSMUSG00000045103	Dystrophin	−0.85
**Car4**	ENSMUSG00000000805	Carbonic anhydrase 4	−1.00
**Cox6b2**	ENSMUSG00000051811	Cytochrome c oxidase subunit 6B2	−1.06
**6720489N17Rik**	ENSMUSG00000072066	KRAB domain-containing protein	−1.25

Most of the candidate genes were related to immune defence mechanisms. They include: upregulated **T cell receptor** (*Trbv16, Trav13-2, Trav3-3*) and genes coding for **immunoglobulin chains** (*Ighg2b, Igkv1-135, Ighv9-3, Igkv1-133, Igkv1-117, Ighg2c*); **cytokine-related genes** (*Ccl17 downregulated, Ccl8 upregulated*); a couple of genes related to **macrophages** including upregulated Angptl2 and downregulated Mgl2; upregulated genes coding for **Complement cascade** *(C1s1* and *C3)*, upregulated *H2-M2* as part of **MHC class Ib**, increased expression of Nitric oxide synthase ***Nos2*** and Toll-like receptor ***Tlr12*,** genes of **extracellular matrix related proteins**, or downregulated **cathepsin K** gene (Ctsk). Three DE genes were shared in BAL and Lung, two of them upregulated: **Htra 1** (serine protease HTRA1) and **T cell receptor alpha variable** (13-2), and one downregulated, **Cathepsin K**.

### Validation of Differential Gene Expression by RT-qPCR

To confirm the reliability of the gene expression results by RNA-Seq, we validated by RT-qPCR a total of 17 DE genes from the list of top-expressed candidate genes at 2 weeks: Nos2, H2M2, Trbv16, Itgam, Cd38, Htra1, Ccl17, Gmpr, Mlph, F7, Bok, and Cspg4 in BAL and Cdo1, Ms4a7, Trbv16, Htra1, Car4, Cox6b2, and Ctsk in lung parenchyma samples. Due to low RNA amount, we analyzed BCG/Nano-FP1 vs BCG/Nano-FP2 groups in BAL. To increase robustness, we used biological replicates from independent mice, and we compared log2 fold changes obtained by both methods.

As it is shown in **Table 2**, we found similar expression levels in all comparisons, validating the results obtained by RNA-Seq for these genes.

**Table 2:** Comparison of the expression levels of 17 differentially expressed genes by RNA-Seq and RT-qPCR.

Bronchoalveolar lavage								
*Gen symbol*			BCG/Nano-FP1 vs Naive 2 weeks					
**			*RNA-Seq Log_2_ fold Change*			*RT-qPCR mean Log2 fold Change ± SEM*		
**Nos2**			3.66			4.24 ± 0.65		
**H2-M2**			1.64			2.00 ± 0.33		
**Trbv16**			1.97			2.21 ± 0.24		
**Itgam**			0.96			1.74 ± 0.33		
**Cd38**			1.42			1.24 ± 0.39		
**Htra1**			2.14			3.17 ± 0.48		
**Ccl17**			−1.07			−0.71 ± 0.37		
**Gmpr**			−0.98			−1.08 ± 0.19		
**Mlph**			−1.37			−1.49 ± 0.47		
**F7**			−0.94			−0.72 ± 0.26		
**Bok**			−1.51			−1.56 ± 0.36		
**Cspg4**			−1.59			−1.95 ± 0.080		
**Lung parenchyma**								
***Gen symbol***	**BCG/Nano-FP1 vs Naive** **2 weeks**			**BCG/Nano-FP1 vs BCG** **2 weeks**			**BCG/Nano-FP1 vs BCG/Nano-FP2** **2 weeks**	
	***RNA-Seq Log_2_ fold Change***	***RNA-Seq Log_2_ fold Change ± SEM***		***RNA-Seq Log_2_ fold Change***	***RNA-Seq Log_2_ fold Change ± SEM***		***RNA-Seq Log_2_ fold Change***	***RNA-Seq Log_2_ fold Change ± SEM***
**Cdo1**	3.19	3.67 ± 0.25		2.68	3.45 ± 0.25		0.95	0.56 ± 0.25
**Ms4a7**	2.73	3.48 ± 0.25		1.97	2.98 ± 0.25		1.12	0.97 ± 0.25
**Trbv16**	1.94	2.52 ± 0.36		1.73	2.25 ± 0.36		1.61	2.06 ± 0.36
**Htra1**	1.81	1.92 ± 0.51		1.10	1.32 ± 0.51		1.69	1.89 ± 0.51
**Ctsk**	−0.76	−1.03 ± 0.22		−0.60	−0.34 ± 0.22		−1.04	−1.17 ± 0.22
**Car4**	−1.00	−1.35 ± 0.35		−0.74	−0.87 ± 0.35		−0.99	−1.04 ± 0.35
**Cox6b2**	−1.06	−1.24 ± 0.16		−1.00	−0.80 ± 0.16		−0.90	−0.60 ± 0.16

## Discussion

Several novel TB vaccine candidates are currently in Phase II/III clinical trials (1) but despite major advances in TB research and vaccine development, BCG remains the only licensed TB vaccine to be used in humans. Development of new and more effective TB vaccines is a global priority. However, the lack of validated immune correlates of protection is a major hurdle in the development of novel vaccines.

Respiratory mucosal vaccination is proposed to be the most effective strategy, mimicking the natural route of *Mtb* infection in the lungs and thus inducing a better local immune response (16, 18, 30). Further, intranasal mucosal route has garnered attention due to its non-invasiveness and accessibility, allowing easy repeated vaccination if necessary (12–15, 17). It has also been observed in novel vaccination strategies that a better outcome could be achieved by the combination of systemic BCG-given immunity reinforced by mucosal boosting. Although BCG has moderate and heterogeneous efficacy on TB, it has a proven role in lowering infant mortality (31). Therefore, BCG vaccination is still a common practice in many countries which is advantageous as an effective priming for this type of vaccination strategy.

In the current study, we aimed to analyze the factors contributing to the duration of immunological memory after vaccination and also to make a correlation between immunological responses and protection. For this, we have used two candidate vaccines namely Nano-FP1 and Nano-FP2 together with BCG and extended the time of infection after vaccination from the short time (2 weeks) to 7 weeks and later. We consider of major importance when assessing novel vaccines, not only to determine the responsiveness of the immune system, but the durability of protection. We investigated the effect of these nanovaccines at different time-points and identified the protection-related immunological signature and genes mobilized in response to the vaccine.

Our results confirm previous data showing the efficacy of the BCG/Nano-FP1 vaccine in animals infected short time (2 weeks) after a second boost with the nanovaccine, by lowering the number of CFUs and triggering several changes in immune cell populations (26). However, both the protection and the phenotype profile were partially lost after 7 weeks and beyond. We found several changes in immune populations at 2 weeks after the 2^nd^ Nano-FP1 boost, with an increased proportion of both CD4^+^ and CD8^+^ T cells, higher percentages of RM CD4^+^ and CD8^+^ T cells, and CD4^+^ cells synthetizing cytokines (IFNγ, TNFα, and/or IL-2), either monofunctionally or polyfunctionally. A partial modulation of the immune profile was also observed in mice receiving the Nano-FP2, which could be explained as a boost of the BCG immunization, by the intranasal administration.

At short term after immunization, our results are in accordance with the central dogma of TB immunity, where CD4^+^ Th1 cells represent the major T cell subset that participates in the immune response to *Mtb* (4–6, 32, 33). Also, their secreted proinflammatory cytokines (IFNγ, TNFα, and IL-2) are essential for control of bacterial growth in both animal models and humans, by the activation of macrophages (4–6, 32, 33). More recently IL-17+ (34) and polyfunctional CD4^+^ T cells, able to produce multiple cytokines, were also associated with protection (7–9, 35–37). Resident memory (RM) vaccine-induced T cells have been postulated as a new desired target due to their lung-homing capacity, with promising results (17, 25), including Hart et al. (26). Our work confirms previous results, with elevated percentages of CD4^+^ and CD8^+^ RM cells in BCG/Nano-FP1 group. However, this profile was lost at later time points.

Since the phenotypic profile observed at 2 weeks was lost from 7 weeks on, we wondered whether the administration of a third intranasal nanovaccine boost could recover the immune phenotype. In fact, most of the mobilized CD4 population observed at week 2, including total and RM CD4^+^ T cells, reached similar levels to short-term and a clear increase was observed in the number of total cytokine-secreting cells compared to the 2 weeks profile, especially in the number of TNFα+ and IFNγ+ TNFα+ producing CD4^+^ cells. On the contrary, the levels of total and RM CD8^+^ cells did not reach the same profile found at 2 weeks. One limitation of this study is the absence of intravascular staining to discriminate lung parenchyma immune cells from those from blood, a technique that has gained attention in the last decade (38–42). The analysis of the T cell homing markers indicates an important role of the tissue-resident cells in the response to the BCG/Nano-FP1 vaccine, although further studies could confirm the origin of those lymphocytes in lung parenchyma. Moreover, a fully understanding of the immune cell dynamics and migration might reveals new insights into the effects induced by the nanovaccine.

In summary, and opposed to the 2-week scenario, the recovery of the immunological profile, and even the cytokine polyfunctionality induced by the Nano-FP1 vaccine, was not enough to exert the expected increased protective effect against the *Mtb*, being even inferior to the levels reached at 2 weeks. These results suggest that Nano-FP1 repeated boosting set positive lung conditions at short term (2 weeks) that lowered bacterial load, but ultimately did not trigger an effective memory response at long term. Furthermore, our findings call for increased efforts when characterizing novel vaccines, by assessing both short and long-term outcomes.

In order to obtain more information, we were encouraged to analyze which genes were mobilized by BCG/Nano-FP1. We first investigated the type of response induced as soon as 24 h after the intranasal boost. We reasoned that analysis at this very short time would reveal the main innate effect of the vaccine and delivery system before the development of the adaptive immunity and in consequence, assess the capacity of the vaccine to create an appropriate environment in the lungs. This analysis asserted how one single Nano-FP1 boost prompted immediate and substantial transcriptomic changes. Nevertheless, comparative transcriptomic profiling among primed and non-primed Nano-FP1 provides compelling evidence of the relevance of BCG priming for the outcome. Furthermore, BCG/Nano-FP1 triggered mobilization of genes at 2 and 14 weeks in both BAL and lung parenchyma, but the response decreased at 11 weeks, suggesting its transient nature.

The main enriched pathways identified in the nanovaccine groups at 2 and 14 weeks in BAL were associated with many immune-related pathways such as **cytokine signaling, TCR and BCR signaling or complement cascade.** DAP12 signaling was also observed, involving **DAP12-mediated** activation signals in NK cells, granulocytes, monocytes/macrophages, and DCs (43, 44), although a published study associated activation of DAP12 in APCs with the delay in Th1 immunity in TB (45).

Moreover, we found pathways upregulated exclusively at 2 weeks, such as **IFNγ signaling**, cytokine known to be essential in TB defence (4–6) and **antigen presentation by class II MHC**, supporting a potentially significant role of CD4 T cells. Furthermore, **Fc gamma receptor dependent phagocytosis** pathway was upregulated, suggesting the contribution of that mechanism in elimination of invading pathogens mediated by immunoglobulins. Lastly, extracellular matrix organization and integrin interactions were also exclusively upregulated at 2 weeks. A role for the **extracellular matrix** has also been reported in regulating the host-pathogen interaction in TB (46).

In lung parenchyma, we found more differences among the transcriptome profiles in nanovaccine groups at 2 and 14 weeks. Around 1300 DE genes appeared unique to the BCG/Nano-FP1 group at 14 weeks, compared to the other time-points, which was ten times more than those DE genes found in BAL (**Supplementary Figure S4**). Regarding pathway enrichment analysis, we found that at 2 weeks, samples displayed a higher number of pathways with upregulated DE genes, most of them coinciding with immune-related pathways found in BAL. These intriguing differences among lung sections could reflect specific mechanisms involved in the enhancement (2 weeks) or not (14 weeks) in TB protection by BCG/Nano-FP1. Moreover, it should also be considered that the effect of a single (14 weeks) or double (2 weeks) boost with the nanovaccine could be the responsible for those differences, with a single dose triggering more easily changes in BAL than in the lung parenchyma. One of the most significant pathways shared among 2- and 14-week samples was surprisingly the **cholesterol biosynthesis** pathway. Although directly not immune-related, cholesterol has been reported as necessary for *Mtb* metabolism (47–49).

However, some new signatures, uniquely related to the 2 weeks post-immunization, were found in lung parenchyma, such as ROS and RNS production in phagocytes, Scavenger receptors, endosomal TLR, signaling through P2Y receptors, Sphingolipid metabolism or regulation of Insulin-like Growth Factor. ROS and RNS production have been well known as key macrophage bactericidal responses to Mtb, although the mycobacteria have developed sophisticated mechanisms to avoid them (50). Toll-like receptors play a key role in both innate immune responses and the initiation of adaptive immunity to Mtb, leading to phagocytic activation. TLRs including 2, 4, 9, and 8 are known to play critical roles in recognition of Mtb (51, 52). Regarding P2Y receptors, they activate intracellular signaling cascades to regulate a variety of cellular processes, including their use by macrophages to combat Mtb (53). Also, scavenger receptor pathways could participate in the Mtb infection outcome. Some scavenger receptors have been directly related to host and pathogenic cholesterol uptake, and to mycobacterial recognition by macrophages (54, 55).

No major changes in cell populations or transcriptome were found in the BCG-vaccinated groups, although its protective effect was evident. We cannot provide a better explanation than to propose different mechanisms of protection are involved and that the nanovaccine is promoting a pathway dependent of BCG, but BCG itself is inducing additional different pathways that are essential for protection.

As a large list of genes identified related with the transcriptional signature of nanovaccine-vaccinated mice, the refined analysis identified unique transcriptomic signature by BCG/Nano-FP1 at 2 weeks, obtaining a list of 22 genes in BAL and 29 in lung parenchyma (**Table 1**). Several **T cell receptor** (*Trbv16, Trav13-2, Trav3-3*), **immunoglobulin chains-coding genes** (*Ighg2b, Igkv1-135, Ighv9-3, Igkv1-133, Igkv1-117, Ighg2c*) and cytokine-related genes (*Ccl17, Ccl8*) were upregulated.

A couple of genes related to **macrophages** were identified, including the upregulated *Angptl2*, a secreted factor able to attract and activate macrophages, and *Mgl2* Macrophage galactose N-acetyl-galactosamine-specific lectin 2, which was downregulated. ***Nos2*** was also upregulated in BCG/Nano-FP1 group. *Nos2* enzyme produces nitric oxide, which mediates bactericidal actions in macrophages, and has been described important in TB host defence. Among mechanism involved in triggering of the immune response, we found upregulated *C1s1* and *C3* coding for **Complement proteins**, and *H2-M2* as part of **MHC class Ib**. Another key type of receptors that initiate immune responses to pathogens, TLRs, were represented with the augmented expression of ***Tlr12***. Although little is known about this protein, recently a role of TLR12 was described in activating macrophages by *Mtb* antigens (56).

Lastly, we observed expression changes in some of the **extracellular matrix related proteins**, which may play an important role in extracellular matrix degradation in TB disease: serine protease HTRA1 (*Htra1*) was upregulated, and **cathepsin K** gene (*Ctsk*) was downregulated in both BAL and lung. The collagenase has been reported previously to contribute to TB cavitation (57).

In keeping with previous observations, we believe this list of genes could be a valuable source of potential biomarkers correlated with protection, as they are driving local lung responses to *Mtb* infection.

Validation of RNA-Seq results was performed by quantitative RT-qPCR analysis of some of the top-fold change DE genes of BCG/Nano-FP1 group at 2 weeks. 17 genes were studied and both techniques showed similar gene expression levels (**Table 2**), supporting the reliability of our overall findings on the candidate genes and biological pathways, that might play a role in TB protection.

Some of the validated genes are related to key defense pathways. Upregulated *H2-M2* and *Trbv16* genes might participate in increasing antigen presentation and recognition (as they codify for proteins of MHC-I and TCR, respectively), while overexpressed *Cd38* and *Itgam* might reflect changes in leucocyte populations, activation or migration (58, 59). As mentioned, upregulated *Nos2* has long been known as a key element in ROS anti-mycobacterial response (60). Decreased expression of *Ccl17* might favor the deviation of the immune population toward a Th1 response (61).

Our results also suggest the importance of other mechanisms in TB infection, for instance, upregulated membrane compound *Ms4a7* was previously reported as altered in TB studies (62), being involved in signal cell transduction. The downregulated gene *Bok* is one of the less studied members of the BCL-2 family, and no critical function has been assigned yet, although they work as critical regulators of apoptosis (63). Our findings also indicate an important role for the extracellular matrix organization, a pathway upregulated in the enrichment analysis of BCG/Nano-FP1 BAL at 2 weeks, with three validated genes possibly participating in the process: *Htra1* (64), *Ctsk* (57) and *Cspg4* (65).

For other validated genes, although presenting major fold change differences, we could not provide feasible hypotheses for their potential role in TB protection or immune system functioning, as is the case of *Cdo1* (Cysteine Dioxygenase Type 1, upregulated), *F7* (Coagulation Factor VII, downregulated), *Gmpr* (Guanosine monophosphate reductase 1, downregulated) or *Mlph* (Melanophilin, downregulated). We encourage future studies to target the wide range of altered pathways and biologically appealing DE genes found in this work.

Results obtained from both phenotypic and gene expression analysis suggest that the protective state achieved at short term after the nanovaccine boosting might be a combination of both lymphocytic and innate immune fractions. However, the precise role in bacterial control of the different leukocyte populations is poorly understood. While the participation of T cells, especially tissue-resident T cells, is known to be essential in TB protection, several research lines have moved toward the study of the innate fraction, particularly in light of the critical importance of early bacterial control on the outcome of the infection (66–69). We detected changes on gene expression as soon as 24 h after the first nanovaccine boosting, pointing toward the action of innate immune cells. Whether these changes are due to long-term epigenetic reprograming (“trained immunity”) or not, remains to be cleared. Further work involving depletion of specific immune populations and adoptive transfer experiments could help to establish the contribution of each population to the protective outcome. Analysis of the post-translational modifications driven by the nanovaccine on the innate cell population, will be considered in future investigations.

In summary, we show here that immune responses and long-term protection are not well correlated. Protection against *Mtb* infection promoted by the BCG/Nano-FP1 vaccine candidate and possibly other vaccines, is of short duration and may require a number of frequent boosts to keep the protective ability of the vaccine. A partial recovery of the immunological profile and protection could be improved, but the maintenance of this long-term protection requires further investigation. Several mechanisms have been identified that could explain the enhanced protection of the vaccine at 2 weeks after the intranasal boost. We provide a list of genes that are exclusively up or downregulated during the “protection window,” which could provide new information for TB vaccine design. Further, we consider that TB protection is established by a complex cooperation of several immune populations, including CD4^+^, CD8^+^ and innate cells, driving specific and a tight gene regulation. Thus, this study proposes a list of genes and distinct molecular pathways to be considered as important when directing future TB vaccine efforts and research.

## Data Availability Statement

The datasets presented in this study can be found in online repositories. The name of the repository and accession number can be found here: https://www.ebi.ac.uk/arrayexpress/, E-MTAB-9449.

## Ethics Statement

The experimental animal procedures were approved by Vigo University Committee and authorized by the competent authority (Xunta de Galicia, Conselleria do Medio Rural, Pontevedra, Spain) and by Stockholm North Ethical Committe on animal experiments.

## Author Contributions

AM-P performed RNA purification, analyzed the experimental data, and wrote the paper with input from all other authors. AM-P, AI, OE, and CMF performed most animal procedures and sample processing. AC helped with the animal vaccinations. ET, AGC, and CMF provided counseling. LA, A-LS and MLG contributed to the early response experiments procedures and sample processing. MS and RR contributed to the counseling and provided nanovaccines and peptides. AG-F conceived the study, made the drafting and the critical revision of the manuscript. All authors contributed to the article and approved the submitted version.

## Funding

This work was supported by the projects EU Horizon2020: “Eliciting Mucosal Immunity in Tuberculosis”(EMI-TB) project (Grant Number 643558); Portugal National funds, through the Foundation for Science and Technology (FCT) - project PTDC/SAU-INF/28463/2017, UIDB/50026/2020, and UIDP/50026/2020; NORTE-01-0145-FEDER-000013 and NORTE-01-0145-FEDER-000023, supported by Norte Portugal Regional Operational Programme (NORTE 2020), under the PORTUGAL 2020 Partnership Agreement, through the European Regional Development Fund (ERDF). This work also received financial support from the Xunta de Galicia (Grupo de Referencia Competitiva-[ED431C 2016/041]) and Centro singular de investigación de Galicia and the European Regional Development Fund (ERDF)-[ED431G2019/06]. AP acknowledges a fellowship from Xunta de Galicia Programa de axudas á etapa predoutoral (Consellería de Cultura, Educación e Ordenación Universitaria) (ED481A-2018/230). ET was supported by the FCT investigator grant IF/01390/2014 and CMF through the FCT PhD fellowship PD/BD/137447/2018.

## Conflict of Interest

MS is the Founding Director of LIONEX Diagnostics & Therapeutics GmbH, based in Braunschweig. AG-F is a co-promoter of the spin-off company NanoImmunoTech, based in Vigo, Spain.

The remaining authors declare that the research was conducted in the absence of any commercial or financial relationships that could be construed as a potential conflict of interest.

The reviewer NT declared a past co-authorship with several authors, A-LS and LA, to the handling editor.
