# Supplementary material for: Changes in the Immune Phenotype and Gene Expression Profile Driven by a Novel Tuberculosis Nanovaccine: Short and Long-Term Post-immunization
Source: Front Immunol. 2021 Jan 28;11:589863. doi: 10.3389/fimmu.2020.589863 (PMC7876410; doi:10.3389/fimmu.2020.589863)
Supplement: Supplementary file 1 [file DataSheet_1.docx]

# SUPPLEMENTARY FIGURE LEGENDS

**Figure S.1.** **Flow Cytometry gating strategy** for (A) immunophenotype analysis of general population and (B) lymphocytic immunophenotype and intracellular cytokine staining (ICCS) analysis in lung.

**Figure S2. Kinetics of immune cell populations in lung.** **(A)** Lung immune cell populations: CD4 and CD8 T cells, B cells, neutrophils, inflammatory monocytes, resident monocytes, eosinophils, Natural killer (NK) cells, alveolar macrophages, exudate macrophages, interstitial macrophages, CD11b^+^ and CD11b^-^ CD103^+^ dendritic cells analyzed by flow cytometry at 2, 7, 11 weeks after the 2^nd^ nanovaccine boost and at 14 weeks (3 weeks after the 3^rd^ boost). Data represent percentages of each cell population referred to the total of immune CD45 positive cells. Kruskal–Wallis test and Dunn's multiple comparisons test were used for statistical analysis. *p<0.05.

**Figure S3. Analysis of CD8**^+^ **cytokine polyfunctionality in lung.** Mice were vaccinated as described for Figure 1. Production of either INFɣ, TNFα, IL-2 and IL-17 or their combinations were analyzed at 2, 7 and 11 weeks after two intranasal nanovaccine boosts or at 14 weeks, three weeks after a third intranasal nanovaccine boost. **(A**) Data represent percentages of cytokine-producing activated CD8^+^ T cells at 2 or 14 weeks that produced 1 cytokine (INFɣ, TNFα, IL-2 or IL-17) or the combination of 2, 3 or 4 of those cytokines, referred to the total of CD4 T cells. **(B, C)** Data represent percentages of cytokine-producing activated CD8^+^ T cells at 2 **(B)** or 14 weeks **(C)** referred to the total of CD8^+^ T cells. Kruskal–Wallis test and Dunn's multiple comparisons test were used for statistical analysis. *p<0.05.

**Figure S.4.** **Transcriptomic changes at short, long term and re-immunization after vaccination.** Volcano plot representation of the differential expression analysis between BCG and BCG/Nano-FP2 groups compared to control Naive group in bronchoalveolar lavage **(A)** and lung parenchyma **(B)**. 3 samples of individual or pooled mice were analyzed per group. The differential expression analysis was made using DESeq2 R package, comparing all annotations of the reference genome (52636 annotations). Significantly DE genes (p adjusted < 0,05 & fold change ≥ 1.5) colored in red (upregulated) or blue (downregulated). Names of top-5 p-adjusted DE genes are plotted. Venn Diagram representing BCG/Nano-FP1 vs control DE genes shared among studied time-points in BAL **(C)** and lung parenchyma **(D)**. Scheme of filtering strategy used to obtain candidate DE genes uniquely expressed in BAL at 2 weeks in the BCG/Nano-FP1 group. **(E)** and lung parenchyma **(F)**. First, BCG/Nano-FP1 group was compared against Naïve, BCG or BCG/Nano-FP2 at each time-point (2, 11 and 14 weeks). Then, DE genes appearing in the BCG/Nano-FP1group analyzed at 2 weeks, but not in BCG/Nano-FP1 at 11 or 14 weeks, were selected as potential gene candidates.

# SUPPLEMENTARY TABLES

**Table S.1**. List of antibodies used in flow cytometry

| Name | Provider | Reference |
| --- | --- | --- |
| Brilliant Violet 510™ anti-mouse CD45 | Biolegend | 103138 |
| Brilliant Violet 605™ anti-mouse CD3 | Biolegend | 100351 |
| APC/Cy7 anti-mouse CD4 | Biolegend | 100414 |
| Brilliant Violet 711™ anti-mouse CD8a | Biolegend | 100759 |
| PE-Cy7 anti-mouse/human CD11b | Biolegend | 101216 |
| Brilliant Violet 605™ anti-mouse CD11c | Biolegend | 117334 |
| Brilliant Violet 650™ anti-mouse CD19 | Biolegend | 115541 |
| Pacific Blue™ anti-mouse CD24 | Biolegend | 101820 |
| PerCP/Cy5.5 anti-mouse/human CD44 | Biolegend | 103032 |
| PE/Cy7 anti-mouse CD62L | Biolegend | 104418 |
| Brilliant Violet 711™ anti-mouse CD64 (FcγRI) | Biolegend | 139311 |
| PE anti-mouse CD69 (H1.2F3) | BD Pharmingen | 553237 |
| FITC anti-mouse CD127 (IL-7Rα) | Biolegend | 135007 |
| APC anti-mouse CD103 | Biolegend | 121410 |
| IFNγ -PE-Cy7 anti-mouse | Biolegend | 505826 |
| APC anti-mouse IL-17A | Biolegend | 506916 |
| PE anti-mouse IL-2 | eBiosciences | 12-7021-82 |
| Ly6C- PerCP-Cy5.5 anti-mouse | Biolegend | 128012 |
| Brilliant Violet 785™ anti-mouse Ly-6G | Biolegend | 127645 |
| FITC anti-mouse I-A/I-E | Biolegend | 107606 |
| PE Rat-Mouse Siglec-F | BD Pharmingen | 552126 |
| FITC anti-mouse TNF-α | Biolegend | 506304 |

**Table S.2.** Reactome Pathway Enrichment results obtained from 24h analysis

| Cluster | ID | Description | p.adjust | geneID | Count |
| --- | --- | --- | --- | --- | --- |
| BCG/PBS-24H up | R-MMU-2168880 | Scavenging of heme from plasma | 0.00124677264924068 | Apol10b/Apol9a/Apol9b | 3 |
| BCG/PBS-24H up | R-MMU-2173782 | Binding and Uptake of Ligands by Scavenger Receptors | 0.00247850260440607 | Apol10b/Apol9a/Apol9b | 3 |
| BCG/PBS-24H up | R-MMU-1236975 | Antigen processing-Cross presentation | 0.0218293765174722 | H2-Q7/H2-Q6/Psmb9 | 3 |
| BCG/PBS-24H up | R-MMU-1236977 | Endosomal/Vacuolar pathway | 0.0218293765174722 | H2-Q7/H2-Q6 | 2 |
| BCG/PBS-24H up | R-MMU-8854691 | Interleukin-20 family signaling | 0.0219861111957726 | Stat1/Stat2 | 2 |
| BCG/PBS-24H up | R-MMU-912694 | Regulation of IFNA signaling | 0.0219861111957726 | Stat1/Stat2 | 2 |
| BCG/PBS-24H up | R-MMU-8957275 | Post-translational protein phosphorylation | 0.0219861111957726 | Apol10b/Apol9a/Apol9b | 3 |
| BCG/PBS-24H up | R-MMU-1236974 | ER-Phagosome pathway | 0.0219861111957726 | H2-Q7/H2-Q6 | 2 |
| BCG/PBS-24H up | R-MMU-909733 | Interferon alpha/beta signaling | 0.0219861111957726 | Stat1/Stat2 | 2 |
| BCG/PBS-24H up | R-MMU-381426 | Regulation of Insulin-like Growth Factor (IGF) transport and uptake by Insulin-like Growth Factor Binding Proteins (IGFBPs) | 0.0258930328609292 | Apol10b/Apol9a/Apol9b | 3 |
| BCG/PBS-24H up | R-MMU-983170 | Antigen Presentation: Folding, assembly and peptide loading of class I MHC | 0.0258930328609292 | H2-Q7/H2-Q6 | 2 |
| BCG/PBS-24H down | R-MMU-163210 | Formation of ATP by chemiosmotic coupling | 0.00526662277814305 | ATP8 | 1 |
| BCG/PBS-24H down | R-MMU-8949613 | Cristae formation | 0.00526662277814305 | ATP8 | 1 |
| BCG/PBS-24H down | R-MMU-1592230 | Mitochondrial biogenesis | 0.00526662277814371 | ATP8 | 1 |
| BCG/PBS-24H down | R-MMU-163200 | Respiratory electron transport, ATP synthesis by chemiosmotic coupling, and heat production by uncoupling proteins. | 0.0136603028308097 | ATP8 | 1 |
| BCG/PBS-24H down | R-MMU-1428517 | The citric acid (TCA) cycle and respiratory electron transport | 0.0177748518762345 | ATP8 | 1 |
| BCG/PBS-24H down | R-MMU-1852241 | Organelle biogenesis and maintenance | 0.022822032038622 | ATP8 | 1 |
| PBS/Nano-FP1-24h up | R-MMU-380108 | Chemokine receptors bind chemokines | 3.37166402384814e-05 | Cxcl5/Cxcl3/Cxcl1/Cxcl2/Ccl19/Ccr5 | 6 |
| PBS/Nano-FP1-24h up | R-MMU-2173782 | Binding and Uptake of Ligands by Scavenger Receptors | 7.42260982456635e-05 | Apol7a/Msr1/Hpx/Apol9a/Apol9b | 5 |
| PBS/Nano-FP1-24h up | R-MMU-1169408 | ISG15 antiviral mechanism | 0.000178013839644521 | Mx2/Eif2ak2/Usp18/Isg15 | 4 |
| PBS/Nano-FP1-24h up | R-MMU-2168880 | Scavenging of heme from plasma | 0.000178013839644521 | Apol7a/Hpx/Apol9a/Apol9b | 4 |
| PBS/Nano-FP1-24h up | R-MMU-1169410 | Antiviral mechanism by IFN-stimulated genes | 0.00020177120578677 | Mx2/Eif2ak2/Usp18/Isg15 | 4 |
| PBS/Nano-FP1-24h up | R-MMU-913531 | Interferon Signaling | 0.000380643161680969 | Mx2/Eif2ak2/Usp18/Isg15/Stat2 | 5 |
| PBS/Nano-FP1-24h up | R-MMU-375276 | Peptide ligand-binding receptors | 0.00776912909452443 | Cxcl5/Cxcl3/Cxcl1/Cxcl2/Ccl19/Ccr5 | 6 |
| PBS/Nano-FP1-24h up | R-MMU-418594 | G alpha (i) signalling events | 0.0142358544788508 | Ccl4/Cxcl5/Cxcl3/Cxcl1/Hcar2/Cxcl2/Ccl19/Ccr5 | 8 |
| PBS/Nano-FP1-24h up | R-MMU-1280215 | Cytokine Signaling in Immune system | 0.016475196625745 | Mx2/Eif2ak2/Il1rn/Tnfrsf9/Usp18/Isg15/Stat2/Osm | 8 |
| PBS/Nano-FP1-24h up | R-MMU-373076 | Class A/1 (Rhodopsin-like receptors) | 0.0166403220688319 | Cxcl5/Cxcl3/Cxcl1/Hcar2/Cxcl2/Ccl19/Ccr5 | 7 |
| PBS/Nano-FP1-24h up | R-MMU-977068 | Termination of O-glycan biosynthesis | 0.0359733105049415 | Muc5b/Muc4 | 2 |
| PBS/Nano-FP1-24h up | R-MMU-8957275 | Post-translational protein phosphorylation | 0.0380517333253988 | Timp1/Apol7a/Apol9a/Apol9b | 4 |
| PBS/Nano-FP1-24h up | R-MMU-114608 | Platelet degranulation | 0.0495768552834094 | Timp1/Lgals3bp/Orm2/Serpina3m | 4 |
| PBS/Nano-FP1-24h up | R-MMU-381426 | Regulation of Insulin-like Growth Factor (IGF) transport and uptake by Insulin-like Growth Factor Binding Proteins (IGFBPs) | 0.0495768552834094 | Timp1/Apol7a/Apol9a/Apol9b | 4 |
| PBS/Nano-FP1-24h up | R-MMU-76005 | Response to elevated platelet cytosolic Ca2+ | 0.0495768552834094 | Timp1/Lgals3bp/Orm2/Serpina3m | 4 |
| PBS/Nano-FP1-24h up | R-MMU-500792 | GPCR ligand binding | 0.0499016048584948 | Cxcl5/Cxcl3/Cxcl1/Hcar2/Cxcl2/Ccl19/Ccr5 | 7 |
| PBS/Nano-FP1-24h up | R-MMU-912694 | Regulation of IFNA signaling | 0.0499016048584948 | Usp18/Stat2 | 2 |
| PBS/Nano-FP1-24h down | R-MMU-373076 | Class A/1 (Rhodopsin-like receptors) | 0.0162560946810082 | Ptger3/Oxtr | 2 |
| PBS/Nano-FP1-24h down | R-MMU-391903 | Eicosanoid ligand-binding receptors | 0.0162560946810082 | Ptger3 | 1 |
| PBS/Nano-FP1-24h down | R-MMU-500792 | GPCR ligand binding | 0.0162560946810082 | Ptger3/Oxtr | 2 |
| PBS/Nano-FP1-24h down | R-MMU-75876 | Synthesis of very long-chain fatty acyl-CoAs | 0.0162560946810082 | Elovl6 | 1 |
| PBS/Nano-FP1-24h down | R-MMU-75105 | Fatty acyl-CoA biosynthesis | 0.021248003394709 | Elovl6 | 1 |
| BCG/Nano-FP1-24h up | R-MMU-1280215 | Cytokine Signaling in Immune system | 8.12958523510991e-13 | Lck/Il12rb1/Irf9/Relb/Hck/Ptpn6/Il12b/Csf1/Il12rb2/Psme1/Cd4/Mx2/Eif2ak2/Psmb8/Tnf/Lta/Nfkb2/Il6/Casp1/Il18rap/Stat1/Il2ra/Il1rn/Ube2l6/Il1b/Il1a/Tnfsf8/Tnfrsf1b/Tnfrsf9/Tnfrsf4/Usp18/Il21r/Il2rg/Psmb10/Il10ra/Uba7/Crlf2/Isg15/Socs1/Stat2/Ddx58/Tnfrsf18/Tnfrsf14/Socs3/Ifng/Osm/Il2rb/Il18bp/Psme2b/Psme2/Psmb9 | 51 |
| BCG/Nano-FP1-24h up | R-MMU-389948 | PD-1 signaling | 1.37291622426555e-10 | Lck/Cd3g/Ptpn6/Cd274/Pdcd1lg2/Cd4/Cd3e/H2-Aa/H2-Eb1/H2-Eb2/H2-Ab1 | 11 |
| BCG/Nano-FP1-24h up | R-MMU-202433 | Generation of second messenger molecules | 8.21250811032709e-10 | Lck/Cd3g/Lcp2/Itk/Fyb/Cd4/Lat/Cd3e/H2-Aa/H2-Eb1/H2-Eb2/H2-Ab1 | 12 |
| BCG/Nano-FP1-24h up | R-MMU-380108 | Chemokine receptors bind chemokines | 2.2248948032024e-09 | Cxcl16/Ccl20/Xcl1/Cxcl5/Cxcl3/Cxcl1/Cxcl9/Cxcl10/Ccl5/Ccr3/Cxcr6/Cxcr3/Cx3cr1/Cxcl2/Ccl19/Ccr5 | 16 |
| BCG/Nano-FP1-24h up | R-MMU-198933 | Immunoregulatory interactions between a Lymphoid and a non-Lymphoid cell | 5.81726293760239e-09 | Itgb7/Cd3g/H2-M3/C3/Ifitm3/Siglec1/Klrk1/Crtam/Cd3e/H2-Q4/Slamf7/Cd300e/Cd8b1/Cd8a/Lair1/H2-Q7/B2m/H2-K1/H2-T23/H2-Q6/H2-D1 | 21 |
| BCG/Nano-FP1-24h up | R-MMU-202430 | Translocation of ZAP-70 to Immunological synapse | 1.08723904645798e-07 | Lck/Cd3g/Cd4/Cd3e/H2-Aa/H2-Eb1/H2-Eb2/H2-Ab1 | 8 |
| BCG/Nano-FP1-24h up | R-MMU-1236975 | Antigen processing-Cross presentation | 1.08723904645798e-07 | Fcgr1/H2-M3/Psme1/Tapbp/Psmb8/Tap2/Psmb10/H2-Q4/Tap1/H2-Q7/B2m/H2-K1/H2-T23/H2-Q6/H2-D1/Psme2b/Psme2/Psmb9 | 18 |
| BCG/Nano-FP1-24h up | R-MMU-166663 | Initial triggering of complement | 1.37534893503075e-07 | C3/C2/C1qa/C1qc/C1qb/C1s1/Gzmm/C1ra/C4b | 9 |
| BCG/Nano-FP1-24hup | R-MMU-1236974 | ER-Phagosome pathway | 1.5753640396305e-07 | H2-M3/Tapbp/Tap2/H2-Q4/Tap1/H2-Q7/B2m/H2-K1/H2-T23/H2-Q6/H2-D1 | 11 |
| BCG/Nano-FP1-24h up | R-MMU-388841 | Costimulation by the CD28 family | 2.21893358439765e-07 | Lck/Cd3g/Ptpn6/Cd274/Pdcd1lg2/Cd86/Cd4/Icos/Cd28/Cd3e/H2-Aa/Tnfrsf14/H2-Eb1/H2-Eb2/H2-Ab1 | 15 |
| BCG/Nano-FP1-24h up | R-MMU-202427 | Phosphorylation of CD3 and TCR zeta chains | 3.70151739216777e-07 | Lck/Cd3g/Cd4/Cd3e/H2-Aa/H2-Eb1/H2-Eb2/H2-Ab1 | 8 |
| BCG/Nano-FP1-24h up | R-MMU-449147 | Signaling by Interleukins | 4.37873382830807e-07 | Lck/Il12rb1/Hck/Ptpn6/Il12b/Csf1/Il12rb2/Psme1/Cd4/Psmb8/Nfkb2/Il6/Casp1/Il18rap/Stat1/Il2ra/Il1rn/Il1b/Il1a/Il21r/Il2rg/Psmb10/Il10ra/Crlf2/Socs1/Stat2/Socs3/Osm/Il2rb/Il18bp/Psme2b/Psme2/Psmb9 | 33 |
| BCG/Nano-FP1-24h up | R-MMU-202403 | TCR signaling | 1.01963534353594e-06 | Lck/Cd3g/Lcp2/Itk/Fyb/Psme1/Cd4/Psmb8/Lat/Psmb10/Cd3e/H2-Aa/H2-Eb1/H2-Eb2/H2-Ab1/Psme2b/Psme2/Psmb9 | 18 |
| BCG/Nano -FP1-24h up | R-MMU-913531 | Interferon Signaling | 1.30414560449052e-06 | Irf9/Ptpn6/Mx2/Eif2ak2/Stat1/Ube2l6/Usp18/Uba7/Isg15/Socs1/Stat2/Ddx58/Socs3/Ifng | 14 |
| BCG/Nano-FP1-24h up | R-MMU-983170 | Antigen Presentation: Folding, assembly and peptide loading of class I MHC | 1.30414560449052e-06 | H2-M3/Tapbp/Tap2/H2-Q4/Tap1/H2-Q7/B2m/H2-K1/H2-T23/H2-Q6/H2-D1 | 11 |
| BCG/Nano-FP1-24h up | R-MMU-1236977 | Endosomal/Vacuolar pathway | 8.80215063229416e-06 | H2-M3/H2-Q4/H2-Q7/B2m/H2-K1/H2-T23/H2-Q6/H2-D1 | 8 |
| BCG/Nano-FP1-24h up | R-MMU-5668541 | TNFR2 non-canonical NF-kB pathway | 1.0804406997503e-05 | Relb/Psme1/Psmb8/Tnf/Lta/Nfkb2/Tnfsf8/Tnfrsf1b/Tnfrsf9/Tnfrsf4/Psmb10/Tnfrsf18/Tnfrsf14/Psme2b/Psme2/Psmb9 | 16 |
| BCG/Nano-FP1-24h up | R-MMU-1169408 | ISG15 antiviral mechanism | 2.76579542664069e-05 | Mx2/Eif2ak2/Stat1/Ube2l6/Usp18/Uba7/Isg15/Ddx58 | 8 |
| BCG/Nano-FP1-24h up | R-MMU-373076 | Class A/1 (Rhodopsin-like receptors) | 3.45807134511723e-05 | Cxcl16/Adora2a/Gpr132/Gpr65/C3/Htr7/Ccl20/Gpr35/Xcl1/Cxcl5/Cxcl3/Cxcl1/Cxcl9/Cxcl10/Hrh2/Ccl5/Ccr3/Ptger4/C3ar1/Bdkrb1/S1pr5/Hcar2/Cxcr6/Cxcr3/Ffar2/Cx3cr1/Ptafr/Cxcl2/Ccl19/Ccr5 | 30 |
| BCG/Nano-FP1-24h up | R-MMU-1169410 | Antiviral mechanism by IFN-stimulated genes | 5.14976180452236e-05 | Mx2/Eif2ak2/Stat1/Ube2l6/Usp18/Uba7/Isg15/Ddx58 | 8 |
| BCG/Nano-FP1-24h up | R-MMU-202424 | Downstream TCR signaling | 5.57403119648793e-05 | Lck/Cd3g/Psme1/Cd4/Psmb8/Psmb10/Cd3e/H2-Aa/H2-Eb1/H2-Eb2/H2-Ab1/Psme2b/Psme2/Psmb9 | 14 |
| BCG/Nano-FP1-24h up | R-MMU-166658 | Complement cascade | 0.00017951819202994 | C3/C2/C1qa/C1qc/C1qb/C1s1/C3ar1/Gzmm/C1ra/C4b | 10 |
| BCG/Nano-FP1-24h up | R-MMU-977606 | Regulation of Complement cascade | 0.000390517209838454 | C3/C2/C1qa/C1qc/C1qb/C1s1/C3ar1/C1ra/C4b | 9 |
| BCG/Nano-FP1-24h up | R-MMU-166786 | Creation of C4 and C2 activators | 0.000570152771298293 | C1qa/C1qc/C1qb/C1s1/C1ra | 5 |
| BCG/Nano-FP1-24h up | R-MMU-5669034 | TNFs bind their physiological receptors | 0.000662095376711932 | Lta/Tnfsf8/Tnfrsf1b/Tnfrsf9/Tnfrsf4/Tnfrsf18/Tnfrsf14 | 7 |
| BCG/Nano-FP1-24h up | R-MMU-375276 | Peptide ligand-binding receptors | 0.000952604063422129 | Cxcl16/C3/Ccl20/Xcl1/Cxcl5/Cxcl3/Cxcl1/Cxcl9/Cxcl10/Ccl5/Ccr3/C3ar1/Bdkrb1/Cxcr6/Cxcr3/Cx3cr1/Cxcl2/Ccl19/Ccr5 | 19 |
| BCG/Nano-FP1-24h up | R-MMU-909733 | Interferon alpha/beta signaling | 0.00167251816657634 | Irf9/Ptpn6/Stat1/Usp18/Socs1/Stat2/Socs3 | 7 |
| BCG/Nano-FP1-24h up | R-MMU-2172127 | DAP12 interactions | 0.00194852280204395 | Lck/Lcp2/Clec5a/Klrk1/Klrc1/Lat/Cd300e/B2m | 8 |
| BCG/Nano-FP1-24h up | R-MMU-2132295 | MHC class II antigen presentation | 0.00204361335948104 | Racgap1/Cd74/Lag3/Kif22/H2-Aa/Kif15/H2-DMa/Ctss/Cenpe/H2-Eb1/Tubb3/H2-Eb2/H2-Ab1/H2-DMb1 | 14 |
| BCG/Nano-FP1-24h up | R-MMU-983169 | Class I MHC mediated antigen processing & presentation | 0.00213928054235344 | Cdc20/Fcgr1/H2-M3/Asb2/Psme1/Tapbp/Psmb8/Tap2/Ube2l6/Rnf19b/Herc6/Trim21/Psmb10/Uba7/H2-Q4/Tap1/Socs1/Dtx3l/Socs3/H2-Q7/B2m/H2-K1/H2-T23/H2-Q6/H2-D1/Psme2b/Psme2/Psmb9 | 28 |
| BCG/Nano-FP1-24h up | R-MMU-8957275 | Post-translational protein phosphorylation | 0.00229134450156289 | Timp1/Cp/Apol7a/Csf1/Vcan/C3/Shisa5/Il6/Apol10b/Apol9a/Serpina10/Apol9b/Apol7e/C4b | 14 |
| BCG/Nano-FP1-24h up | R-MMU-2168880 | Scavenging of heme from plasma | 0.00237938626697558 | Apol7a/Hpx/Apol10b/Apol9a/Apol9b/Apol7e | 6 |
| BCG/Nano-FP1-24h up | R-MMU-622312 | Inflammasomes | 0.00238782688043994 | Pycard/Pstpip1/Nlrp3/Aim2/Nlrp1a | 5 |
| BCG/Nano-FP1-24h up | R-MMU-446652 | Interleukin-1 family signaling | 0.0026279891973592 | Psme1/Psmb8/Nfkb2/Casp1/Il18rap/Il1rn/Il1b/Il1a/Psmb10/Il18bp/Psme2b/Psme2/Psmb9 | 13 |
| BCG/Nano-FP1-24h up | R-MMU-168643 | Nucleotide-binding domain, leucine rich repeat containing receptor (NLR) signaling pathways | 0.0026279891973592 | Tnfaip3/Casp1/Pycard/Pstpip1/Nlrp3/Casp4/Aim2/Nlrp1a | 8 |
| BCG/Nano-FP1-24h up | R-MMU-202733 | Cell surface interactions at the vascular wall | 0.00396139515620915 | Lck/Ptpn6/Dok2/Slc7a8/Cd74/Fcamr/Selp/Sele/Procr/Slc7a11/Slc7a5/Spn/Fcer1g | 13 |
| BCG/Nano-FP1-24h up | R-MMU-877300 | Interferon gamma signaling | 0.00403494518802666 | Ptpn6/Stat1/Socs1/Socs3/Ifng | 5 |
| BCG/Nano-FP1-24h up | R-MMU-2173782 | Binding and Uptake of Ligands by Scavenger Receptors | 0.00403494518802666 | Apol7a/Msr1/Hpx/Apol10b/Apol9a/Apol9b/Apol7e | 7 |
| BCG/Nano-FP1-24h up | R-MMU-500792 | GPCR ligand binding | 0.00429378520941529 | Cxcl16/Adora2a/Gpr132/Gpr65/C3/Htr7/Ccl20/Gpr35/Xcl1/Cxcl5/Cxcl3/Cxcl1/Cxcl9/Cxcl10/Hrh2/Ccl5/Ccr3/Ptger4/C3ar1/Bdkrb1/S1pr5/Hcar2/Cxcr6/Cxcr3/Ffar2/Cx3cr1/Ptafr/Cxcl2/Ccl19/Ccr5 | 30 |
| BCG/Nano-FP1-24h up | R-MMU-5607764 | CLEC7A (Dectin-1) signaling | 0.00526058114876992 | Relb/Psme1/Psmb8/Nfkb2/Il1b/Nfatc2/Pycard/Psmb10/Psme2b/Psme2/Psmb9 | 11 |
| BCG/Nano-FP1-24h up | R-MMU-877312 | Regulation of IFNG signaling | 0.00645700050334659 | Stat1/Socs1/Socs3/Ifng | 4 |
| BCG/Nano-FP1-24h up | R-MMU-381426 | Regulation of Insulin-like Growth Factor (IGF) transport and uptake by Insulin-like Growth Factor Binding Proteins (IGFBPs) | 0.00672750628456018 | Timp1/Cp/Apol7a/Csf1/Vcan/C3/Shisa5/Il6/Apol10b/Apol9a/Serpina10/Apol9b/Apol7e/C4b | 14 |
| BCG/Nano-FP1-24h up | R-MMU-451927 | Interleukin-2 family signaling | 0.00820049823538354 | Lck/Ptpn6/Stat1/Il2ra/Il21r/Il2rg/Il2rb | 7 |
| BCG/Nano-FP1-24h up | R-MMU-2424491 | DAP12 signaling | 0.00820049823538354 | Lck/Lcp2/Klrk1/Klrc1/Lat/B2m | 6 |
| BCG/Nano-FP1-24h up | R-MMU-9020558 | Interleukin-2 signaling | 0.0085717093593358 | Lck/Il2ra/Il2rg/Il2rb | 4 |
| BCG/Nano-FP1-24h up | R-MMU-174178 | APC/C:Cdh1 mediated degradation of Cdc20 and other APC/C:Cdh1 targeted proteins in late mitosis/early G1 | 0.00935968455553951 | Cdc20/Aurkb/Psme1/Psmb8/Plk1/Psmb10/Psme2b/Psme2/Psmb9 | 9 |
| BCG/Nano-FP1-24h up | R-MMU-5607761 | Dectin-1 mediated noncanonical NF-kB signaling | 0.0102116176685002 | Relb/Psme1/Psmb8/Nfkb2/Psmb10/Psme2b/Psme2/Psmb9 | 8 |
| BCG/Nano-FP1-24h up | R-MMU-5676590 | NIK-->noncanonical NF-kB signaling | 0.0102116176685002 | Relb/Psme1/Psmb8/Nfkb2/Psmb10/Psme2b/Psme2/Psmb9 | 8 |
| BCG/Nano-FP1-24h up | R-MMU-174143 | APC/C-mediated degradation of cell cycle proteins | 0.0102116176685002 | Cdc20/Aurkb/Psme1/Cdkn1a/Psmb8/Plk1/Psmb10/Psme2b/Psme2/Psmb9 | 10 |
| BCG/Nano-FP1-24h up | R-MMU-453276 | Regulation of mitotic cell cycle | 0.0102116176685002 | Cdc20/Aurkb/Psme1/Cdkn1a/Psmb8/Plk1/Psmb10/Psme2b/Psme2/Psmb9 | 10 |
| BCG/Nano-FP1-24h up | R-MMU-8852276 | The role of GTSE1 in G2/M progression after G2 checkpoint | 0.0102116176685002 | Psme1/Cdkn1a/Psmb8/Plk1/Psmb10/Tubb3/Psme2b/Psme2/Psmb9 | 9 |
| BCG/Nano-FP1-24h up | R-MMU-2029481 | FCGR activation | 0.0102116176685002 | Cd3g/Hck/Fcgr1/Fcgr4 | 4 |
| BCG/Nano-FP1-24h up | R-MMU-416700 | Other semaphorin interactions | 0.0102116176685002 | Sema4d/Sema4a/Cd72/Sema7a | 4 |
| BCG/Nano-FP1-24h up | R-MMU-2454202 | Fc epsilon receptor (FCERI) signaling | 0.013700849280656 | Lcp2/Itk/Psme1/Psmb8/Nfatc2/Lat/Psmb10/Lat2/Fcer1g/Psme2b/Psme2/Psmb9 | 12 |
| BCG/Nano-FP1-24h up | R-MMU-1236978 | Cross-presentation of soluble exogenous antigens (endosomes) | 0.0168903397290683 | Fcgr1/Psme1/Psmb8/Psmb10/Psme2b/Psme2/Psmb9 | 7 |
| BCG/Nano-FP1-24h up | R-MMU-9020702 | Interleukin-1 signaling | 0.0176052784498423 | Psme1/Psmb8/Nfkb2/Il1rn/Il1b/Il1a/Psmb10/Psme2b/Psme2/Psmb9 | 10 |
| BCG/Nano-FP1-24h up | R-MMU-69601 | Ubiquitin Mediated Degradation of Phosphorylated Cdc25A | 0.0176052784498423 | Psme1/Psmb8/Psmb10/Chek1/Psme2b/Psme2/Psmb9 | 7 |
| BCG/Nano-FP1-24h up | R-MMU-69610 | p53-Independent DNA Damage Response | 0.0176052784498423 | Psme1/Psmb8/Psmb10/Chek1/Psme2b/Psme2/Psmb9 | 7 |
| BCG/Nano-FP1-24h up | R-MMU-69613 | p53-Independent G1/S DNA damage checkpoint | 0.0176052784498423 | Psme1/Psmb8/Psmb10/Chek1/Psme2b/Psme2/Psmb9 | 7 |
| BCG/Nano-FP1-24h up | R-MMU-1168372 | Downstream signaling events of B Cell Receptor (BCR) | 0.0176052784498423 | Psme1/Nfkbie/Psmb8/Rasgrp1/Nfatc2/Psmb10/Psme2b/Psme2/Psmb9 | 9 |
| BCG/Nano-FP1-24h up | R-MMU-5621481 | C-type lectin receptors (CLRs) | 0.0176052784498423 | Relb/Psme1/Psmb8/Nfkb2/Il1b/Nfatc2/Pycard/Psmb10/Psme2b/Psme2/Psmb9 | 11 |
| BCG/Nano-FP1-24h up | R-MMU-69615 | G1/S DNA Damage Checkpoints | 0.0177518994516096 | Psme1/Cdkn1a/Psmb8/Psmb10/Chek1/Psme2b/Psme2/Psmb9 | 8 |
| BCG/Nano-FP1-24h up | R-MMU-176408 | Regulation of APC/C activators between G1/S and early anaphase | 0.0183784075646219 | Cdc20/Psme1/Cdkn1a/Psmb8/Plk1/Psmb10/Psme2b/Psme2/Psmb9 | 9 |
| BCG/Nano-FP1-24h up | R-MMU-5658442 | Regulation of RAS by GAPs | 0.0207183064487261 | Rasa4/Psme1/Psmb8/Psmb10/Rasal3/Psme2b/Psme2/Psmb9 | 8 |
| BCG/Nano-FP1-24h up | R-MMU-68867 | Assembly of the pre-replicative complex | 0.0207183064487261 | Mcm5/Cdc6/Psme1/Psmb8/Psmb10/Psme2b/Psme2/Psmb9 | 8 |
| BCG/Nano-FP1-24h up | R-MMU-2467813 | Separation of Sister Chromatids | 0.0218033143095619 | Cdc20/Birc5/Aurkb/Psme1/Psmb8/Cdca5/Plk1/Psmb10/Zwilch/Cenpe/Espl1/Tubb3/Psme2b/Psme2/Psmb9 | 15 |
| BCG/Nano-FP1-24h up | R-MMU-174113 | SCF-beta-TrCP mediated degradation of Emi1 | 0.0221394509561107 | Cdc20/Psme1/Psmb8/Psmb10/Psme2b/Psme2/Psmb9 | 7 |
| BCG/Nano-FP1-24h up | R-MMU-69002 | DNA Replication Pre-Initiation | 0.0221394509561107 | Mcm5/Pole/Cdc6/Psme1/Psmb8/Psmb10/Psme2b/Psme2/Psmb9 | 9 |
| BCG/Nano-FP1-24h up | R-MMU-68882 | Mitotic Anaphase | 0.0244284285397527 | Cdc20/Birc5/Aurkb/Psme1/Psmb8/Cdca5/Plk1/Psmb10/Zwilch/Cenpe/Espl1/Tubb3/Psme2b/Psme2/Psmb9 | 15 |
| BCG/Nano-FP1-24h up | R-MMU-2555396 | Mitotic Metaphase and Anaphase | 0.0249219639550229 | Cdc20/Birc5/Aurkb/Psme1/Psmb8/Cdca5/Plk1/Psmb10/Zwilch/Cenpe/Espl1/Tubb3/Psme2b/Psme2/Psmb9 | 15 |
| BCG/Nano-FP1-24h up | R-MMU-351202 | Metabolism of polyamines | 0.0249219639550229 | Arg1/Psme1/Psmb8/Gatm/Psmb10/Ass1/Psme2b/Psme2/Psmb9 | 9 |
| BCG/Nano-FP1-24h up | R-MMU-68949 | Orc1 removal from chromatin | 0.0249219639550229 | Mcm5/Cdc6/Psme1/Psmb8/Psmb10/Psme2b/Psme2/Psmb9 | 8 |
| BCG/Nano-FP1-24h up | R-MMU-197264 | Nicotinamide salvaging | 0.0274567451763786 | Nampt/Parp9/Parp14/Parp10 | 4 |
| BCG/Nano-FP1-24h up | R-MMU-2871809 | FCERI mediated Ca+2 mobilization | 0.0276534031308281 | Lcp2/Itk/Nfatc2/Lat/Fcer1g | 5 |
| BCG/Nano-FP1-24h up | R-MMU-983705 | Signaling by the B Cell Receptor (BCR) | 0.027936019163378 | Ptpn6/Psme1/Nfkbie/Psmb8/Rasgrp1/Nfatc2/Psmb10/Psme2b/Psme2/Psmb9 | 10 |
| BCG/Nano-FP1-24h up | R-MMU-69206 | G1/S Transition | 0.0296019457428833 | Mcm5/Pole/Cdc6/Psme1/Cdkn1a/Psmb8/Psmb10/Psme2b/Psme2/Psmb9 | 10 |
| BCG/Nano-FP1-24h up | R-MMU-68827 | CDT1 association with the CDC6:ORC:origin complex | 0.0296262219144783 | Cdc6/Psme1/Psmb8/Psmb10/Psme2b/Psme2/Psmb9 | 7 |
| BCG/Nano-FP1-24h up | R-MMU-5656169 | Termination of translesion DNA synthesis | 0.03055715145572 | Pole/Ube2l6/Uba7/Isg15/Pclaf | 5 |
| BCG/Nano-FP1-24h up | R-MMU-187577 | SCF(Skp2)-mediated degradation of p27/p21 | 0.0319129146900494 | Psme1/Cdkn1a/Psmb8/Psmb10/Psme2b/Psme2/Psmb9 | 7 |
| BCG/Nano-FP1-24h up | R-MMU-176814 | Activation of APC/C and APC/C:Cdc20 mediated degradation of mitotic proteins | 0.0320245671053099 | Cdc20/Psme1/Psmb8/Plk1/Psmb10/Psme2b/Psme2/Psmb9 | 8 |
| BCG/Nano-FP1-24h up | R-MMU-844456 | The NLRP3 inflammasome | 0.0324337478884828 | Pycard/Pstpip1/Nlrp3 | 3 |
| BCG/Nano-FP1-24h up | R-MMU-9020591 | Interleukin-12 signaling | 0.0324337478884828 | Il12rb1/Il12b/Il12rb2 | 3 |
| BCG/Nano-FP1-24h up | R-MMU-9020958 | Interleukin-21 signaling | 0.0324337478884828 | Stat1/Il21r/Il2rg | 3 |
| BCG/Nano-FP1-24h up | R-MMU-69620 | Cell Cycle Checkpoints | 0.0352462524944345 | Mcm5/Cdc20/Cdc6/Birc5/Aurkb/Psme1/Cdkn1a/Psmb8/Plk1/Psmb10/Chek1/Zwilch/Rmi2/Exo1/Clspn/Cenpe/Psme2b/Psme2/Psmb9 | 19 |
| BCG/Nano-FP1-24h up | R-MMU-447115 | Interleukin-12 family signaling | 0.0352462524944345 | Il12rb1/Il12b/Il12rb2/Stat1 | 4 |
| BCG/Nano-FP1-24h up | R-MMU-168928 | DDX58/IFIH1-mediated induction of interferon-alpha/beta | 0.0368988934592201 | Tnfaip3/Nfkb2/Irf7/Ddx58/Ikbke | 5 |
| BCG/Nano-FP1-24h up | R-MMU-1059683 | Interleukin-6 signaling | 0.0408356399808831 | Il6/Stat1/Socs3 | 3 |
| BCG/Nano-FP1-24h up | R-MMU-418594 | G alpha (i) signalling events | 0.0408356399808831 | Cxcl16/Ccl4/Guca1a/C3/Ccr1/Ccl20/Cxcl5/Cxcl3/Cxcl1/Cxcl9/Cxcl10/Ccl5/Ccr3/C3ar1/Bdkrb1/S1pr5/Hcar2/Cxcr6/Ccr2/Cxcr3/Cx3cr1/Cxcl2/Ccl19/Ccr5 | 24 |
| BCG/Nano-FP1-24h up | R-MMU-69563 | p53-Dependent G1 DNA Damage Response | 0.0408356399808831 | Psme1/Cdkn1a/Psmb8/Psmb10/Psme2b/Psme2/Psmb9 | 7 |
| BCG/Nano-FP1-24h up | R-MMU-69580 | p53-Dependent G1/S DNA damage checkpoint | 0.0408356399808831 | Psme1/Cdkn1a/Psmb8/Psmb10/Psme2b/Psme2/Psmb9 | 7 |
| BCG/Nano-FP1-24h up | R-MMU-73893 | DNA Damage Bypass | 0.0426127231878583 | Pole/Ube2l6/Uba7/Isg15/Dtl/Pclaf | 6 |
| BCG/Nano-FP1-24h up | R-MMU-114604 | GPVI-mediated activation cascade | 0.0449098616106564 | Lck/Lcp2/Ptpn6/Lat/Fcer1g | 5 |
| BCG/Nano-FP1-24h up | R-MMU-69229 | Ubiquitin-dependent degradation of Cyclin D1 | 0.0456381749811977 | Psme1/Psmb8/Psmb10/Psme2b/Psme2/Psmb9 | 6 |
| BCG/Nano-FP1-24h up | R-MMU-75815 | Ubiquitin-dependent degradation of Cyclin D | 0.0456381749811977 | Psme1/Psmb8/Psmb10/Psme2b/Psme2/Psmb9 | 6 |
| BCG/Nano-FP1-24h up | R-MMU-1169091 | Activation of NF-kappaB in B cells | 0.0456381749811977 | Psme1/Nfkbie/Psmb8/Psmb10/Psme2b/Psme2/Psmb9 | 7 |
| BCG/Nano-FP1-24h up | R-MMU-174154 | APC/C:Cdc20 mediated degradation of Securin | 0.0456381749811977 | Cdc20/Psme1/Psmb8/Psmb10/Psme2b/Psme2/Psmb9 | 7 |
| BCG/Nano-FP1-24h up | R-MMU-389359 | CD28 dependent Vav1 pathway | 0.0480029417590308 | Lck/Cd86/Cd28 | 3 |
| BCG/Nano-FP1-24h up | R-MMU-349425 | Autodegradation of the E3 ubiquitin ligase COP1 | 0.0480029417590308 | Psme1/Psmb8/Psmb10/Psme2b/Psme2/Psmb9 | 6 |
| BCG/Nano-FP1-24h up | R-MMU-8939902 | Regulation of RUNX2 expression and activity | 0.0480029417590308 | Psme1/Psmb8/Psmb10/Psme2b/Psme2/Psmb9 | 6 |

ID= Reactome pathway ID. P.adjust = adjusted p-value; indicates the significance of the enrichment. Count = number of genes from the submitted list found in the pathway.

**Table S3**. Reactome Pathway Enrichment results obtained from BAL analysis

| Cluster | ID | Description | p.adjust | geneID | Count |
| --- | --- | --- | --- | --- | --- |
| 2 weeks BCG/Nano-FP1 downregulated | R-MMU-2500257 | Resolution of Sister Chromatid Cohesion | 0.00124878447131451 | Cdc20/Birc5/Cdk1/Aurkb/Sgo1/Cdca5/Nuf2/Bub1/Kif2c/Cdca8/Kntc1/Plk1/Cenpi/Cenpn/Ccnb2/Zwilch/Cenpt/Tubb4b/Bub1b/Ccnb1/Nsl1/Cenpm | 22 |
| 2 weeks BCG/Nano-FP1 downregulated | R-MMU-390247 | Beta-oxidation of very long chain fatty acids | 0.00124878447131451 | Acaa1b/Acox1/Eci2/Ehhadh/Hsd17b4/Acaa1a/Acot4 | 7 |
| 2 weeks BCG/Nano-FP1 downregulated | R-MMU-68877 | Mitotic Prometaphase | 0.00262497144115307 | Prkar2b/Cdc20/Ncapg/Birc5/Cdk1/Aurkb/Sgo1/Cdca5/Nek2/Nuf2/Bub1/Smc2/Kif2c/Cdca8/Kntc1/Plk1/Cenpi/Cenpn/Ccnb2/Zwilch/Ncaph/Cenpt/Tubb4b/Ncapd2/Bub1b/Ccnb1/Nsl1/Cenpm | 28 |
| 2 weeks BCG/Nano-FP1 downregulated | R-MMU-69620 | Cell Cycle Checkpoints | 0.00423578390220068 | Ube2c/Mcm5/Cdc20/Brca1/Cdc6/Birc5/Cdk1/Aurkb/Sgo1/Mcm10/Nuf2/Bub1/Ccna2/Ccne2/Orc1/Kif2c/Cdca8/Kntc1/Mcm7/Plk1/Cenpi/Cenpn/Ccnb2/Zwilch/Brip1/Cenpt/Exo1/Bub1b/Ccnb1/Clspn/Cdc25c/H2afx/Nsl1/Cenpm | 34 |
| 2 weeks BCG/Nano-FP1 downregulated | R-MMU-141424 | Amplification of signal from the kinetochores | 0.00423578390220068 | Cdc20/Birc5/Aurkb/Sgo1/Nuf2/Bub1/Kif2c/Cdca8/Kntc1/Plk1/Cenpi/Cenpn/Zwilch/Cenpt/Bub1b/Nsl1/Cenpm | 17 |
| 2 weeks BCG/Nano-FP1 downregulated | R-MMU-141444 | Amplification of signal from unattached kinetochores via a MAD2 inhibitory signal | 0.00423578390220068 | Cdc20/Birc5/Aurkb/Sgo1/Nuf2/Bub1/Kif2c/Cdca8/Kntc1/Plk1/Cenpi/Cenpn/Zwilch/Cenpt/Bub1b/Nsl1/Cenpm | 17 |
| 2 weeks BCG/Nano-FP1 downregulated | R-MMU-5663220 | RHO GTPases Activate Formins | 0.00423578390220068 | Rhoc/Cdc20/Birc5/Aurkb/Diaph3/Sgo1/Nuf2/Bub1/Kif2c/Cdca8/Kntc1/Actb/Plk1/Cenpi/Cenpn/Zwilch/Cenpt/Tubb4b/Bub1b/Nsl1/Cenpm | 21 |
| 2 weeks BCG/Nano-FP1 downregulated | R-MMU-69273 | Cyclin A/B1/B2 associated events during G2/M transition | 0.00458915819758449 | Foxm1/Cdk1/Cdc25b/Ccna2/Plk1/Ccnb2/Ccnb1/Cdc25c | 8 |
| 2 weeks BCG/Nano-FP1 downregulated | R-MMU-69618 | Mitotic Spindle Checkpoint | 0.00659627312824909 | Ube2c/Cdc20/Birc5/Aurkb/Sgo1/Nuf2/Bub1/Kif2c/Cdca8/Kntc1/Plk1/Cenpi/Cenpn/Zwilch/Cenpt/Bub1b/Nsl1/Cenpm | 18 |
| 2 weeks BCG/Nano-FP1 downregulated | R-MMU-68886 | M Phase | 0.0134087074355814 | Ube2c/Emd/Prkar2b/Kif20a/Cdc20/Ncapg/Birc5/Cdk1/Lpin1/Aurkb/Vrk1/Sgo1/Cdca5/Nek2/Nuf2/Mastl/Bub1/Lmna/Smc2/Kif2c/Cdca8/Kntc1/Plk1/Cenpi/Cenpn/Ccnb2/Kif23/Zwilch/Ncaph/Cenpt/Tubb4b/Ncapd2/Bub1b/Ccnb1/H2afx/Espl1/Nsl1/Cenpm/Hist1h3c/Hist1h2ap | 40 |
| 2 weeks BCG/Nano-FP1 downregulated | R-MMU-8978868 | Fatty acid metabolism | 0.0164257788806258 | Pon1/Ggt5/Acaa1b/Acsf3/Ppt2/Alox15/Acox1/Aloxe3/Acot2/Elovl2/Eci2/Ehhadh/Abcc1/Hsd17b4/Cpt1a/Alox5/Hadha/Pecr/Ptges2/Cpt2/Cyp4b1/Acaa1a/Acot4/Alox5ap/Acot1 | 25 |
| 2 weeks BCG/Nano-FP1 downregulated | R-MMU-390918 | Peroxisomal lipid metabolism | 0.0175380884743921 | Acaa1b/Acox1/Eci2/Ehhadh/Hsd17b4/Pecr/Acaa1a/Acot4 | 8 |
| 2 weeks BCG/Nano-FP1 downregulated | R-MMU-195258 | RHO GTPase Effectors | 0.0175380884743921 | Rhoc/Cdc20/Birc5/Aurkb/Diaph3/Sgo1/Nuf2/Pkn3/Bub1/Iqgap3/Kif2c/Cdca8/Kntc1/Cit/Actb/Rhpn2/Myh14/Plk1/Cenpi/Flna/Cenpn/Zwilch/Cenpt/Tubb4b/Prc1/Bub1b/Kif14/Cdc25c/H2afx/Nsl1/Cenpm/Hist1h3c/Hist1h2ap | 33 |
| 2 weeks BCG/Nano-FP1 downregulated | R-MMU-9033241 | Peroxisomal protein import | 0.0214062807955926 | Acaa1b/Acox1/Acot2/Eci2/Ehhadh/Hsd17b4/Pecr/Hmgcl/Acaa1a/Acot4/Ech1/Acot1 | 12 |
| 2 weeks BCG/Nano-FP1 downregulated | R-MMU-194315 | Signaling by Rho GTPases | 0.0323463570087494 | Rhoc/Arhgef40/Cdc20/Birc5/Rac3/Aurkb/Net1/Diaph3/Sgo1/Nuf2/Pkn3/Bub1/Ect2/Iqgap3/Kif2c/Cdca8/Kntc1/Cit/Actb/Rhpn2/Myh14/Plk1/Cenpi/Flna/Cenpn/Zwilch/Gmip/Cenpt/Tubb4b/Syde2/Prc1/Myo9a/Bub1b/Kif14/Cdc25c/Arhgap23/H2afx/Nsl1/Cenpm/Hist1h3c/Arhgdig/Hist1h2ap | 42 |
| 2 weeks BCG/Nano-FP1 downregulated | R-MMU-2046104 | alpha-linolenic (omega3) and linoleic (omega6) acid metabolism | 0.0435226548674425 | Acaa1b/Acox1/Elovl2/Hsd17b4/Acaa1a | 5 |
| 2 weeks BCG/Nano-FP1 downregulated | R-MMU-2046106 | alpha-linolenic acid (ALA) metabolism | 0.0435226548674425 | Acaa1b/Acox1/Elovl2/Hsd17b4/Acaa1a | 5 |
| 2 weeks BCG/Nano-FP1 downregulated | R-MMU-9609507 | Protein localization | 0.0468110494566927 | Acaa1b/Acox1/Acot2/Eci2/Ehhadh/Hsd17b4/Pecr/Hmgcl/Acaa1a/Acot4/Ech1/Acot1 | 12 |
| 2 weeks BCG/Nano-FP1 downregulated | R-MMU-983189 | Kinesins | 0.0468110494566927 | Kif20a/Kif11/Kif21a/Kif2c/Kif22/Kif23/Kif4/Tubb4b/Kif18b/Kifc1 | 10 |
| 2 weeks BCG/Nano-FP1 downregulated | R-MMU-68882 | Mitotic Anaphase | 0.049619069743887 | Ube2c/Cdc20/Birc5/Aurkb/Vrk1/Sgo1/Cdca5/Nuf2/Bub1/Kif2c/Cdca8/Kntc1/Plk1/Cenpi/Cenpn/Zwilch/Cenpt/Tubb4b/Bub1b/Espl1/Nsl1/Cenpm | 22 |
| 2 weeks BCG/Nano-FP1 downregulated | R-MMU-4419969 | Depolymerisation of the Nuclear Lamina | 0.049619069743887 | Emd/Cdk1/Lpin1/Lmna/Ccnb1 | 5 |
| 2 weeks BCG/Nano-FP1 downregulated | R-MMU-2555396 | Mitotic Metaphase and Anaphase | 0.049619069743887 | Ube2c/Cdc20/Birc5/Aurkb/Vrk1/Sgo1/Cdca5/Nuf2/Bub1/Kif2c/Cdca8/Kntc1/Plk1/Cenpi/Cenpn/Zwilch/Cenpt/Tubb4b/Bub1b/Espl1/Nsl1/Cenpm | 22 |
| 2 weeks BCG/Nano-FP1 upregulated | R-MMU-198933 | Immunoregulatory interactions between a Lymphoid and a non-Lymphoid cell | 1.94892887932216e-18 | Icam2/Itgb7/Cd3g/Sh2d1a/Cd247/Slamf6/H2-M3/H2-M2/Cd40/Cd96/Cd200/Trem2/C3/Ifitm3/Col3a1/Itga4/Cd1d1/Klrk1/Pianp/Cd19/Cd40lg/Crtam/Cd3e/Cd3d/Cd226/Cd300a/H2-Q4/Slamf7/Pvr/Cd8b1/Cd8a/H2-Q7/Ifitm2/B2m/H2-K1/Cd200r4/H2-T23/Treml2/H2-Q6/H2-D1/Cd200r2 | 41 |
| 2 weeks BCG/Nano-FP1 upregulated | R-MMU-202430 | Translocation of ZAP-70 to Immunological synapse | 8.06371645897655e-12 | Lck/Cd3g/Cd247/Cd4/Zap70/Ptpn22/Cd3e/Cd3d/H2-Aa/H2-Eb1/H2-Eb2/H2-Ab1 | 12 |
| 2 weeks BCG/Nano-FP1 upregulated | R-MMU-202433 | Generation of second messenger molecules | 5.56815384103429e-10 | Lck/Cd3g/Cd247/Plcg1/Itk/Fyb/Cd4/Zap70/Lat/Cd3e/Cd3d/H2-Aa/H2-Eb1/H2-Eb2/H2-Ab1 | 15 |
| 2 weeks BCG/Nano-FP1 upregulated | R-MMU-389948 | PD-1 signaling | 5.57965757035919e-09 | Lck/Cd3g/Cd247/Cd274/Cd4/Pdcd1/Cd3e/Cd3d/H2-Aa/H2-Eb1/H2-Eb2/H2-Ab1 | 12 |
| 2 weeks BCG/Nano-FP1 upregulated | R-MMU-202427 | Phosphorylation of CD3 and TCR zeta chains | 1.33222970065202e-08 | Lck/Cd3g/Cd247/Cd4/Ptpn22/Cd3e/Cd3d/H2-Aa/H2-Eb1/H2-Eb2/H2-Ab1 | 11 |
| 2 weeks BCG/Nano-FP1 upregulated | R-MMU-388841 | Costimulation by the CD28 family | 1.90120396426229e-08 | Lck/Cd3g/Cd247/Cd274/Akt3/Cd86/Cd4/Icos/Ctla4/Cd28/Pdcd1/Src/Pik3ca/Cd3e/Cd3d/Trib3/H2-Aa/Tnfrsf14/Btla/H2-Eb1/H2-Eb2/H2-Ab1 | 22 |
| 2 weeks BCG/Nano-FP1 upregulated | R-MMU-1280215 | Cytokine Signaling in Immune system | 3.56966856822034e-08 | Lck/Il12rb1/Irf9/Ebi3/Il7r/Tnfsf14/Tnfrsf13b/Csf1/Il10/Plcg1/Cd40/Ifngr1/Irak3/Il9r/Sdc1/Tnfsf11/Psme1/Ifngr2/Il10rb/Ifnar2/Il15ra/Cd4/Psmb8/Ltb/Jak2/Flnb/Il18rap/Il18r1/Stat1/Pik3ca/Il21/Tnfsf8/Tec/Usp18/Cd27/Psmd8/Il21r/Cd40lg/Il2rg/Dusp4/Birc3/Sos2/Tnfsf9/Socs1/Pik3cd/Ripk2/Stx3/Tnfrsf14/Tnfsf15/Socs3/Ifng/Camk2b/Osm/Irak2/Stat4/Tnfrsf13c/Il2rb/Il3ra/Il18bp/Il11ra1/Psme2b/Psme2/Edaradd/Psmb9/Flt3l | 65 |
| 2 weeks BCG/Nano-FP1 upregulated | R-MMU-202403 | TCR signaling | 1.04075533856544e-06 | Lck/Cd3g/Cd247/Plcg1/Itk/Fyb/Psme1/Cd4/Psmb8/Zap70/Prkcq/Pik3ca/Ptpn22/Psmd8/Lat/Cd3e/Cd3d/Malt1/H2-Aa/Ripk2/H2-Eb1/H2-Eb2/H2-Ab1/Psme2b/Psme2/Psmb9 | 26 |
| 2 weeks BCG/Nano-FP1 upregulated | R-MMU-451927 | Interleukin-2 family signaling | 5.05384614896005e-05 | Lck/Il9r/Il15ra/Jak2/Stat1/Pik3ca/Il21/Il21r/Il2rg/Sos2/Pik3cd/Stat4/Il2rb/Il3ra | 14 |
| 2 weeks BCG/Nano-FP1 upregulated | R-MMU-202424 | Downstream TCR signaling | 0.00016409087139762 | Lck/Cd3g/Cd247/Psme1/Cd4/Psmb8/Prkcq/Pik3ca/Psmd8/Cd3e/Cd3d/Malt1/H2-Aa/Ripk2/H2-Eb1/H2-Eb2/H2-Ab1/Psme2b/Psme2/Psmb9 | 20 |
| 2 weeks BCG/Nano-FP1 upregulated | R-MMU-877312 | Regulation of IFNG signaling | 0.000190735310412239 | Ifngr1/Ifngr2/Jak2/Stat1/Socs1/Socs3/Ifng | 7 |
| 2 weeks BCG/Nano-FP1 upregulated | R-MMU-1236974 | ER-Phagosome pathway | 0.000190735310412239 | H2-M3/H2-M2/Tapbp/H2-Q4/Tap1/H2-Q7/B2m/H2-K1/H2-T23/H2-Q6/H2-D1 | 11 |
| 2 weeks BCG/Nano-FP1 upregulated | R-MMU-1236977 | Endosomal/Vacuolar pathway | 0.000268264320791659 | H2-M3/H2-M2/H2-Q4/H2-Q7/B2m/H2-K1/H2-T23/H2-Q6/H2-D1 | 9 |
| 2 weeks BCG/Nano-FP1 upregulated | R-MMU-877300 | Interferon gamma signaling | 0.000575069863013198 | Ifngr1/Ifngr2/Jak2/Stat1/Socs1/Socs3/Ifng/Camk2b | 8 |
| 2 weeks BCG/Nano-FP1 upregulated | R-MMU-76002 | Platelet activation, signaling and aggregation | 0.000736000540889074 | Gna12/Lck/Vwf/Psap/Arrb1/Igf1/Srgn/Itgb3/Vcl/Ctsw/Cd63/Pdgfa/Prkcq/Rasgrp1/Src/Pik3ca/Gnb4/P2ry1/Ecm1/Pdpn/Hgf/Itpr1/Lat/Gng11/Rasgrp2/Lgals3bp/Dgkh/Gng12/Endod1/F13a1/Orm1/Itpr3/Gng2/Prkce/Lhfpl2/F2r/Actn2/Prkcb/Serpina3f | 39 |
| 2 weeks BCG/Nano-FP1 upregulated | R-MMU-5668541 | TNFR2 non-canonical NF-kB pathway | 0.000736000540889074 | Tnfsf14/Tnfrsf13b/Cd40/Tnfsf11/Psme1/Psmb8/Ltb/Tnfsf8/Cd27/Psmd8/Cd40lg/Birc3/Tnfsf9/Tnfrsf14/Tnfsf15/Tnfrsf13c/Psme2b/Psme2/Edaradd/Psmb9 | 20 |
| 2 weeks BCG/Nano-FP1 upregulated | R-MMU-449147 | Signaling by Interleukins | 0.00104901404477814 | Lck/Il12rb1/Ebi3/Il7r/Csf1/Il10/Irak3/Il9r/Sdc1/Psme1/Il10rb/Il15ra/Cd4/Psmb8/Jak2/Il18rap/Il18r1/Stat1/Pik3ca/Il21/Tec/Psmd8/Il21r/Il2rg/Dusp4/Sos2/Socs1/Pik3cd/Ripk2/Stx3/Socs3/Osm/Irak2/Stat4/Il2rb/Il3ra/Il18bp/Il11ra1/Psme2b/Psme2/Psmb9/Flt3l | 42 |
| 2 weeks BCG/Nano-FP1 upregulated | R-MMU-2424491 | DAP12 signaling | 0.00130448814703487 | Lck/Plcg1/Trem2/Pik3ca/Klrk1/Klrd1/Klrc1/Lat/Klrc2/B2m | 10 |
| 2 weeks BCG/Nano-FP1 upregulated | R-MMU-983170 | Antigen Presentation: Folding, assembly and peptide loading of class I MHC | 0.00130448814703487 | H2-M3/H2-M2/Tapbp/H2-Q4/Tap1/H2-Q7/B2m/H2-K1/H2-T23/H2-Q6/H2-D1 | 11 |
| 2 weeks BCG/Nano-FP1 upregulated | R-MMU-380108 | Chemokine receptors bind chemokines | 0.00204519588597526 | Cxcl16/Xcl1/Cxcl9/Ccr9/Cxcl10/Ccl5/Ccr6/Ccr8/Ccrl2/Cxcr5/Cxcr6/Cxcr3/Ccr5 | 13 |
| 2 weeks BCG/Nano-FP1 upregulated | R-MMU-166663 | Initial triggering of complement | 0.00383360606894382 | C3/C2/C1qa/C1qc/C1qb/C1s1/C1ra | 7 |
| 2 weeks BCG/Nano-FP1 upregulated | R-MMU-5676594 | TNF receptor superfamily (TNFSF) members mediating non-canonical NF-kB pathway | 0.00383360606894382 | Tnfsf14/Cd40/Tnfsf11/Ltb/Cd40lg/Birc3/Tnfrsf13c | 7 |
| 2 weeks BCG/Nano-FP1 upregulated | R-MMU-2172127 | DAP12 interactions | 0.00404868540373165 | Lck/Plcg1/Trem2/Pik3ca/Clec5a/Klrk1/Klrd1/Klrc1/Lat/Klrc2/B2m | 11 |
| 2 weeks BCG/Nano-FP1 upregulated | R-MMU-1236975 | Antigen processing-Cross presentation | 0.00421606820165985 | H2-M3/H2-M2/Psme1/Tapbp/Psmb8/Psmd8/H2-Q4/Tap1/H2-Q7/B2m/H2-K1/H2-T23/H2-Q6/H2-D1/Psme2b/Psme2/Psmb9 | 17 |
| 2 weeks BCG/Nano-FP1 upregulated | R-MMU-977606 | Regulation of Complement cascade | 0.00471881882885609 | C3/C2/Cd55/Cr2/Cd19/C1qa/C1qc/C1qb/C1s1/C3ar1/C1ra | 11 |
| 2 weeks BCG/Nano-FP1 upregulated | R-MMU-983705 | Signaling by the B Cell Receptor (BCR) | 0.00977964104691416 | Cd79a/Psme1/Psmb8/Rasgrp1/Nfatc2/Itpr1/Psmd8/Cd19/Malt1/Pik3cd/Cd79b/Itpr3/Prkcb/Blnk/Rasgrp3/Psme2b/Psme2/Psmb9 | 18 |
| 2 weeks BCG/Nano-FP1 upregulated | R-MMU-5669034 | TNFs bind their physiological receptors | 0.0101214643647507 | Tnfrsf13b/Tnfsf11/Tnfsf8/Cd27/Tnfsf9/Tnfrsf14/Tnfsf15/Edaradd | 8 |
| 2 weeks BCG/Nano-FP1 upregulated | R-MMU-9020958 | Interleukin-21 signaling | 0.0101214643647507 | Stat1/Il21/Il21r/Il2rg/Stat4 | 5 |
| 2 weeks BCG/Nano-FP1 upregulated | R-MMU-216083 | Integrin cell surface interactions | 0.0101214643647507 | Icam2/Itgb7/Col18a1/Vwf/Itgae/Itga2/Itgb3/Col3a1/Col5a1/Itga4/Spp1/Itgam/Col16a1/Itga1/Kdr | 15 |
| 2 weeks BCG/Nano-FP1 upregulated | R-MMU-913531 | Interferon Signaling | 0.0112837287137155 | Irf9/Plcg1/Ifngr1/Ifngr2/Ifnar2/Jak2/Flnb/Stat1/Usp18/Socs1/Socs3/Ifng/Camk2b | 13 |
| 2 weeks BCG/Nano-FP1 upregulated | R-MMU-166658 | Complement cascade | 0.0134387169417463 | C3/C2/Cd55/Cr2/Cd19/C1qa/C1qc/C1qb/C1s1/C3ar1/C1ra | 11 |
| 2 weeks BCG/Nano-FP1 upregulated | R-MMU-166786 | Creation of C4 and C2 activators | 0.0153981496828805 | C1qa/C1qc/C1qb/C1s1/C1ra | 5 |
| 2 weeks BCG/Nano-FP1 upregulated | R-MMU-373076 | Class A/1 (Rhodopsin-like receptors) | 0.0153981496828805 | Psap/Cxcl16/Adora2a/Gpr132/F2rl1/Gpr65/C3/Htr7/Nmb/Htr2b/Xcl1/P2ry1/Cxcl9/Ccr9/Npy/Lpar6/Cysltr2/Cxcl10/Hrh2/Ccl5/P2ry14/C3ar1/Ccr6/Cmklr1/Ccr8/Ptgir/Ccrl2/S1pr1/Gpr68/Cxcr5/F2r/Cxcr6/Cxcr3/Gpr18/P2ry10/Gpr183/Ptafr/Ece1/Ccr5 | 39 |
| 2 weeks BCG/Nano-FP1 upregulated | R-MMU-2029485 | Role of phospholipids in phagocytosis | 0.0157595409074064 | Cd3g/Cd247/Plcg1/Fcgr2b/Pik3ca/Prkce/Fcgr3 | 7 |
| 2 weeks BCG/Nano-FP1 upregulated | R-MMU-5673001 | RAF/MAP kinase cascade | 0.0159017411137085 | Vwf/Rasa4/Arrb1/Itgb3/Vcl/Psme1/Psmb8/Pdgfrb/Jak2/Pdgfa/Rasgrp1/Spred1/Dusp2/Src/Hgf/Psmd8/Lat/Il2rg/Rasa3/Dusp4/Dusp10/Il17rd/Rasal3/Actn2/Camk2b/Il2rb/Il3ra/Rasgrp3/Psme2b/Psme2/Psmb9/Flt3l | 32 |
| 2 weeks BCG/Nano-FP1 upregulated | R-MMU-5213460 | RIPK1-mediated regulated necrosis | 0.0190087611199937 | Fasl/Mlkl/Ripk3/Fas/Birc3/Tnfsf10 | 6 |
| 2 weeks BCG/Nano-FP1 upregulated | R-MMU-5218859 | Regulated Necrosis | 0.0190087611199937 | Fasl/Mlkl/Ripk3/Fas/Birc3/Tnfsf10 | 6 |
| 2 weeks BCG/Nano-FP1 upregulated | R-MMU-983695 | Antigen activates B Cell Receptor (BCR) leading to generation of second messengers | 0.0190087611199937 | Cd79a/Itpr1/Cd19/Pik3cd/Cd79b/Itpr3/Blnk | 7 |
| 2 weeks BCG/Nano-FP1 upregulated | R-MMU-5684996 | MAPK1/MAPK3 signaling | 0.0217039884128944 | Vwf/Rasa4/Arrb1/Itgb3/Vcl/Psme1/Psmb8/Pdgfrb/Jak2/Pdgfa/Rasgrp1/Spred1/Dusp2/Src/Hgf/Psmd8/Lat/Il2rg/Rasa3/Dusp4/Dusp10/Il17rd/Rasal3/Actn2/Camk2b/Il2rb/Il3ra/Rasgrp3/Psme2b/Psme2/Psmb9/Flt3l | 32 |
| 2 weeks BCG/Nano-FP1 upregulated | R-MMU-1474244 | Extracellular matrix organization | 0.0269809388074959 | Mmp11/Mmp14/Icam2/Itgb7/Col18a1/Vwf/Itgae/Ctsd/Adam19/Itga2/Sdc4/Timp2/P4ha1/Dcn/Sdc1/Itgb3/Ctsl/Ctsb/P3h3/Ltbp3/Pdgfa/Col3a1/Col5a1/Itga4/Spp1/Itgam/Bgn/Mmp2/Crtap/Ctss/Col16a1/Itga1/Mmp12/Kdr/Ceacam1 | 35 |
| 2 weeks BCG/Nano-FP1 upregulated | R-MMU-5683057 | MAPK family signaling cascades | 0.0271872795209448 | Vwf/Rasa4/Arrb1/Itgb3/Vcl/Psme1/Psmb8/Pdgfrb/Jak2/Pdgfa/Rasgrp1/Spred1/Dusp2/Src/Hgf/Psmd8/Lat/Il2rg/Rasa3/Dusp4/Cdc14a/Dusp10/Il17rd/Foxo1/Rasal3/Actn2/Camk2b/Il2rb/Il3ra/Rasgrp3/Psme2b/Psme2/Psmb9/Flt3l | 34 |
| 2 weeks BCG/Nano-FP1 upregulated | R-MMU-2029481 | FCGR activation | 0.0295980330455678 | Cd3g/Cd247/Fcgr2b/Src/Fcgr3 | 5 |
| 2 weeks BCG/Nano-FP1 upregulated | R-MMU-456926 | Thrombin signalling through proteinase activated receptors (PARs) | 0.0303922790718437 | Gna12/Arrb1/Src/Gnb4/Gng11/Gng12/Gng2/F2r | 8 |
| 2 weeks BCG/Nano-FP1 upregulated | R-MMU-2029480 | Fcgamma receptor (FCGR) dependent phagocytosis | 0.0350569801356187 | Cd3g/Cd247/Plcg1/Cyfip2/Myo10/Baiap2/Fcgr2b/Abi2/Src/Pik3ca/Limk1/Myo5a/Prkce/Fcgr3 | 14 |
| 2 weeks BCG/Nano-FP1 upregulated | R-MMU-2132295 | MHC class II antigen presentation | 0.0407210405942372 | Ctse/Ctsd/Kif3a/Kif3c/Lgmn/Ctsl/Ctsb/H2-Oa/Cd74/Lag3/Ctsh/H2-Aa/H2-DMb2/Ctss/H2-Ob/H2-Eb1/H2-Eb2/H2-Ab1 | 18 |
| 11 weeks BCG/Nano-FP1 downregulated | R-MMU-211945 | Phase I - Functionalization of compounds | 0.00436255412624731 | Cyp2a5/Aldh3a1/Cmbl/Fmo3/Cyp4b1/Cyp2b10/Fmo2/Cyp2f2/Adh7 | 9 |
| 11 weeks BCG/Nano-FP1 downregulated | R-MMU-1266738 | Developmental Biology | 0.00791476549001076 | Grb7/Akap5/Dpysl2/Krt18/Dpysl3/Col4a2/Krt80/Ppl/Adam22/Krt8/Tchh/Robo2/Dsp/Krt79/Sema3e | 15 |
| 11 weeks BCG/Nano-FP1 downregulated | R-MMU-6809371 | Formation of the cornified envelope | 0.00791476549001076 | Krt18/Krt80/Ppl/Krt8/Tchh/Dsp/Krt79 | 7 |
| 11 weeks BCG/Nano-FP1 downregulated | R-MMU-211859 | Biological oxidations | 0.0169963325723478 | Cyp2a5/Aldh3a1/Cmbl/Oplah/Fmo3/Cyp4b1/Cyp2b10/Fmo2/Cyp2f2/Adh7 | 10 |
| 11 weeks BCG/Nano-FP1 downregulated | R-MMU-6799198 | Complex I biogenesis | 0.0169963325723478 | ND1/ND2/ND4/ND5/ND6 | 5 |
| 11 weeks BCG/Nano-FP1 downregulated | R-MMU-211935 | Fatty acids | 0.0180006654031879 | Cyp2a5/Cyp4b1/Cyp2b10/Cyp2f2 | 4 |
| 11 weeks BCG/Nano-FP1 downregulated | R-MMU-611105 | Respiratory electron transport | 0.0180006654031879 | ND1/ND2/ND4/ND5/ND6 | 5 |
| 11 weeks BCG/Nano-FP1 downregulated | R-MMU-6805567 | Keratinization | 0.023179566809947 | Krt18/Krt80/Ppl/Krt8/Tchh/Dsp/Krt79 | 7 |
| 11 weeks BCG/Nano-FP1 upregulated | R-MMU-198933 | Immunoregulatory interactions between a Lymphoid and a non-Lymphoid cell | 2.33365546075102e-11 | Itgb7/Cd3g/Cd247/H2-M2/Cd40/Cd96/Cd200/Klrk1/Pianp/Cd3e/Cd3d/Cd226/H2-Q4/Slamf7/Cd8b1/Cd8a/H2-Q7/H2-K1/H2-T23/Treml2/H2-Q6 | 21 |
| 11 weeks BCG/Nano-FP1 upregulated | R-MMU-202430 | Translocation of ZAP-70 to Immunological synapse | 1.41374120502648e-08 | Lck/Cd3g/Cd247/Cd4/Zap70/Ptpn22/Cd3e/Cd3d | 8 |
| 11 weeks BCG/Nano-FP1 upregulated | R-MMU-202433 | Generation of second messenger molecules | 1.41374120502648e-08 | Lck/Cd3g/Cd247/Itk/Fyb/Cd4/Zap70/Lat/Cd3e/Cd3d | 10 |
| 11 weeks BCG/Nano-FP1 upregulated | R-MMU-202427 | Phosphorylation of CD3 and TCR zeta chains | 2.48561064483118e-06 | Lck/Cd3g/Cd247/Cd4/Ptpn22/Cd3e/Cd3d | 7 |
| 11 weeks BCG/Nano-FP1 upregulated | R-MMU-388841 | Costimulation by the CD28 family | 3.90728820280153e-05 | Lck/Cd3g/Cd247/Akt3/Cd4/Icos/Ctla4/Cd28/Cd3e/Cd3d/Trib3 | 11 |
| 11 weeks BCG/Nano-FP1 upregulated | R-MMU-389948 | PD-1 signaling | 0.000147182290870139 | Lck/Cd3g/Cd247/Cd4/Cd3e/Cd3d | 6 |
| 11 weeks BCG/Nano-FP1 upregulated | R-MMU-1236974 | ER-Phagosome pathway | 0.000466641028230125 | H2-M2/H2-Q4/Tap1/H2-Q7/H2-K1/H2-T23/H2-Q6 | 7 |
| 11 weeks BCG/Nano-FP1 upregulated | R-MMU-1236977 | Endosomal/Vacuolar pathway | 0.000490631840509774 | H2-M2/H2-Q4/H2-Q7/H2-K1/H2-T23/H2-Q6 | 6 |
| 11 weeks BCG/Nano-FP1 upregulated | R-MMU-202403 | TCR signaling | 0.000580422704545048 | Lck/Cd3g/Cd247/Itk/Fyb/Cd4/Zap70/Ptpn22/Lat/Trat1/Cd3e/Cd3d | 12 |
| 11 weeks BCG/Nano-FP1 upregulated | R-MMU-983170 | Antigen Presentation: Folding, assembly and peptide loading of class I MHC | 0.00150180879873848 | H2-M2/H2-Q4/Tap1/H2-Q7/H2-K1/H2-T23/H2-Q6 | 7 |
| 11 weeks BCG/Nano-FP1 upregulated | R-MMU-76002 | Platelet activation, signaling and aggregation | 0.00602673780605456 | Lck/Arrb1/Igf1/Srgn/Vcl/Ctsw/Cd63/Pdgfa/Rasgrp1/Lat/Rasgrp2/Lgals3bp/Gng12/Orm1/Itpr3/Cd109/Prkcb/Serpina3f | 18 |
| 11 weeks BCG/Nano-FP1 upregulated | R-MMU-380108 | Chemokine receptors bind chemokines | 0.0125658195882285 | Cxcl16/Ccl5/Ccr6/Ccrl2/Ccr4/Cxcr6/Cxcr3 | 7 |
| 11 weeks BCG/Nano-FP1 upregulated | R-MMU-76005 | Response to elevated platelet cytosolic Ca2+ | 0.0333504416200571 | Igf1/Srgn/Vcl/Ctsw/Cd63/Pdgfa/Lgals3bp/Orm1/Cd109/Prkcb/Serpina3f | 11 |
| 14 weeks BCG/Nano-FP1 upregulated | R-MMU-198933 | Immunoregulatory interactions between a Lymphoid and a non-Lymphoid cell | 1.37896139275533e-17 | Itgb7/Cd3g/Sh2d1a/Cd247/H2-M3/H2-M2/Cd40/Cd96/Cd200/Trem2/C3/Klrk1/Pianp/Cd19/Cd40lg/Crtam/Cd3e/Cd3d/Cd226/H2-Q4/Slamf7/Cd8b1/Cd8a/H2-Q7/B2m/H2-K1/H2-T23/Treml2/H2-Q6/H2-D1/Cd200r2 | 31 |
| 14 weeks BCG/Nano-FP1 upregulated | R-MMU-202430 | Translocation of ZAP-70 to Immunological synapse | 2.79595549862848e-15 | Lck/Cd3g/Cd247/Cd4/Zap70/Ptpn22/Cd3e/Cd3d/H2-Aa/H2-Eb1/H2-Eb2/H2-Ab1 | 12 |
| 14 weeks BCG/Nano-FP1 upregulated | R-MMU-202433 | Generation of second messenger molecules | 3.50355153268732e-14 | Lck/Cd3g/Cd247/Plcg1/Itk/Fyb/Cd4/Zap70/Lat/Cd3e/Cd3d/H2-Aa/H2-Eb1/H2-Eb2/H2-Ab1 | 15 |
| 14 weeks BCG/Nano-FP1 upregulated | R-MMU-388841 | Costimulation by the CD28 family | 7.37085656200728e-13 | Lck/Cd3g/Cd247/Cd274/Akt3/Fyn/Cd86/Cd4/Icos/Ctla4/Cd28/Pdcd1/Src/Cd3e/Cd3d/Trib3/H2-Aa/Tnfrsf14/H2-Eb1/H2-Eb2/H2-Ab1 | 21 |
| 14 weeks BCG/Nano-FP1 upregulated | R-MMU-389948 | PD-1 signaling | 1.77043507453682e-12 | Lck/Cd3g/Cd247/Cd274/Cd4/Pdcd1/Cd3e/Cd3d/H2-Aa/H2-Eb1/H2-Eb2/H2-Ab1 | 12 |
| 14 weeks BCG/Nano-FP1 upregulated | R-MMU-202427 | Phosphorylation of CD3 and TCR zeta chains | 8.20820946464317e-12 | Lck/Cd3g/Cd247/Cd4/Ptpn22/Cd3e/Cd3d/H2-Aa/H2-Eb1/H2-Eb2/H2-Ab1 | 11 |
| 14 weeks BCG/Nano-FP1 upregulated | R-MMU-202403 | TCR signaling | 4.02265583081678e-11 | Lck/Cd3g/Cd247/Plcg1/Itk/Fyb/Psme1/Cd4/Psmb8/Zap70/Prkcq/Ptpn22/Lat/Trat1/Psmb10/Cd3e/Cd3d/Malt1/H2-Aa/H2-Eb1/H2-Eb2/H2-Ab1/Psme2/Psmb9 | 24 |
| 14 weeks BCG/Nano-FP1 upregulated | R-MMU-1236974 | ER-Phagosome pathway | 2.00599058721812e-08 | H2-M3/H2-M2/Tapbp/Tap2/H2-Q4/Tap1/H2-Q7/B2m/H2-K1/H2-T23/H2-Q6/H2-D1 | 12 |
| 14 weeks BCG/Nano-FP1 upregulated | R-MMU-202424 | Downstream TCR signaling | 1.78241697483849e-07 | Lck/Cd3g/Cd247/Psme1/Cd4/Psmb8/Prkcq/Trat1/Psmb10/Cd3e/Cd3d/Malt1/H2-Aa/H2-Eb1/H2-Eb2/H2-Ab1/Psme2/Psmb9 | 18 |
| 14 weeks BCG/Nano-FP1 upregulated | R-MMU-983170 | Antigen Presentation: Folding, assembly and peptide loading of class I MHC | 3.03389543487509e-07 | H2-M3/H2-M2/Tapbp/Tap2/H2-Q4/Tap1/H2-Q7/B2m/H2-K1/H2-T23/H2-Q6/H2-D1 | 12 |
| 14 weeks BCG/Nano-FP1 upregulated | R-MMU-1280215 | Cytokine Signaling in Immune system | 3.17509610710986e-07 | Lck/Il12rb1/Irf9/Ebi3/Il7r/Tnfrsf13b/Plcg1/Cd40/Fyn/Il9r/Sdc1/Tnfsf11/Psme1/Cd4/Psmb8/Ltb/Lta/Tnfrsf25/Flnb/Il18r1/Stat1/Tnfsf8/Il21r/Cd40lg/Il2rg/Dusp4/Jak3/Psmb10/Crlf2/Pik3cd/Tnfrsf14/Jun/Ifng/Camk2b/Stat4/Il2rb/Il18bp/Il11ra1/Psme2/Psmb9/Flt3l | 41 |
| 14 weeks BCG/Nano-FP1 upregulated | R-MMU-1236977 | Endosomal/Vacuolar pathway | 1.03100965740453e-06 | H2-M3/H2-M2/H2-Q4/H2-Q7/B2m/H2-K1/H2-T23/H2-Q6/H2-D1 | 9 |
| 14 weeks BCG/Nano-FP1 upregulated | R-MMU-1236975 | Antigen processing-Cross presentation | 1.03828169442131e-06 | H2-M3/H2-M2/Psme1/Tapbp/Psmb8/Tap2/Psmb10/H2-Q4/Tap1/H2-Q7/B2m/H2-K1/H2-T23/H2-Q6/H2-D1/Psme2/Psmb9 | 17 |
| 14 weeks BCG/Nano-FP1 upregulated | R-MMU-166663 | Initial triggering of complement | 3.65804610305895e-06 | C3/C2/C1qa/C1qc/C1qb/C1s1/C1ra/C4b | 8 |
| 14 weeks BCG/Nano-FP1 upregulated | R-MMU-983705 | Signaling by the B Cell Receptor (BCR) | 1.06746750209556e-05 | Cd79a/Psme1/Psmb8/Rasgrp1/Nfatc2/Itpr1/Cd19/Psmb10/Malt1/Pik3cd/Cd79b/Itpr3/Prkcb/Blnk/Rasgrp3/Psme2/Psmb9 | 17 |
| 14 weeks BCG/Nano-FP1 upregulated | R-MMU-2424491 | DAP12 signaling | 4.68156100232058e-05 | Lck/Plcg1/Fyn/Trem2/Klrk1/Klrd1/Klrc1/Lat/B2m | 9 |
| 14 weeks BCG/Nano-FP1 upregulated | R-MMU-977606 | Regulation of Complement cascade | 0.000115650041319586 | C3/C2/Cr2/Cd19/C1qa/C1qc/C1qb/C1s1/C1ra/C4b | 10 |
| 14 weeks BCG/Nano-FP1 upregulated | R-MMU-166658 | Complement cascade | 0.00039527184611729 | C3/C2/Cr2/Cd19/C1qa/C1qc/C1qb/C1s1/C1ra/C4b | 10 |
| 14 weeks BCG/Nano-FP1 upregulated | R-MMU-5668541 | TNFR2 non-canonical NF-kB pathway | 0.00054247826898148 | Tnfrsf13b/Cd40/Tnfsf11/Psme1/Psmb8/Ltb/Lta/Tnfrsf25/Tnfsf8/Cd40lg/Psmb10/Tnfrsf14/Psme2/Psmb9 | 14 |
| 14 weeks BCG/Nano-FP1 upregulated | R-MMU-983695 | Antigen activates B Cell Receptor (BCR) leading to generation of second messengers | 0.00054247826898148 | Cd79a/Itpr1/Cd19/Pik3cd/Cd79b/Itpr3/Blnk | 7 |
| 14 weeks BCG/Nano-FP1 upregulated | R-MMU-9020958 | Interleukin-21 signaling | 0.00054247826898148 | Stat1/Il21r/Il2rg/Jak3/Stat4 | 5 |
| 14 weeks BCG/Nano-FP1 upregulated | R-MMU-2172127 | DAP12 interactions | 0.000548445106357809 | Lck/Plcg1/Fyn/Trem2/Klrk1/Klrd1/Klrc1/Lat/B2m | 9 |
| 14 weeks BCG/Nano-FP1 upregulated | R-MMU-451927 | Interleukin-2 family signaling | 0.000548445106357809 | Lck/Il9r/Stat1/Il21r/Il2rg/Jak3/Pik3cd/Stat4/Il2rb | 9 |
| 14 weeks BCG/Nano-FP1 upregulated | R-MMU-449147 | Signaling by Interleukins | 0.000731382671047637 | Lck/Il12rb1/Ebi3/Il7r/Fyn/Il9r/Sdc1/Psme1/Cd4/Psmb8/Il18r1/Stat1/Il21r/Il2rg/Dusp4/Jak3/Psmb10/Crlf2/Pik3cd/Jun/Stat4/Il2rb/Il18bp/Il11ra1/Psme2/Psmb9/Flt3l | 27 |
| 14 weeks BCG/Nano-FP1 upregulated | R-MMU-166786 | Creation of C4 and C2 activators | 0.000809161108788982 | C1qa/C1qc/C1qb/C1s1/C1ra | 5 |
| 14 weeks BCG/Nano-FP1 upregulated | R-MMU-389513 | CTLA4 inhibitory signaling | 0.00256551966618957 | Lck/Akt3/Fyn/Cd86/Ctla4/Src | 6 |
| 14 weeks BCG/Nano-FP1 upregulated | R-MMU-380108 | Chemokine receptors bind chemokines | 0.00289005644690548 | Cxcl16/Ccl1/Cxcl9/Ccl5/Ccr6/Cxcr5/Cxcr6/Cxcr3/Ccr5 | 9 |
| 14 weeks BCG/Nano-FP1 upregulated | R-MMU-389357 | CD28 dependent PI3K/Akt signaling | 0.00317058558620017 | Lck/Akt3/Fyn/Cd86/Cd28/Trib3 | 6 |
| 14 weeks BCG/Nano-FP1 upregulated | R-MMU-5673001 | RAF/MAP kinase cascade | 0.00339256958846455 | Rasa4/Met/Arrb1/Fyn/Psme1/Psmb8/Rasgrp1/Dusp2/Src/Lat/Il2rg/Dusp4/Jak3/Psmb10/Rasal3/Dlg2/Camk2b/Il2rb/Rasgrp3/Psme2/Psmb9/Flt3l | 22 |
| 14 weeks BCG/Nano-FP1 upregulated | R-MMU-389356 | CD28 co-stimulation | 0.00445901219362937 | Lck/Akt3/Fyn/Cd86/Cd28/Src/Trib3 | 7 |
| 14 weeks BCG/Nano-FP1 upregulated | R-MMU-5684996 | MAPK1/MAPK3 signaling | 0.00445901219362937 | Rasa4/Met/Arrb1/Fyn/Psme1/Psmb8/Rasgrp1/Dusp2/Src/Lat/Il2rg/Dusp4/Jak3/Psmb10/Rasal3/Dlg2/Camk2b/Il2rb/Rasgrp3/Psme2/Psmb9/Flt3l | 22 |
| 14 weeks BCG/Nano-FP1 upregulated | R-MMU-199418 | Negative regulation of the PI3K/AKT network | 0.00445901219362937 | Lck/Met/Akt3/Esr1/Fyn/Cd86/Icos/Cd28/Src/Cd19/Trat1/Trib3/Pik3cd | 13 |
| 14 weeks BCG/Nano-FP1 upregulated | R-MMU-5676594 | TNF receptor superfamily (TNFSF) members mediating non-canonical NF-kB pathway | 0.00678301797997073 | Cd40/Tnfsf11/Ltb/Lta/Cd40lg | 5 |
| 14 weeks BCG/Nano-FP1 upregulated | R-MMU-5669034 | TNFs bind their physiological receptors | 0.00707374053692195 | Tnfrsf13b/Tnfsf11/Lta/Tnfrsf25/Tnfsf8/Tnfrsf14 | 6 |
| 14 weeks BCG/Nano-FP1 upregulated | R-MMU-202733 | Cell surface interactions at the vascular wall | 0.00765811891469741 | Lck/Plcg1/Fyn/Sdc1/Cd74/Procr/Src/Itgam/Mif/Gpc1/Slc7a5/Spn/Jchain | 13 |
| 14 weeks BCG/Nano-FP1 upregulated | R-MMU-76002 | Platelet activation, signaling and aggregation | 0.00883736105334193 | Lck/Arrb1/Fyn/Ctsw/Cd63/Prkcq/Rasgrp1/Src/Gnb4/Ecm1/Pdpn/Itpr1/Lat/Lgals3bp/Gng12/Endod1/Orm1/Itpr3/Gng2/F2r/Prkcb/Serpina3f | 22 |
| 14 weeks BCG/Nano-FP1 upregulated | R-MMU-210990 | PECAM1 interactions | 0.010046779259715 | Lck/Plcg1/Fyn/Src | 4 |
| 14 weeks BCG/Nano-FP1 upregulated | R-MMU-2454202 | Fc epsilon receptor (FCERI) signaling | 0.0106976512806553 | Plcg1/Itk/Psme1/Psmb8/Prkcq/Nfatc2/Lat/Psmb10/Malt1/Lat2/Jun/Psme2/Psmb9 | 13 |
| 14 weeks BCG/Nano-FP1 upregulated | R-MMU-1168372 | Downstream signaling events of B Cell Receptor (BCR) | 0.0113799939285004 | Psme1/Psmb8/Rasgrp1/Nfatc2/Psmb10/Malt1/Prkcb/Rasgrp3/Psme2/Psmb9 | 10 |
| 14 weeks BCG/Nano-FP1 upregulated | R-MMU-5683057 | MAPK family signaling cascades | 0.0127581943623684 | Rasa4/Met/Arrb1/Fyn/Psme1/Psmb8/Rasgrp1/Dusp2/Src/Lat/Il2rg/Dusp4/Jak3/Psmb10/Rasal3/Dlg2/Camk2b/Il2rb/Rasgrp3/Psme2/Psmb9/Flt3l | 22 |
| 14 weeks BCG/Nano-FP1 upregulated | R-MMU-389359 | CD28 dependent Vav1 pathway | 0.0127581943623684 | Lck/Fyn/Cd86/Cd28 | 4 |
| 14 weeks BCG/Nano-FP1 upregulated | R-MMU-9020558 | Interleukin-2 signaling | 0.0127581943623684 | Lck/Il2rg/Jak3/Il2rb | 4 |
| 14 weeks BCG/Nano-FP1 upregulated | R-MMU-9006925 | Intracellular signaling by second messengers | 0.0127581943623684 | Lck/Met/Akt3/Esr1/Fyn/Psme1/Adcy4/Cd86/Psmb8/Icos/Cd28/Src/Cd19/Trat1/Psmb10/Trib3/Pml/Camk4/Pik3cd/Psme2/Psmb9 | 21 |
| 14 weeks BCG/Nano-FP1 upregulated | R-MMU-2029481 | FCGR activation | 0.0172195416006737 | Cd3g/Cd247/Fyn/Src | 4 |
| 14 weeks BCG/Nano-FP1 upregulated | R-MMU-456926 | Thrombin signalling through proteinase activated receptors (PARs) | 0.0173181973093398 | Arrb1/Src/Gnb4/Gng12/Gng2/F2r | 6 |
| 14 weeks BCG/Nano-FP1 upregulated | R-MMU-351202 | Metabolism of polyamines | 0.01745347495817 | Slc6a8/Arg1/Psme1/Psmb8/Paox/Psmb10/Nags/Ass1/Psme2/Psmb9 | 10 |
| 14 weeks BCG/Nano-FP1 upregulated | R-MMU-1257604 | PIP3 activates AKT signaling | 0.0178541553931323 | Lck/Met/Akt3/Esr1/Fyn/Psme1/Cd86/Psmb8/Icos/Cd28/Src/Cd19/Trat1/Psmb10/Trib3/Pml/Pik3cd/Psme2/Psmb9 | 19 |
| 14 weeks BCG/Nano-FP1 upregulated | R-MMU-6811558 | PI5P, PP2A and IER3 Regulate PI3K/AKT Signaling | 0.0206640071261029 | Lck/Met/Esr1/Fyn/Cd86/Icos/Cd28/Src/Cd19/Trat1/Pik3cd | 11 |
| 14 weeks BCG/Nano-FP1 upregulated | R-MMU-373076 | Class A/1 (Rhodopsin-like receptors) | 0.0282653038189929 | Cxcl16/Ccl1/Gpr132/C3/Htr2b/Cxcl9/Npy/Lpar6/Cysltr2/Ccl5/Ccr6/Cmklr1/Ptgir/S1pr4/S1pr1/Gpr68/Cxcr5/F2r/Cxcr6/Cxcr3/Gpr18/P2ry10/Ccr5 | 23 |
| 14 weeks BCG/Nano-FP1 upregulated | R-MMU-418346 | Platelet homeostasis | 0.0448056678625553 | Atp2a3/Nos2/Atp2b4/Gnb4/Itpr1/Gng12/Itpr3/Gng2/Ptgir | 9 |

ID= Reactome pathway ID. P.adjust = adjusted p-value; indicates the significance of the enrichment. Count = number of genes from the submitted list found in the pathway.

**Table S4**. Reactome Pathway Enrichment results obtained from Lung parenchyma analysis

| Cluster | ID | Description | p.adjust | geneID | Count |
| --- | --- | --- | --- | --- | --- |
| 2 weeks BCG/Nano-FP1 upregulated | R-MMU-191273 | Cholesterol biosynthesis | 6.55178171809532e-10 | Cyp51/Mvd/Fdft1/Sqle/Acat2/Hsd17b7/Pmvk/Msmo1/Sc5d/Lss/Dhcr24/Mvk/Idi1/Dhcr7/Fdps | 15 |
| 2 weeks BCG/Nano-FP1 upregulated | R-MMU-389948 | PD-1 signaling | 4.66930646671055e-08 | Lck/Cd3g/Cd274/Pdcd1lg2/Cd4/Pdcd1/Cd3e/H2-Aa/H2-Eb1/H2-Eb2/H2-Ab1 | 11 |
| 2 weeks BCG/Nano-FP1 upregulated | R-MMU-202430 | Translocation of ZAP-70 to Immunological synapse | 4.91735933356183e-07 | Lck/Cd3g/Cd4/Zap70/Cd3e/H2-Aa/H2-Eb1/H2-Eb2/H2-Ab1 | 9 |
| 2 weeks BCG/Nano-FP1 upregulated | R-MMU-388841 | Costimulation by the CD28 family | 2.71943464775286e-06 | Lck/Cd3g/Cd274/Pdcd1lg2/Akt3/Cd86/Cd4/Icos/Ctla4/Pdcd1/Src/Cd3e/Trib3/H2-Aa/Tnfrsf14/H2-Eb1/H2-Eb2/H2-Ab1 | 18 |
| 2 weeks BCG/Nano-FP1 upregulated | R-MMU-198933 | Immunoregulatory interactions between a Lymphoid and a non-Lymphoid cell | 3.02421113907101e-06 | Cd3g/H2-M3/H2-M2/Cd40/Trem2/C3/Pianp/Cd40lg/Cd3e/Cd226/Cd300a/H2-Q4/Icam1/Slamf7/Cd8a/H2-Q7/B2m/H2-K1/Cd200r4/H2-T23/H2-Q6/H2-D1/Cd200r2 | 23 |
| 2 weeks BCG/Nano-FP1 upregulated | R-MMU-1236974 | ER-Phagosome pathway | 5.59902898535852e-06 | H2-M3/H2-M2/Tapbp/Tap2/H2-Q4/Tap1/H2-Q7/B2m/H2-K1/H2-T23/H2-Q6/H2-D1 | 12 |
| 2 weeks BCG/Nano-FP1 upregulated | R-MMU-1236975 | Antigen processing-Cross presentation | 1.4983507193288e-05 | Cyba/Fcgr1/H2-M3/H2-M2/Itgb5/Tapbp/Psmb8/Tap2/Psmd8/Psmb10/H2-Q4/Tap1/H2-Q7/B2m/H2-K1/H2-T23/H2-Q6/H2-D1/Psme2/Psmb9 | 20 |
| 2 weeks BCG/Nano-FP1 upregulated | R-MMU-1280215 | Cytokine Signaling in Immune system | 2.14241515758191e-05 | Lck/Il12rb1/Relb/Ebi3/Il12b/Csf1/Cd40/Prkcd/Tnfsf11/Ifngr2/Il10rb/Cd4/Psmb8/Lta/Csf1r/Tnfrsf25/Flnb/Il17a/Il18r1/Stat1/Il21/Tnfsf8/Tnfrsf1b/Tnfrsf4/Usp18/Psmd8/Il21r/Cd40lg/Il2rg/Tnfsf13b/Dusp4/Jak3/Psmb10/Mapkapk3/Uba7/Crlf2/Isg15/Stat2/Stx3/Tnfrsf18/Tnfrsf14/Il17re/Ifng/Il2rb/Il3ra/Hist1h3a/Il18bp/Il11ra1/Psme2/Psmb9/Tnfsf12 | 51 |
| 2 weeks BCG/Nano-FP1 upregulated | R-MMU-202433 | Generation of second messenger molecules | 2.8558779011736e-05 | Lck/Cd3g/Cd4/Zap70/Lat/Cd3e/H2-Aa/H2-Eb1/H2-Eb2/H2-Ab1 | 10 |
| 2 weeks BCG/Nano-FP1 upregulated | R-MMU-202427 | Phosphorylation of CD3 and TCR zeta chains | 2.8558779011736e-05 | Lck/Cd3g/Cd4/Cd3e/H2-Aa/H2-Eb1/H2-Eb2/H2-Ab1 | 8 |
| 2 weeks BCG/Nano-FP1 upregulated | R-MMU-983170 | Antigen Presentation: Folding, assembly and peptide loading of class I MHC | 4.96958053015077e-05 | H2-M3/H2-M2/Tapbp/Tap2/H2-Q4/Tap1/H2-Q7/B2m/H2-K1/H2-T23/H2-Q6/H2-D1 | 12 |
| 2 weeks BCG/Nano-FP1 upregulated | R-MMU-1236977 | Endosomal/Vacuolar pathway | 6.03516620910263e-05 | H2-M3/H2-M2/H2-Q4/H2-Q7/B2m/H2-K1/H2-T23/H2-Q6/H2-D1 | 9 |
| 2 weeks BCG/Nano-FP1 upregulated | R-MMU-5669034 | TNFs bind their physiological receptors | 0.000745127243818421 | Tnfsf11/Lta/Tnfrsf25/Tnfsf8/Tnfrsf1b/Tnfrsf4/Tnfsf13b/Tnfrsf18/Tnfrsf14 | 9 |
| 2 weeks BCG/Nano-FP1 upregulated | R-MMU-5668541 | TNFR2 non-canonical NF-kB pathway | 0.000824581127752583 | Relb/Cd40/Tnfsf11/Psmb8/Lta/Tnfrsf25/Tnfsf8/Tnfrsf1b/Tnfrsf4/Psmd8/Cd40lg/Tnfsf13b/Psmb10/Tnfrsf18/Tnfrsf14/Psme2/Psmb9/Tnfsf12 | 18 |
| 2 weeks BCG/Nano-FP1 upregulated | R-MMU-1660662 | Glycosphingolipid metabolism | 0.000861170232040473 | Gm2a/Psap/Ctsa/Galc/Hexb/Asah2/Hexa/Gba/Neu3/B3galnt1/Glb1 | 11 |
| 2 weeks BCG/Nano-FP1 upregulated | R-MMU-2029485 | Role of phospholipids in phagocytosis | 0.00122967439463775 | Cd3g/Pld3/Fcgr1/Prkcd/Fcgr2b/Pld4/Fcgr4/Fcgr3 | 8 |
| 2 weeks BCG/Nano-FP1 upregulated | R-MMU-166663 | Initial triggering of complement | 0.00146860723940256 | C3/C2/C1qa/C1qc/C1qb/C1s1/C1ra | 7 |
| 2 weeks BCG/Nano-FP1 upregulated | R-MMU-2173782 | Binding and Uptake of Ligands by Scavenger Receptors | 0.00157502989505755 | Apoe/Msr1/Stab1/Apol7c/Apol10b/Apol9a/Jchain/Apol9b/Apol7b/Apol7e | 10 |
| 2 weeks BCG/Nano-FP1 upregulated | R-MMU-380108 | Chemokine receptors bind chemokines | 0.00157502989505755 | Cxcl16/Ccl1/Xcl1/Cxcl9/Cxcl10/Ccr8/Ccrl2/Ccr4/Cxcr6/Cxcr3/Xcr1/Ccr5 | 12 |
| 2 weeks BCG/Nano-FP1 upregulated | R-MMU-428157 | Sphingolipid metabolism | 0.00161985715131902 | Gm2a/Psap/Ctsa/Sgpl1/Galc/Hexb/Asah2/Hexa/Gba/Plpp3/Acer3/Neu3/B3galnt1/Glb1/Plpp2/Sphk1 | 16 |
| 2 weeks BCG/Nano-FP1 upregulated | R-MMU-2029481 | FCGR activation | 0.00206330948106536 | Cd3g/Fcgr1/Fcgr2b/Src/Fcgr4/Fcgr3 | 6 |
| 2 weeks BCG/Nano-FP1 upregulated | R-MMU-8957322 | Metabolism of steroids | 0.0022534840756127 | Cyp51/Stard3nl/Mvd/Abcc3/Lgmn/Fdft1/Sqle/Acat2/Stard4/Hsd17b7/Pmvk/Osbpl3/Msmo1/Sc5d/Lss/Dhcr24/Tspo/Mvk/Idi1/Dhcr7/Fdps | 21 |
| 2 weeks BCG/Nano-FP1 upregulated | R-MMU-381426 | Regulation of Insulin-like Growth Factor (IGF) transport and uptake by Insulin-like Growth Factor Binding Proteins (IGFBPs) | 0.00260803445076458 | Apoe/Csf1/Igf1/Fstl1/C3/Fam20c/Lamc1/Fbn1/Cst3/Spp1/Mfge8/Nucb1/Mmp2/Apol7c/Penk/Apol10b/Apol9a/Lgals1/Apol9b/Apol7b/Apol7e | 21 |
| 2 weeks BCG/Nano-FP1 upregulated | R-MMU-456926 | Thrombin signalling through proteinase activated receptors (PARs) | 0.00260803445076458 | Gna12/Arrb1/F2rl2/Src/Gnb4/Gna15/Gng12/Gng2/F2r | 9 |
| 2 weeks BCG/Nano-FP1 upregulated | R-MMU-8957275 | Post-translational protein phosphorylation | 0.0034747035203146 | Apoe/Csf1/Fstl1/C3/Fam20c/Lamc1/Fbn1/Cst3/Spp1/Mfge8/Nucb1/Apol7c/Penk/Apol10b/Apol9a/Lgals1/Apol9b/Apol7b/Apol7e | 19 |
| 2 weeks BCG/Nano-FP1 upregulated | R-MMU-9020958 | Interleukin-21 signaling | 0.00454431742947394 | Stat1/Il21/Il21r/Il2rg/Jak3 | 5 |
| 2 weeks BCG/Nano-FP1 upregulated | R-MMU-2024096 | HS-GAG degradation | 0.00620763543925722 | Naglu/Sgsh/Sdc3/Idua/Gpc1/Hpse/Glb1 | 7 |
| 2 weeks BCG/Nano-FP1 upregulated | R-MMU-166786 | Creation of C4 and C2 activators | 0.00725546969738654 | C1qa/C1qc/C1qb/C1s1/C1ra | 5 |
| 2 weeks BCG/Nano-FP1 upregulated | R-MMU-76002 | Platelet activation, signaling and aggregation | 0.00725546969738654 | Gna12/Lck/Pdgfb/Vwf/Psap/Arrb1/Igf1/F2rl2/Prkcd/Ctsw/Vegfb/Cd63/Pdgfa/Cyb5r1/Src/Gnb4/P2ry1/Ecm1/Pdpn/Hgf/Lat/Lgals3bp/Gna15/Gng12/Endod1/Orm1/Gng2/Lhfpl2/F2r/Actn2/Serpina3f | 31 |
| 2 weeks BCG/Nano-FP1 upregulated | R-MMU-1679131 | Trafficking and processing of endosomal TLR | 0.00736878697433925 | Lgmn/Ctsl/Ctsb/Tlr3/Unc93b1/Ctss/Tlr9 | 7 |
| 2 weeks BCG/Nano-FP1 upregulated | R-MMU-2168880 | Scavenging of heme from plasma | 0.00736878697433925 | Apol7c/Apol10b/Apol9a/Jchain/Apol9b/Apol7b/Apol7e | 7 |
| 2 weeks BCG/Nano-FP1 upregulated | R-MMU-917937 | Iron uptake and transport | 0.0076775209038551 | Tcirg1/Hmox1/Atp6v0a1/Atp6v1c1/Slc11a2/Atp6v0c/Steap3/Atp6v0b/Ftl1/Atp6v1a/LOC100862446 | 11 |
| 2 weeks BCG/Nano-FP1 upregulated | R-MMU-5676594 | TNF receptor superfamily (TNFSF) members mediating non-canonical NF-kB pathway | 0.0076775209038551 | Cd40/Tnfsf11/Lta/Cd40lg/Tnfsf13b/Tnfsf12 | 6 |
| 2 weeks BCG/Nano-FP1 upregulated | R-MMU-1222556 | ROS, RNS production in phagocytes | 0.0110839469887917 | Tcirg1/Cyba/Atp6v0a1/Atp6v1c1/Atp6v0c/Slc11a1/Atp6v0b/Atp6v1a | 8 |
| 2 weeks BCG/Nano-FP1 upregulated | R-MMU-2132295 | MHC class II antigen presentation | 0.0172899656124693 | Klc4/Ctsd/Ctsa/Kif3a/Lgmn/Ctsl/Ctsb/Cd74/Lag3/Ctsc/Ctsh/H2-Aa/Ctss/H2-Eb1/H2-Eb2/H2-Ab1/H2-DMb1 | 17 |
| 2 weeks BCG/Nano-FP1 upregulated | R-MMU-446219 | Synthesis of substrates in N-glycan biosythesis | 0.0175651047045074 | Mvd/St6galnac1/Ctsa/Fuom/Neu3/Amdhd2/Glb1/Slc17a5/St6galnac2/Dolk/St6galnac4 | 11 |
| 2 weeks BCG/Nano-FP1 upregulated | R-MMU-1630316 | Glycosaminoglycan metabolism | 0.0180090657794369 | Naglu/Sgsh/Hexb/Papss2/Hexa/Sdc3/B3gnt3/Chst2/Idua/Gpc1/Chst11/Gns/Hpse/Chst12/Glb1/B4galt6/Chst14 | 17 |
| 2 weeks BCG/Nano-FP1 upregulated | R-MMU-202403 | TCR signaling | 0.0235016196528961 | Lck/Cd3g/Cd4/Psmb8/Zap70/Psmd8/Lat/Psmb10/Cd3e/H2-Aa/H2-Eb1/H2-Eb2/H2-Ab1/Psme2/Psmb9 | 15 |
| 2 weeks BCG/Nano-FP1 upregulated | R-MMU-202424 | Downstream TCR signaling | 0.0332020528141222 | Lck/Cd3g/Cd4/Psmb8/Psmd8/Psmb10/Cd3e/H2-Aa/H2-Eb1/H2-Eb2/H2-Ab1/Psme2/Psmb9 | 13 |
| 2 weeks BCG/Nano-FP1 upregulated | R-MMU-917977 | Transferrin endocytosis and recycling | 0.0391962835996935 | Tcirg1/Atp6v0a1/Atp6v1c1/Atp6v0c/Steap3/Atp6v0b/Atp6v1a | 7 |
| 2 weeks BCG/Nano-FP1 upregulated | R-MMU-449147 | Signaling by Interleukins | 0.0391962835996935 | Lck/Il12rb1/Ebi3/Il12b/Csf1/Il10rb/Cd4/Psmb8/Csf1r/Il17a/Il18r1/Stat1/Il21/Psmd8/Il21r/Il2rg/Dusp4/Jak3/Psmb10/Mapkapk3/Crlf2/Stat2/Stx3/Il17re/Il2rb/Il3ra/Hist1h3a/Il18bp/Il11ra1/Psme2/Psmb9 | 31 |
| 2 weeks BCG/Nano-FP1 upregulated | R-MMU-71387 | Metabolism of carbohydrates | 0.0391962835996935 | Hk2/Naglu/Sgsh/Man2b1/Pgam1/Aldoc/Gyg/Pfkl/Hexb/Papss2/Got1/Hexa/Gaa/Sdc3/Hk3/Pfkfb3/B3gnt3/Slc37a2/Chst2/Idua/Gpc1/Chst11/Gns/Hpse/Chst12/Glb1/B4galt6/Chst14 | 28 |
| 2 weeks BCG/Nano-FP1 upregulated | R-MMU-1638074 | Keratan sulfate/keratin metabolism | 0.044230948382363 | Hexb/Hexa/B3gnt3/Chst2/Gns/Glb1/B4galt6 | 7 |
| 2 weeks BCG/Nano-FP1 upregulated | R-MMU-4085001 | Sialic acid metabolism | 0.044230948382363 | St6galnac1/Ctsa/Neu3/Glb1/Slc17a5/St6galnac2/St6galnac4 | 7 |
| 2 weeks BCG/Nano-FP1 upregulated | R-MMU-9006934 | Signaling by Receptor Tyrosine Kinases | 0.0452152908152581 | Lck/Pdgfb/Tcirg1/Matk/Kit/Cyba/Tns4/Atp6v0a1/Akt3/Esr1/Igf1/Flt4/Tns3/Thbs4/Prkcd/Atp6v1c1/Atp6v0c/Vegfb/Pdgfa/Nrp2/Stat1/Ptpn18/Lamc1/Spint1/Src/Sh2d2a/Hgf/Spp1/Dusp4/Mapkapk3/Trib3/Atp6v0b/Tlr9/Col6a3/Atp6v1a/Aph1c/Sphk1/Nrg1/Ctnnd1 | 39 |
| 2 weeks BCG/Nano-FP1 upregulated | R-MMU-451927 | Interleukin-2 family signaling | 0.0459143681881447 | Lck/Stat1/Il21/Il21r/Il2rg/Jak3/Il2rb/Il3ra | 8 |
| 2 weeks BCG/Nano-FP1 upregulated | R-MMU-114608 | Platelet degranulation | 0.0478848204544478 | Pdgfb/Vwf/Psap/Igf1/Ctsw/Vegfb/Cd63/Pdgfa/Cyb5r1/Ecm1/Hgf/Lgals3bp/Endod1/Orm1/Lhfpl2/Actn2/Serpina3f | 17 |
| 2 weeks BCG/Nano-FP1 upregulated | R-MMU-446193 | Biosynthesis of the N-glycan precursor (dolichol lipid-linked oligosaccharide, LLO) and transfer to a nascent protein | 0.0480007456286368 | Mvd/St6galnac1/Ctsa/Fuom/Neu3/Amdhd2/Glb1/Slc17a5/St6galnac2/Dolk/St6galnac4 | 11 |
| 2 weeks BCG/Nano-FP1 upregulated | R-MMU-418592 | ADP signalling through P2Y purinoceptor 1 | 0.0487853372264413 | Src/Gnb4/P2ry1/Gna15/Gng12/Gng2 | 6 |
| 2 weeks BCG/Nano-FP1 upregulated | R-MMU-977606 | Regulation of Complement cascade | 0.0494655986012909 | C3/C2/C1qa/C1qc/C1qb/C1s1/C3ar1/C1ra | 8 |
| 11 weeks BCG/Nano-FP1 upregulated | R-MMU-76002 | Platelet activation, signaling and aggregation | 4.31131545842681e-06 | Gna12/Pdgfb/Arrb1/Igf1/Itih4/Clu/F5/Selp/P2ry1/Ppbp/Pf4/Gp9/Gng11/Lgals3bp/Itga2b/Gng12/F13a1/Gp5/Serpina3f/Mpig6b/Gp6 | 21 |
| 11 weeks BCG/Nano-FP1 upregulated | R-MMU-114608 | Platelet degranulation | 0.00112473085202516 | Pdgfb/Igf1/Itih4/Clu/F5/Selp/Ppbp/Pf4/Lgals3bp/Itga2b/F13a1/Serpina3f | 12 |
| 11 weeks BCG/Nano-FP1 upregulated | R-MMU-76005 | Response to elevated platelet cytosolic Ca2+ | 0.00112473085202516 | Pdgfb/Igf1/Itih4/Clu/F5/Selp/Ppbp/Pf4/Lgals3bp/Itga2b/F13a1/Serpina3f | 12 |
| 11 weeks BCG/Nano-FP1 upregulated | R-MMU-380108 | Chemokine receptors bind chemokines | 0.00355306702290562 | Ppbp/Pf4/Cxcl9/Cxcl10/Cxcr6/Cx3cr1/Xcr1 | 7 |
| 11 weeks BCG/Nano-FP1 upregulated | R-MMU-1592389 | Activation of Matrix Metalloproteinases | 0.00355306702290562 | Mmp14/Mmp8/Timp2/Mmp9/Mmp25/Mmp2 | 6 |
| 11 weeks BCG/Nano-FP1 upregulated | R-MMU-140877 | Formation of Fibrin Clot (Clotting Cascade) | 0.00434161698081233 | F5/Pf4/Gp9/F13a1/Gp5/Cd177 | 6 |
| 11 weeks BCG/Nano-FP1 upregulated | R-MMU-416482 | G alpha (12/13) signalling events | 0.00434161698081233 | Gna12/Tiam2/Gng11/Gng12/Arhgef10l/Arhgef37/Arhgef15/Kalrn | 8 |
| 11 weeks BCG/Nano-FP1 upregulated | R-MMU-198933 | Immunoregulatory interactions between a Lymphoid and a non-Lymphoid cell | 0.00434161698081233 | Icam2/Treml1/Ifitm1/Ifitm3/Ifitm6/Ifitm2/Cd300lb/Pilrb1/H2-Q10 | 9 |
| 11 weeks BCG/Nano-FP1 upregulated | R-MMU-140875 | Common Pathway of Fibrin Clot Formation | 0.0240974787068585 | F5/Pf4/F13a1/Cd177 | 4 |
| 11 weeks BCG/Nano-FP1 upregulated | R-MMU-1474244 | Extracellular matrix organization | 0.0269271075481127 | Pdgfb/Mmp14/Icam2/Mmp8/Timp2/Mmp9/Mmp25/Emilin2/Sdc3/Itgam/Mmp2/Itga2b/Capn5/Col6a3 | 14 |
| 11 weeks BCG/Nano-FP1 upregulated | R-MMU-75892 | Platelet Adhesion to exposed collagen | 0.0349520949758006 | Gp9/Gp5/Gp6 | 3 |
| 11 weeks BCG/Nano-FP1 upregulated | R-MMU-202733 | Cell surface interactions at the vascular wall | 0.039077924149315 | Slc16a3/Sdc3/Selp/Pf4/Itgam/Cd177/Gm9733/Gp6 | 8 |
| 14 weeks BCG/Nano-FP1 downregulated | R-MMU-8953854 | Metabolism of RNA | 1.03789254157287e-07 | Hnrnpd/Puf60/Cwc15/Slbp/Hspb1/Hnrnpa2b1/Tpr/Gtf2h1/Hnrnph1/Ubc/Rps25/Ppil4/Mtrex/Srsf6/Psmd11/Snrpd3/Slu7/Ddx52/Ddx42/Ddx5/Polr2h/Snw1/Srsf5/Rps14/Smndc1/Ppil3/Cstf3/Nop56/Xrn2/Exosc9/Ddx20/Rps3a1/Syf2/Isy1/Mphosph10/Pqbp1/Cnot7/Rbm5/Tut4/Nup54/Lsm1/Sf3b6/Thoc2/Wdr43/Pcf11/Hnrnpf/Gnl3/Hnrnph2/Utp18/Hnrnpc/Rps7/Utp14a/Sarnp/Seh1l/Zc3h11a | 55 |
| 14 weeks BCG/Nano-FP1 downregulated | R-MMU-72203 | Processing of Capped Intron-Containing Pre-mRNA | 6.00348956974805e-06 | Hnrnpd/Puf60/Cwc15/Hnrnpa2b1/Tpr/Hnrnph1/Ppil4/Srsf6/Snrpd3/Slu7/Ddx42/Ddx5/Polr2h/Snw1/Srsf5/Smndc1/Ppil3/Cstf3/Syf2/Isy1/Pqbp1/Rbm5/Nup54/Sf3b6/Thoc2/Pcf11/Hnrnpf/Hnrnph2/Hnrnpc/Sarnp/Seh1l/Zc3h11a | 32 |
| 14 weeks BCG/Nano-FP1 downregulated | R-MMU-72163 | mRNA Splicing - Major Pathway | 3.99604534014971e-05 | Hnrnpd/Puf60/Cwc15/Hnrnpa2b1/Hnrnph1/Ppil4/Srsf6/Snrpd3/Ddx42/Ddx5/Polr2h/Snw1/Srsf5/Smndc1/Ppil3/Cstf3/Syf2/Isy1/Pqbp1/Rbm5/Sf3b6/Pcf11/Hnrnpf/Hnrnph2/Hnrnpc | 25 |
| 14 weeks BCG/Nano-FP1 downregulated | R-MMU-72172 | mRNA Splicing | 7.70875031215125e-05 | Hnrnpd/Puf60/Cwc15/Hnrnpa2b1/Hnrnph1/Ppil4/Srsf6/Snrpd3/Ddx42/Ddx5/Polr2h/Snw1/Srsf5/Smndc1/Ppil3/Cstf3/Syf2/Isy1/Pqbp1/Rbm5/Sf3b6/Pcf11/Hnrnpf/Hnrnph2/Hnrnpc | 25 |
| 14 weeks BCG/Nano-FP1 downregulated | R-MMU-109688 | Cleavage of Growing Transcript in the Termination Region | 0.0396204795214795 | Slbp/Srsf6/Snrpd3/Slu7/Srsf5/Cstf3/Thoc2/Pcf11/Sarnp/Zc3h11a | 10 |
| 14 weeks BCG/Nano-FP1 downregulated | R-MMU-73856 | RNA Polymerase II Transcription Termination | 0.0396204795214795 | Slbp/Srsf6/Snrpd3/Slu7/Srsf5/Cstf3/Thoc2/Pcf11/Sarnp/Zc3h11a | 10 |
| 14 weeks BCG/Nano-FP1 downregulated | R-MMU-6791226 | Major pathway of rRNA processing in the nucleolus and cytosol | 0.0396204795214795 | Rps25/Mtrex/Ddx52/Rps14/Nop56/Xrn2/Exosc9/Rps3a1/Mphosph10/Wdr43/Gnl3/Utp18/Rps7/Utp14a | 14 |
| 14 weeks BCG/Nano-FP1 downregulated | R-MMU-72312 | rRNA processing | 0.0396204795214795 | Rps25/Mtrex/Ddx52/Rps14/Nop56/Xrn2/Exosc9/Rps3a1/Mphosph10/Wdr43/Gnl3/Utp18/Rps7/Utp14a | 14 |
| 14 weeks BCG/Nano-FP1 downregulated | R-MMU-8868773 | rRNA processing in the nucleus and cytosol | 0.0396204795214795 | Rps25/Mtrex/Ddx52/Rps14/Nop56/Xrn2/Exosc9/Rps3a1/Mphosph10/Wdr43/Gnl3/Utp18/Rps7/Utp14a | 14 |
| 14 weeks BCG/Nano-FP1 upregulated | R-MMU-191273 | Cholesterol biosynthesis | 2.33979995802357e-06 | Fdft1/Sqle/Hsd17b7/Pmvk/Ebp/Msmo1/Sc5d/Lss/Dhcr24/Mvk/Idi1/Dhcr7/Hmgcs1 | 13 |
| 14 weeks BCG/Nano-FP1 upregulated | R-MMU-1280215 | Cytokine Signaling in Immune system | 0.000494178515525408 | Mapk7/Ebi3/Rps6ka1/Stx1a/Crlf1/Csf1/Plcg1/Fos/Tnfsf11/Psmb5/Tnfrsf17/App/Il10rb/Cd4/Psmb8/Csf1r/Tnfrsf25/Flnb/Il17a/Stat1/Tnfsf8/Tnfrsf1b/Psmb2/Tnfrsf4/Ltbr/Sh2b1/Il21r/Cd40lg/Tnfsf13b/Dusp4/Jak3/Tyk2/Uba7/Inppl1/Crlf2/Socs1/Stx3/Il17f/Tnfrsf14/Il17re/Ifng/Osm/Csf2ra/Irak4/Il2rb/Uba52/Il3ra/Il18bp/Il1rl2/Csf2rb/Csf2rb2/Il11ra1/Psme2b/Psmb9/Tnfsf12 | 55 |
| 14 weeks BCG/Nano-FP1 upregulated | R-MMU-1236974 | ER-Phagosome pathway | 0.000721014168889536 | H2-M3/H2-M2/Tapbp/Tap2/H2-Q4/Tap1/H2-Q7/H2-K1/H2-T23/H2-Q6/H2-D1 | 11 |
| 14 weeks BCG/Nano-FP1 upregulated | R-MMU-1236975 | Antigen processing-Cross presentation | 0.00165502086196775 | Cyba/Fcgr1/H2-M3/H2-M2/Psmb5/Itgb5/Tapbp/Psmb8/Tap2/Psmb2/H2-Q4/Tap1/H2-Q7/H2-K1/H2-T23/H2-Q6/H2-D1/Psme2b/Psmb9 | 19 |
| 14 weeks BCG/Nano-FP1 upregulated | R-MMU-71387 | Metabolism of carbohydrates | 0.00284160886305006 | Hk2/Naglu/Slc25a1/Sgsh/Man2b1/Pgam1/Galns/Aldoc/Calm3/Pfkl/Hexb/Tpi1/Papss2/Hexa/Gusb/Gaa/Sdc3/Slc25a10/Hk3/Akr1a1/Slc9a1/B3gnt3/Pgls/Slc37a2/Cspg4/Idua/Gns/G6pc3/Hpse/Galt/Hk1/Chpf2/Pck2/Agrn/Glb1/Nup188/Ndst1/Uba52 | 38 |
| 14 weeks BCG/Nano-FP1 upregulated | R-MMU-983170 | Antigen Presentation: Folding, assembly and peptide loading of class I MHC | 0.00395305955794156 | H2-M3/H2-M2/Tapbp/Tap2/H2-Q4/Tap1/H2-Q7/H2-K1/H2-T23/H2-Q6/H2-D1 | 11 |
| 14 weeks BCG/Nano-FP1 upregulated | R-MMU-1236977 | Endosomal/Vacuolar pathway | 0.00494545118233424 | H2-M3/H2-M2/H2-Q4/H2-Q7/H2-K1/H2-T23/H2-Q6/H2-D1 | 8 |
| 14 weeks BCG/Nano-FP1 upregulated | R-MMU-8957322 | Metabolism of steroids | 0.00859350008673834 | Stard3nl/Rxra/Acot8/Ptgis/Abcc3/Lgmn/Fdft1/Sqle/Srebf2/Vdr/Hsd17b7/Pmvk/Osbpl3/Ebp/Msmo1/Sc5d/Lss/Dhcr24/Mvk/Osbpl1a/Idi1/Dhcr7/Hmgcs1 | 23 |
| 14 weeks BCG/Nano-FP1 upregulated | R-MMU-2024096 | HS-GAG degradation | 0.00859350008673834 | Naglu/Sgsh/Gusb/Sdc3/Idua/Hpse/Agrn/Glb1 | 8 |
| 14 weeks BCG/Nano-FP1 upregulated | R-MMU-166663 | Initial triggering of complement | 0.00871684469795211 | C3/C2/C1qa/C1qc/C1qb/C1ra/C4b | 7 |
| 14 weeks BCG/Nano-FP1 upregulated | R-MMU-449147 | Signaling by Interleukins | 0.0113223791946206 | Mapk7/Ebi3/Rps6ka1/Stx1a/Crlf1/Csf1/Fos/Psmb5/App/Il10rb/Cd4/Psmb8/Csf1r/Il17a/Stat1/Psmb2/Il21r/Dusp4/Jak3/Tyk2/Inppl1/Crlf2/Socs1/Stx3/Il17f/Il17re/Osm/Csf2ra/Irak4/Il2rb/Uba52/Il3ra/Il18bp/Il1rl2/Csf2rb/Csf2rb2/Il11ra1/Psme2b/Psmb9 | 39 |
| 14 weeks BCG/Nano-FP1 upregulated | R-MMU-198933 | Immunoregulatory interactions between a Lymphoid and a non-Lymphoid cell | 0.0165134807611225 | H2-M3/H2-M2/Trem2/C3/Siglec1/Pianp/Cd40lg/Cd300a/H2-Q4/Slamf7/Cd8a/Lair1/H2-Q7/H2-K1/Hcst/H2-T23/H2-Q6/H2-D1 | 18 |
| 14 weeks BCG/Nano-FP1 upregulated | R-MMU-5669034 | TNFs bind their physiological receptors | 0.0225303096690408 | Tnfsf11/Tnfrsf17/Tnfrsf25/Tnfsf8/Tnfrsf1b/Tnfrsf4/Tnfsf13b/Tnfrsf14 | 8 |
| 14 weeks BCG/Nano-FP1 upregulated | R-MMU-1660662 | Glycosphingolipid metabolism | 0.0240261360045511 | Gm2a/Psap/B4galnt1/Ctsa/Galc/Hexb/Hexa/Gba/Asah1/Glb1 | 10 |
| 14 weeks BCG/Nano-FP1 upregulated | R-MMU-5668541 | TNFR2 non-canonical NF-kB pathway | 0.0240261360045511 | Tnfsf11/Psmb5/Tnfrsf17/Psmb8/Tnfrsf25/Tnfsf8/Tnfrsf1b/Psmb2/Tnfrsf4/Ltbr/Cd40lg/Tnfsf13b/Tnfrsf14/Uba52/Psme2b/Psmb9/Tnfsf12 | 17 |

ID= Reactome pathway ID. P.adjust = adjusted p-value; indicates the significance of the enrichment. Count = number of genes from the submitted list found in the pathway.

**Table S.5. List of primers used in the RT-qPCR for some of the DE genes analyzed.**

| **Gene name** | **Forward primer (5’-3’)** | **Reverse primer (5’-3’)** |
| --- | --- | --- |
| Nos2 | CTTTGCCACGGACGAGAC | AACTTCCAGTCATTGTACTCTGAGG |
| H2M2 | GACAGTGGGAAATGAGACTCTACTG | CATGATGATGTCAGAAGGGTAGAA |
| Trbv16 | GTGAAGCCACTGCCTCATCT | TGGATGACACCAGCATTTGT |
| Itgam | TGTCCCTGGCTGTTTCTACTG | ATTCTCCTTGCAGTTTTGGTG |
| Cd38 | AAGATGTTCACCCTGGAGGA | ACTCCAATGTGGGCAAGAGA |
| Htra1 | CATTGAAGTCATTCCTGACACC | TGTCCGTTGATGCTGATGA |
| Cspg4 | GCAGAGGAGGTCTTGGTGAA | GGACATCTCGTGCTCATACAGA |
| Ctsk | AGCGAACAGATTCTCAACAGC | AGACAGAGCAAAGCTCACCAT |
| Bok | AGTGGCAGGCCACATCTT | CCACGGAATACAGGGACACTA |
| F7 | TGCTGCTTCTCTGCTTTCTG | TGCTTCCTCCTGGGTTATGA |
| Mlph | ACAACAGCTGTGCCCTCTCT | TCAAAAGAACCACTGTCTACGC |
| Gmpr | CGCGGACCTTAAACTCGAC | GTAAAAGTTCGCTCAAGATCCAC |
| Ccl17 | GCTCTGCTTCTGGGGACTTT | GAATGGCCCCTTTGAAGTAA |
| Cdo1 | GATTCTGTGCTGGGGTGAA | CAGTGGGAGTCCGTGTGAT |
| Ms4a7 | ATCTCAACCAAACCCTTTGC | GGCAACAACAGAGCTTGCTA |
| Car4 | CAAACCAAGGATCCTAGAAGCA | GGGGACTGCTGATTCTCCTT |
| Cox6b2 | CCATTCACTGTGTCCCATCA | AGCCACTAGGGGTTGATGAC |
| Actb | CTAAGGCCAACCGTGAAAAG | ACCAGAGGCATACAGGGACA |
