# Supplementary figures and images for: Changes in the Immune Phenotype and Gene Expression Profile Driven by a Novel Tuberculosis Nanovaccine: Short and Long-Term Post-immunization
Source: Front Immunol. 2021 Jan 28;11:589863. doi: 10.3389/fimmu.2020.589863 (PMC7876410; doi:10.3389/fimmu.2020.589863)

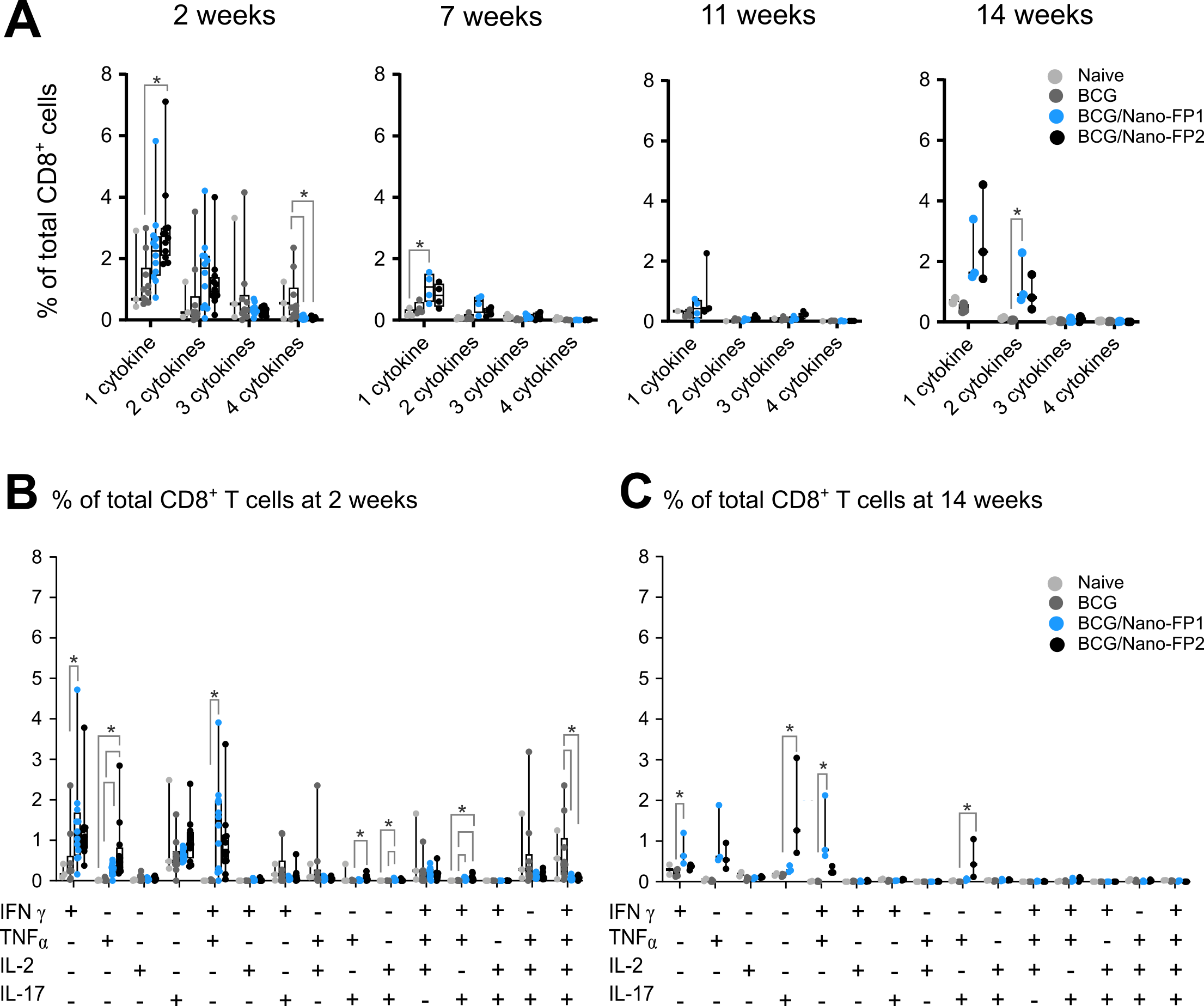

Supplement: Supplementary file 4 [file Image_3.tiff]

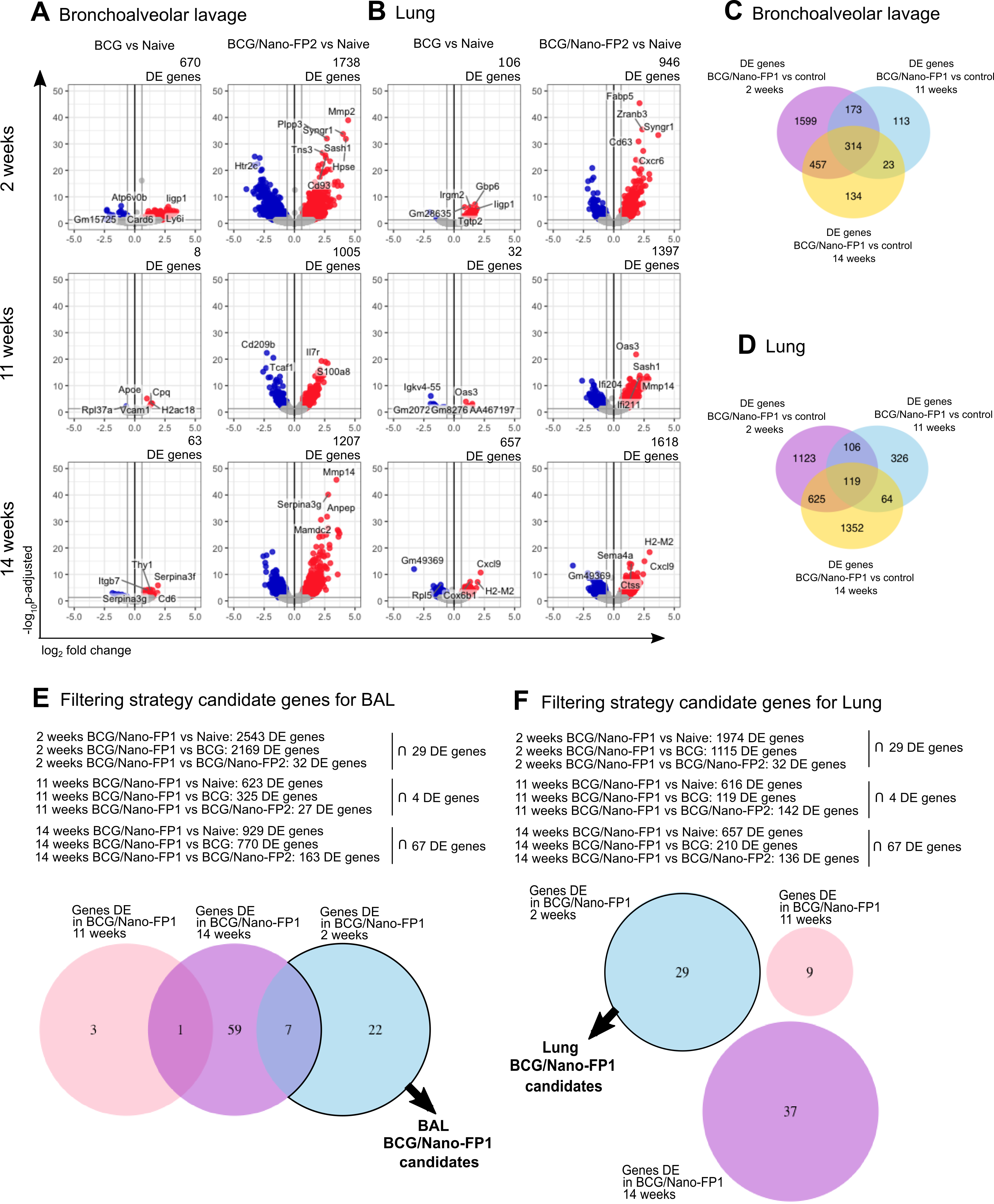

Supplement: Supplementary file 5 [file Image_4.tiff]
